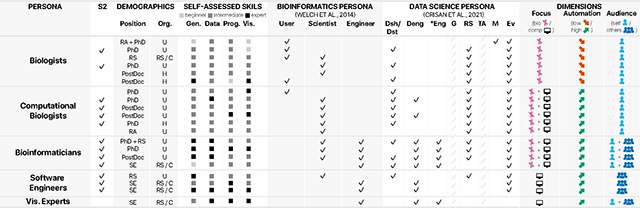# Understanding Visualization Authoring Techniques for Genomics Data in the Context of Personas and Tasks

**DOI:** 10.1109/TVCG.2024.3456298

**Published:** 2024-12-03

**Authors:** Astrid van den Brandt, Sehi L’Yi, Huyen N. Nguyen, Anna Vilanova, Nils Gehlenborg

**Affiliations:** Eindhoven University of Technology; Harvard Medical School; Harvard Medical School; Eindhoven University of Technology; Harvard Medical School

**Keywords:** User interviews, visual probes, visualization authoring, genomics data visualization

## Abstract

Genomics experts rely on visualization to extract and share insights from complex and large-scale datasets. Beyond off-the-shelf tools for data exploration, there is an increasing need for platforms that aid experts in authoring customized visualizations for both exploration and communication of insights. A variety of interactive techniques have been proposed for authoring data visualizations, such as template editing, shelf configuration, natural language input, and code editors. However, it remains unclear how genomics experts create visualizations and which techniques best support their visualization tasks and needs. To address this gap, we conducted two user studies with genomics researchers: (1) semi-structured interviews (*n*=20) to identify the tasks, user contexts, and current visualization authoring techniques and (2) an exploratory study (*n*=13) using visual probes to elicit users’ intents and desired techniques when creating visualizations. Our contributions include (1) a characterization of how visualization authoring is currently utilized in genomics visualization, identifying limitations and benefits in light of common criteria for authoring tools, and (2) generalizable design implications for genomics visualization authoring tools based on our findings on task- and user-specific usefulness of authoring techniques. All supplemental materials are available at https://osf.io/bdj4v/.

## 1 INTRODUCTION

Genomics experts heavily rely on data visualization for the sensemaking of complex data. The importance of visualization in the sensemaking process is apparent in the wide variety of visualization tools for genomics data analysis [52]. Typically, these tools provide off-the-shelf solutions tailored to support analysis tasks. Because of the highly collaborative nature and the diversity of questions to be addressed, genomics experts also engage in visualization authoring activities. These activities include the design and implementation of data visualizations, both for the goal of data exploration and communication of data insights. While visualization for genomics data exploration has been extensively studied [39,42,49,52], there has been little research into the authoring practice of these domain experts. Visualization authoring generally refers to the process of transforming data to visual representations, and it is recognized as a non-trivial task [24,25]. In visualization research, various techniques have been studied and proposed for interactively authoring visualizations, which all present trade-offs related to authoring criteria [3] and task effectiveness [56, 57]. For example, template-based editors, such as Microsoft Excel’s Recommended Charts [50], enable easy and efficient construction of visualizations. However, these often result in constrained outcomes and thus are at odds with *expressiveness*—the scope of possible design choices enabled (“Can I build it” [9]) [55]. On the other side of the spectrum are programmatic approaches such as Vega-Lite [62] and D3 [10], which are highly expressive but pose a steep learning curve [61], affecting their *learnability* (“Do I know how?” [9]). The techniques also differ in suitability for specific lower-level authoring tasks [47,56]. For example, template-based editors are good for quickly creating an initial scaffold for the visualization, but further customizations are likely cumbersome or even infeasible. Techniques using natural language, on the other hand, are promising for making expressive customizations [77].

User context and skills influence the authoring process and techniques used. A study about interactions with voice user interfaces (VUI) showed that user characteristics influence how well users could adopt the interaction technique [51]. Previous studies about the analytical workflows of data scientists have also revealed that user and contextual characteristics impact the type of tooling they need [18]. Like data scientists, genomics experts work in different organizations, from academic institutions to clinical and experimental labs and companies, with broadly diverse backgrounds and skill sets in programming, data manipulation, and visualization. Therefore, the question arises: **What visualization authoring techniques can best support a diverse group of genomics experts and in which tasks?**

Understanding genomics data visualization authoring is of interest to the broader visualization and CS communities, as both the complexity of genomics visualizations—often featuring advanced visualization techniques like semantic zooming, hierarchical structures, and interconnected relationships—and the difficulties posed by complex data, such as handling large datasets across multiple levels of detail and managing uncertainty, challenge the expressiveness capabilities and mirror integration needs seen in other data-intensive fields.

In this paper, we aim to address this gap in understanding by investigating the current workflows and challenges of diverse genomics experts for authoring data visualizations. Going beyond, we further explore how to address domain experts’ challenges by eliciting their intent for visualization authoring when introduced to various authoring techniques outside their current practices. We had a two-fold approach to addressing this question. First, we conducted semi-structured interviews with a diverse group of genomics experts (*n*=20) to broadly understand their authoring tasks and whether and how user context and characteristics impact authoring processes and decisions. Inspired by Crisan et al. [19], we then designed visual design probes using our newly acquired knowledge of the current situation for exploratory elicitation sessions (*n*=13) to gain deeper insights into their intent for authoring. We discuss the challenges and benefits of authoring techniques in light of common criteria for evaluation: *effectiveness, efficiency, expressiveness, integration, learnability, and usability* [3,25,42]. Together, the two formative studies lead to the following contributions:

- A characterization of the current challenges in genomics visualization authoring, stratified across five distinct authoring personas that extend bioinformatics personas [78] and data science personas [18];
- Delineation of task- and user-specific usefulness of six common visualization authoring techniques; and
- Design implications for visualization authoring tools of genomics data visualizations.

Finally, we provide comprehensive data from our studies in the Supplementary Material (doi:10.17605/osf.io/BDJ4V), which is available under the CC-BY 4.0 license.

## 2 BACKGROUND AND RELATED WORK

We summarize prior work in visualization authoring, task taxonomies, and formative studies in visualization and human-computer interaction.

### 2.1 Visualization Authoring Tasks

Previous studies describing visualization authoring systems offer varying scopes on what constitutes the task of visualization authoring. For example, some define it as having a narrower scope than visualization design, focusing on the implementation of predefined design using already formatted data [5,61], while others define it broadly, including transforming data and exploring design choices [73,75]. In this paper, we use the term *visualization authoring* broadly, as the design and implementation of data visualizations, involving decisions on data, visual encodings, and composition. Users engage in various lower-level tasks as part of visualization authoring. We build on previous visualization reference models [14,15], visualization taxonomies [11,28], and related work that contributes assertions and task classifications [38,57,77], to outline the following tasks in genomics data visualization authoring.

#### Data Transformation:

Data is loaded, cleaned, and transformed into a structured format suitable for visualization, including filtering, aggregations, and calculations. Related tasks in prior work include transform data [14], introduce→import (arrow indicates the hierarchy of tasks) [11], and filtering change [38].

#### Visual Mapping and View Creation:

The former refers to the mapping of processed data to visual variables, such as position, size, shape, and color. The latter identifies with the view transformation step, where the initial visual representation or structure is created, involving the spatial layout, scaling, and clipping. Related tasks are: encoding operations [77] and data and view specification [28].

#### Customization:

This task involves small chart edits, enhancements, or superficial changes to the visual design to improve its readability, interpretability, or aesthetic appeal (including titles, fonts, color choices, axis ticks/labels, swapping the horizontal/vertical axes, and distortions). Related tasks in previous work include manipulate→select [11], mark operations [57], and view manipulation [28].

#### Modification:

This task refers to significant chart edits involving changes in chart type and additional data encoding to extend the current design of one view. Related tasks are manipulate→change [11], and encoding change [38].

#### Coordinated Multiple Views and Interactions:

The task identifies significant chart edits to extend the current design to multiple views with interaction. Tasks in prior work include view transformation [14], interactivity [14], and manipulate→arrange [11].

#### View Arrangement:

Changing the arrangement of views to enhance clarity, facilitate comparisons, or tailor the presentation to specific audience needs or preferences. Corresponding tasks in previous work include arrange change [38] and layout operations [77].

#### Glyph Creation and Annotation:

This task involves data-driven glyph creations, which can be enhanced by annotations. Related tasks in previous work include introduce→annotate [11], introduce→derive [11], annotate operations [77], and process and provenance [28].

### 2.2 Visualization Authoring Techniques

Existing literature delineates a broad spectrum of techniques and tools available for authoring effective visualizations [24, 48]. From these studies, we extracted six techniques: template-based, shelf configuration, natural language input (NLI), code editing, visualization by demonstration (VbD), and example-based. These techniques can be used in an independent manner or in combination to carry out specific tasks in visualization construction. On the less technical, more intuitive side of the spectrum, (1) template-based techniques provide standard chart types in pre-defined templates of existing authoring tools [33, 46, 47, 75]. However, such techniques typically offer limited customization options. (2) Example-based techniques are a related approach where the user submits an example, and the system provides a reusable template for further use, as demonstrated with AutoGosling [76]. (3) Shelf configuration provides users more flexibility in creating bindings between data and visual encoding, often with the use of drag-and-drop interactions. While the template approach is mostly employed in the visualization creation and encoding mapping tasks, shelf configuration can go beyond that to enable fine-grained alternatives between different encodings [13,54,60,75]. (4) Visualization by demonstration (VbD), including direct manipulation (DM) and sketching, allows users to edit visualizations by directly manipulating—using mouse or pen—the graphical encodings used in the visual representation [56,58]. In VbD, the intent of a user is interpreted by the system, which in turn recommends potential mappings. VbD is often coupled with other techniques such as NLI [67], or with GUI controls [57]. Compared to VbD, the recommendation step is lacking in DM [21,35,37,68,69]. Towards the more technical side of the spectrum are (5) natural language interfaces (NLI) and code editing. Visualization-oriented NLIs [65] take a user prompt in natural language as input and to facilitate the creation of a visualization, chart customizations, adding annotations [21,68,71,77], and sometimes also capabilities for data question and answering and exploration [63,69,70]. On the technical extreme of the spectrum, (6) code editing enables expressivity in various ways and typically accommodates all stages in the authoring process, from data transformation to visual creation, building coordinated views, and interaction [62]. Some approaches specialize for certain tasks or scenarios, for example design [8] or chaining between different tools [29], or use in computational notebooks [82].

Despite being provided access to existing tools and techniques, it remains unclear how users in the genomics domain perceive lower-level tasks and their specific roles, as well as the extent to which these tools’ capabilities can be effectively applied. The genomics research field poses cutting-edge data questions with the data being large and heterogeneous, leading to the need for integration from different data sources and undergoing analysis prior to viewing. Current tools are mainly in the graphical user interface (GUI) and command-line interface (CLI) formats, some of which only provide limited template editing options [40]. Common data visualization tools used for genomics data under the Grammar of Graphics (GoG) [80] concept include ggplot2 [79], Gosling [40] toolkit for scalable and interactive genomics visualization, and Gos [44] Python library. GenoREC [53] presented visualization recommendations specific to users’ data and tasks. AutoGosling [76] allows users to recreate genomics data visualizations from an input image or sketch. While the current body of work provides solutions with a template or coding approach, there is a lack of available techniques that foster visualization authoring. Our study aimed to understand the authoring process from users’ perspectives and provide insights that can guide future research in this field.

### 2.3 Formative Studies to Investigate Users and Context

This section presents a brief background on formative user interviews and elicitation approaches that inspired our work.

#### Interviews and Personas.

Based on semi-structured interviews, Kandel et al. [34] presented the results of a study with 35 data analysts that emphasized the importance of visual analytic tools to improve the analysis quality. Following a similar strategy, Wongsuphasawat et al. [81] shifted the focus to better exploratory data analysis. Crisan et al. [17] characterize the use of AutoML in enterprise settings. Furthermore, Crisan et al. [18] presented a synthesized model of data science work and proposed nine distinct roles among data scientists with regard to their expertise. On the design aspect of visualization, Bigelow et al. [7] reflected on how designers design with data, based on an observational study and interviews. The work of Bako et al. [4] shed light on the ways in which visualization designers use examples and how computational tools can assist these practices also in a semi-structured interview manner. Aiming to better understand how visualization novices construct visualization, Grammel et al. [25] suggested that tools should aid in data selection, provide explanations, and support learning.

#### Probes.

Probes are materials encouraging people to reflect on and share their experiences, feelings, and attitudes [23]. They can be seen as a postcard without a message, to provoke a reaction and elicit inspiration from that. Compared to other elicitation methods such as generative toolkits where users actively participate in the design (“designing with”), probes are a “designing for” approach, meaning that the designer or researcher uses the elicited responses at their own discretion [59]. In a study in healthcare, Mamykina et al. [43] use a probe to engage individuals in reflective reasoning about their health to highlight the potential strategies, biases, or misconceptions. Recently, Crisan et al. [19] used probes to elicit users’ preferred interactions for interactive machine learning systems.

Despite the substantial research on authoring behaviors, user personas, and contexts, no studies investigated these questions within genomics research. Inspired by these formative studies, we combine interviews with design probes to understand the user context and elicit richer, more nuanced information into visualization authoring needs and practices of genomics researchers.

## 3 THE CURRENT PRACTICE OF AUTHORING BY A DIVERSE AUDIENCE (STUDY 1)

Our goal of the first study was to understand how a diverse group of experts in genomics currently create and use data visualization in their workflows. Specifically, we aimed to gain insights into authoring tasks, tools used, challenges faced, and how user and context characteristics influence approaches to visualization authoring.

### 3.1 Participants

We intended to reach experts with diverse backgrounds and skill sets. Multiple channels were used to recruit participants, such as university and computational biology conference mailing lists, X (formerly, Twitter), Slack, Meetup communities, printed flyers on bulletin boards, and direct emailing within our network. All participants received a $25 Amazon gift card for their participation in the study. Prior to the interview session, we conducted a pre-study survey to capture information on their current position, years of experience, frequency of visualization authoring, and self-reported skills in genomics data analysis, data processing, programming, and visualization. We also requested participants to upload two representative examples of visualizations they had authored to verify their study qualifications (e.g., familiarity with genome-mapped data visualizations [40] that show the mapping to genomic coordinates), and to guide our interview questions.

We recruited 20 experts from 9 different organizations. They were affiliated with universities (*n*=12), research centers and companies (*n*=5), and hospitals and clinical research facilities (*n*=3). Interviewees self-identified as PhD students, PostDocs, Software Engineers or Scientific Programmers, Research Scientists, Research Assistants, Master Students, and Bioinformaticians. Their years of experience in genomics was 1–3 years (*n*=9), 6 years or more (*n*=7), >3–5 years (*n*=2), and less than 1 year (*n*=2). They visualized genomics data weekly (*n*=6), daily (*n*=5), monthly (*n*=4), bi-weekly (*n*=3), and less than monthly (*n*=2). According to participants’ self-assessment, they had varied expertise levels in genomics data analysis (2 beginners, 12 intermediates, and 6 experts), data processing (12 intermediates and 6 experts), programming (2 beginners, 7 intermediates, and 3 experts), and visualization (2 beginners, 12 intermediates, and 6 experts). Table 1 summarizes participant demographics and self-identified skills.

### 3.2 Procedure

Our semi-structured interviews were conducted in a pair-interview setup [2]. Three paper authors took on the roles of driver and navigator, keeping the driver consistent for each interview, but where needed, navigators alternated. Driver and navigators have all participated in the analysis of the interview results. The driver and navigators also had a pre-interview briefing before each session to discuss and align expectations. We conducted all interviews remotely using Zoom videoconferencing software. At the start of the interview, participants were briefed on the study’s aims. We asked for their consent to use Zoom transcription and save the chat history. One of the interviewers scrubbed the automatic transcripts of identifiable information. During the interviews, we also took notes to back them up. The interviews lasted between 50 and 90 minutes. We started each interview with a brief introduction round and open questions regarding the participants’ typical work week. We continued to the uploaded visualization examples and asked the participant to walk us through the authoring process. To help the participants delineate the authoring process and reflect on their choices, we selected questions from our interview guide that focused on topics drawn from prior literature and user studies. These topics included visualization authoring processes, user roles in workflows, and criteria for evaluating tools [3,4,17,25]. The following are the topics with example questions:

- *Goals:* What was the goal of creating this visualization?
- *Ideation Process:* How did you get an idea of what to visualize?
- *Workflow and Tasks:* Can you describe the step-by-step process you follow in creating the visualization(s)?
- *Tools*: What tools do you use, and what are their limitations (if any) in the process?
- *Challenges:* What are the major obstacles you encounter while creating the visualization?
- *Design Choices:* How often do you consider [reusing elements from previous designs / interactivity / … ]?
- *Context and Collaboration:* How do you collaborate with others during the development of visualization?
- *Reflection on Process:* Without any practical limitations or constraints, how would you envision the ideal construction process?

We piloted interviews with two participants to test the guide and interview dynamics, revealing the need for a concise checklist to cover all critical steps and track questions. Furthermore, we streamlined the guide by reducing question options for better navigation.

### 3.3 Analysis

We analyzed the interview data using an iterative hybrid approach, starting with loosely defined codes from qualitative content analysis [31] followed by open and axial coding [12], with two coders coding the 20 interviews (*ATLAS.ti Scientific Software Development GmbH. (2024). ATLAS.ti Mac (version 24.0.1)*). The process was realized in three steps.

(1) We started with a code calibration, where both coders used predefined codes from our literature-informed interview guide and openly coded any additional factors on 20% of the transcripts to familiarize themselves with the data. (2) After discussing the discrepancies in this pilot coding, the remaining data was iteratively coded, and codes were added, dropped, or updated based on weekly discussions. (3) Finally, one coder merged and consolidated the codes of each coder, resulting in 18 category codes for the final analysis. We used these codes and their co-occurrences to clusters insights. In the following section, we discuss our insights with representative quotes to support our claims.

We further created personas to represent the diverse users in genomics data visualization authoring. To identify these personas, we used interview observations and users’ self-identified skills, as well as their mapping to data science personas [18] and bioinformatics personas [78], following the approach established in previous work [18]. This helped to situate the personas within existing research and provided additional context about their typical work activities and responsibilities. Below, we describe the authoring personas that we encountered. We then describe shared and distinctive patterns in the tasks they perform, the tools they use, and the general processes they take.

### 3.4 Insights

#### 3.4.1 Users and Context

Based on the interviews, we found that genomics experts exhibit diverse types of visualization authoring processes and tool usage. The criteria mentioned, such as expressiveness or efficiency, also exhibit variations across different groups of users. Recognizing this diversity, we identified five personas that describe distinct groups of genomics data visualization authors: “Biologists”, “Computational Biologists”, “Bioinformaticians”, “Software Engineers”, and “Visualization Experts”. In discussing the diverse user characteristics and context, three dimensions emerged that guided the identification of these personas (see the last column in Table 1): (1) *Focus:* Is the bulk of their work focused on a biological question or a computational question, or both? (2) *Automation:* Is the level of automation in their analyses low or high? (3) *Audience:* Do they author visualizations for themselves or for others?

1. **“Biologists”** focus on biological questions, use a low level of automation, and author visualizations primarily for themselves.
2. **“Computational Biologists”** focus on both biological and computational questions, use a high level of automation, and author visualizations primarily for themselves.
3. **“Bioinformaticians”** focus on both biological and computational questions, use a high level of automation, and author visualizations for both others and themselves.
4. **“Software Engineers”** focus on computational questions, use a high level of automation, and author visualizations for others.
5. **“Visualization Experts”** focus on computational questions, use a high level of automation, and author visualizations for both others and themselves.

We are aware that these labels of abstraction are not precise representations of the real world. However, in the context of this study, they represent acceptable approximations that align well with different combinations of the “User”, “Scientist”, and “Engineer” bioinformatics personas [78] and introduce further nuances. It is also important to mention that the personas are not mutually exclusive, as individuals can wear different hats at different times. Some groups need more research; for example, we are aware that “Visualization Experts” is an outlier in this study and is overall quite distinct from the other groups.

#### 3.4.2 Goals and Motivations for Authoring Visualizations

All participants used visualizations for multiple goals in their workflow. It was almost always for data exploration or validation *“spot checking”*, as well as communication of data insights. Usually, these exploration and data validation stages happened at the beginning, after which a context switch between multiple programs was typically needed to customize a publication figure for presentation purposes near the ending phase of their analysis cycle. In some cases, participants also referred to visualization as a way of documenting and tracking their analysis process, and those visualizations would be authored in different ways: *“These [visualizations] were just for our own documentation. We were not planning to publish them or anything.”* (P1). Furthermore, P6 shared *“if I want to kind of crystalize and make it more permanent and presentable, then I’ll go make these types of visualizations.”*

##### Importance and Attitude.

Visualization was generally found to be important in participants’ workflows, and they had expressed goals to improve the authoring process, for example, by learning more expressive code editing techniques (P4, P8, P13). Some saw visualization primarily as necessary to communicate with others and thought it was not always needed to get quick insights about aspects of their data: *“Personally, when I’m deep into analysis, I don’t really visualize that for myself because I’m so deep in the task that just looking at tables and looking at numbers is good enough. But when I’m sort of finishing up things, I like to visualize stuff”* P8 said, and further clarified *“or when it becomes too much data.”*, indicating a nuanced approach.

#### 3.4.3 Tasks and Creation Process

##### Data Preparation and Transformation.

Tasks preparing the data for visualization were often remarked upon for various reasons. A common challenging task for almost all participants was integrating and loading data stored somewhere else into the visualization, hindering the integration of authoring steps in the workflow. Converting genomic data files and tidying operations were also highlighted as demanding by a few participants (P6, P3, P15). Conversion was often needed to move data between tools. One participant reflected on the lack of support in this task: *“There’s very common pat terns that people keep coding themselves […]. That could be a thing to have [support for] […] People often take this type of data and convert it to this [tidy data].”* (P6).

##### Customizations and Aesthetics.

Most participants expressed that they were making visual encoding and stylistic changes as a customization step in their authoring process. These customizations were regarded as highly important mainly for communication purposes but sometimes also for personal fulfillment. Participants often mentioned that aesthetics as part of the customizations were needed so *“people take it more seriously”*, as well as *“getting the message across”*, i.e., referring to expressiveness and effectiveness. For example, *“To be honest, if we look at like Nature [or] Cell papers, I think the more impactful papers have better visualizations. So I think It’s also very important that you keep up to this standard, right?”* (P12).

In contrast to making a preliminary visualization for quick exploration, *“getting the final version is hard”* (P4). What made this task challenging for some participants was their *“lacking of an eye for aesthetics”* (P4, P18), often referring to having difficulty with making sensible color choices. Other challenging customization tasks were alignment or composition adjustments or deciding how additional annotation information has to be shown without compromising other parts. Some felt like they were unable to do *“self-feedback”*, or found tools not supportive of making expressive edits, therefore *“sticking to the basics”* (P18). Often, participants used Adobe Illustrator as a way to create more expressive visualizations, but they experienced efficiency and learnability barriers: *“I have to adjust everything in Adobe Illustrator again. That is a significant amount of time.”* (P12). Also, P16 stated *“I have to do experiments, and there’s only 24h in a day. I can’t spend 4 years getting a graphic design degree to learn Adobe Illustrator.”*

Different from those who regarded the customizations as important authoring decisions, a few participants (P1, P2, P8) commented that they felt like they did not author anything but just *used* visualizations. For example, *“I’m not doing any visualizations myself […] I just try to use all the tools available and see if there’s something I like.”* (P8).

##### Getting Ideas and Creativity.

Authoring visualizations was often done with an idea for a design already in mind, but not always did participants know upfront how to visualize their idea. Quite a few of participants *(8/20)* mentioned that they had encountered both occurrences of known and unknown starting points. For example, *“I would maybe say 70% of the time I know what I need, because for that level of data I am looking at, I know what is informative, but there is definitely like 30% of cases where I don’t know how to best effectively visualize it. And probably it’s 100% of cases where the design we use could be improved.”* (P2).

Some participants (P6, P13, P19) used sketching to get and explore ideas. They also referred to drawing as a way to break down the authoring process into smaller tasks *“laying it out”*. For example: *“I think it’s like a very useful part of the process, because sometimes I’ll feel kind of stuck with all this data […] trying to figure out how to even present it. So just having a thing in front of you to play with then will allow you to give feedback to yourself and iterate.”* (P6).

##### Using Examples.

One frequently mentioned strategy to get started with visualizing is to use examples. Participants very often knew about or actively searched for examples from papers that were related to their work. Another commonly used strategy was to use search engines (e.g., Google) or Q&A forums (e.g., Stack Overflow) for visual and code examples, and sometimes even additional recommendations by these tools: *“I think what happens at times is I really like Google’s suggested images. I’ll Google a figure that I’m kind of interested in and I’ll see like related ones on the sidebar.”* (P4).

Participants also used specific catalogs or galleries, such as awesome-genome-visualization [20], for getting ideas as well as finding tools for implementation. Finally, some participants reflected on the use of large language models as ways to get inspired: *“ If I’m looking for some simple idea to process data before the visualization, then ChatGPT is useful. It’s also useful for simple visualizations. […] I suppose you could ask [ChatGPT] for ideas of how to visualize things too.”* (P19).

##### Brainstorming.

Some participants brainstormed by themselves and also involved others’ feedback as a strategy to *“sharpen ideas”* and improve on them. For example: *“Sometimes, it [a source of inspiration] is my own creativity, [for example] sometimes you just think: oh, this is not good enough, this is not clear enough. So how can I improve it? And then you just think [by yourself]. Or you discuss with other people.”* (P12). *“You talk with colleagues or something. Brainstorm. Come up with different ways to explore the data.”* (P19).

#### 3.4.4 Tools and Techniques Used in the Process

##### Tools and Techniques.

We discovered 83 different tools and programming languages discussed during the interviews. These included genome browsers, plotting libraries and applications, data manipulation libraries and tools, presentation software, vector graphics software, online resources, communication platforms, workflow tools, cloud service tools, deployment tools, and file management tools. We can broadly categorize the interface type into five groups: graphical user interface applications using windows, icons, menus, pointer input (GUI), command line interface applications with sometimes also graphical input (CLI+GUI), graphical interfaces with template selections (GUI+template), graphical interfaces with direct manipulation (GUI+DM), and code editors. All genomics experts mentioned the use of code editors, mostly Python or R, and frequently used them in computational notebooks, such as Jupyter Notebooks [36]. Furthermore, almost everyone mentioned using presentation or vector graphics software (GUI+DM). Almost all genomics experts *(19/20)* mentioned using genome browsers, such as IGV [72], which are GUI+template.

##### Use of Tools in the Process.

**Fig. 1:**
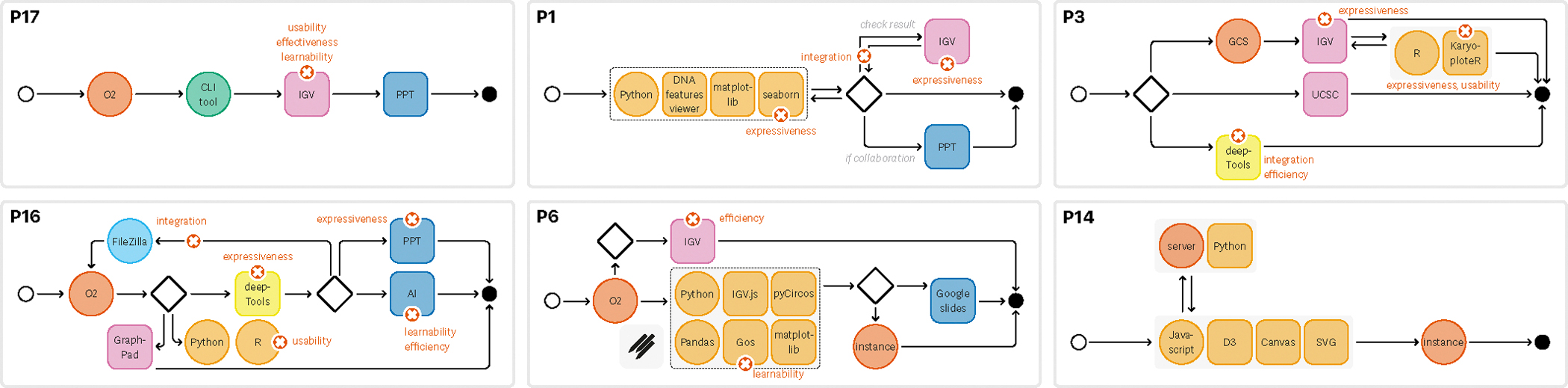
Representative workflows for each of the 5 personas: *“Biologists” (P16, P17), “Computational Biologists” (P1), “Bioinformaticians” (P6), “Software Engineers” (P3), and “Visualization Experts” (P14)*. Workflow diagrams for all participants can be found in the Supplementary Material at doi:10.17605/osf.io/BDJ4V.

We observed several combinations of the number of tools used and how they were connected to one another in the authoring process (Fig. 1). All participants chained three or more types of tools to create visualizations; most of the time *(18/20)*, iterations were needed. Typically, these iterations happened because of additional data loading, preparation, or transformations, e.g., *“filter and visualize”* (P7, P9, P11, P17). In a few cases (P2, P17, P20), the iterative pattern and the number of tools were much less pronounced and rather linear. Some others stated that they were looking for ways to create linear workflows for efficiency, for example, *“Ideally, you want it to be linear, right? Go in a straight line; that’s very efficient. But practically, that almost never happens.”* (P18).

Many participants (P4, P9, P12, P13, P14, P16, P18) commented about getting feedback on the created visualizations resulting in iterations. Participants also discussed using iterative approaches to develop or refine a design. One participant explained: *“…plotting - changing - plotting - changing… plotting things in multiple ways, and then looking at them to know which one looks better.”* (P6).

Some participants (P4, P13, P15, P18) noted that working in Jupyter Notebooks [36] was beneficial for quickly iterating over the design and exploring customizations due to its flexible integration in the workflow: *“To have it [the visualization] pop up in line and then in that way, I can generate it more easily, instead of having to go into the [data manipulation tool], generate a new file and then in the visualization tool, see how it works, go tweak again…”* (P15).

One participant pointed out that the design process is especially highly iterative when the visualization is supposed to be shared in a publication, and that this process could be demotivating: *“There’s like a very special graveyard where all of the really nice figures that never made into a paper […]. [Sometimes] I have to entirely stop an idea, [then] I kind of give myself like a few hours to grumpy and sad about it… And [after] I’m like, okay, let’s make a new one.”* (P4).

##### Limitations of Tools.

Challenges in the authoring process were often caused by tools lacking support on different criteria. Many participants illustrated challenges with the genome browsers (GUI+template). Often mentioned was the lack of integration in their workflow (P1, P2, P3, P4, P5, P12, P16, P20), requiring *“manually pasting data*”, or limited expressiveness to tailor visualizations to their needs (P1, P3, P5, P7, P9, P15, P18), e.g., *“cannot adjust or tweak”*,*“limited with colors”*,*“no multiple view options”*. Participants also found them to be lacking in efficiency (P5, P10), usability (P5, P8, P9, P17), ill-supporting the effectiveness of visualization *“cannot handle many samples”*, *“cannot sort”*, (P5, P7, P10, P17), and intuitiveness or learnability (P5, P10, P15, P17, P20). For example, P5 said: *“Using IGV has cost me a significant amount of time to navigate back and forth for different reference genomes.”*. Also, P20 stated *“It [the genome browser’s design] is not done in a very beginner-friendly way. It’s simple, but not intuitive.”*

Regarding code editors, learnability was often found to be an issue in the authoring process. We see a distinction for the types of code editors experienced by different personas. Authoring with low-level visualization libraries (e.g., Gosling [40]) often had a steep learning curve and was sometimes found to be impossible after initial attempts for “Bioinformaticians” and some “Computational Biologists” (P5, P9, P8, P6, P13, P19). They found this unfortunate because they saw potential in libraries, such as Gosling [40], to produce *“desirable”* or *“pleasant visualizations”*. *“It’s doing nice visualizations, but it took me some time to grasp.”* P9 said. *“It’s beautiful. It’s just a little bit hard to get your hands on JavaScript.”* P5 shared. *“I just need to put more time into it [Gosling], but … It felt a little harder than it should have been to get the views to link up.”* (P6).

Other “Computational Biologists” (P4, P15) illustrated learnability issues for some higher-level code libraries, such as ggplot [79], e.g., *“I’m not very good at it yet.”* (P4). Different from learnability, one participant raised the issue of limited creativity support when authoring visualizations in the code editor: *“I know that you can change a lot of these things in Seaborn, but sometimes I feel that figuring out all the specific things gets in the way of me exploring aesthetically.”* (P13).

##### Finding Tools and Trade-offs in Implementation.

Some participants also explained that finding and exploring tools was not always straightforward because of time constraints (P6, P19). For some participants, it was hard to assess whether tools would match their mental image of *“what I thought I could do with it”* or live up to their expectations in expressiveness for customizations (P8, P18), indicating obstacles in the gulfs of execution and evaluation [61]. *“[…] and then you want to add something and the software does not support that feature. That’s really annoying because then you have to find a different tool.”* (P18).

In choosing between off-the-shelf applications or code editors, a participant mentioned having an *“upfront cost of coding”* is better and further elaborated: *“It’s always useful to apply them [tools] when you can for exploration and when you do simpler things. But one thing about using the tools: the tools that I’ve used aren’t as implementable in a pipeline. That’s just another nice part of making it yourself.”* (P19).

#### 3.4.5 Characteristics of the Created Visualizations

Participants discussed authoring diverse visualizations that depended on the goals they had for analysis. Since the interview was centered on the construction of genome-mapped data visualizations, we mostly encountered one-dimensional linear *(20/20)* and circular sequence visualizations *(6/20)*, comparative two-dimensional sequence visualizations such as matrices or multiple sequence alignments *(5/20)*, and some highly customized glyph creations, e.g., for showing gene bodies or digital karyotypes, *(2/20)*. Although not the focus of our study, some participants also explained how they related these visualizations to non-genome-mapped data visualizations such as networks, trees, and tables (P6, P8, P14, P13, P15), often involving trying to author interactive linking between the views.

##### Static versus Interactive.

Nearly all participants stated that while they found interactivity useful, it was challenging to author and difficult to customize if they managed to incorporate it. The bottleneck mostly stemmed from a lack of skill and time to learn: *“I’m not going to kill myself to make it work. [… ] [I ask myself:] If it is half an hour to do using the tutorial, can I manage to get something interactive? If I’m like, no, this is too challenging, I’m not going to make a detour.”* (P15).

Only a few participants, mainly “Bioinformaticians” and “Software Engineers”, authored interactive views. They mostly authored tooltips or some other form of linking between visualizations and tables. However, they tended to encounter issues with authoring the linking methods (P6, P13). When referring to multiple views, many participants had little to no linking between them, displaying views one-by-one or in different tabs, but not side-by-side. *“[the views are] independent, but you know it would be interesting to have them linked.”* (P9). Interestingly, some participants (P8, P16) also reflected that they did not see the immediate value of having linked multiple views.

Furthermore, sharing of interactive formats rarely happened, even though it was thought of as potentially efficient and valuable for collaboration purposes. Participants also pointed out the challenge with interactivity for large data (P7, P18), experiencing it inefficient, *“hacky”* and not trustworthy: *“…Especially if you can rely on it [interactivity]. But it is very important if you present something, you are able to use it in real-time, so you have to make it work. If people have to wait 5 min, then it is a crappy experience. They will not like it.”* (P18).

Static visualizations, which were most often authored for communication purposes, were also found challenging to author with effectiveness and without overcrowding the view (P11, P13). For example: *“This is additional information that I feel is very hard to visualize easily. If we don’t represent it interactively, probably we will need to spend much more time understanding how the splicing looks like.”* (P11).

#### 3.4.6 Collaboration and Organizational Dynamics

Collaboration in authoring was mainly described as engaging with others for data questions, feedback on the design, or help with coding. In some cases, collaboration also occurred at the beginning of the creation process in the form of brainstorming. Some participants referred to situations where they used co-workers’ code pieces to build further and also with later involvement of them for feedback. However, the act of creating visual structures in authoring was described as an individual process by most. For example, P5 said *“It is mostly me who makes the visualization, and the developer [runs] the assembly tool.”*

Participants described drawing and getting inspiration advice from colleagues’ work, highlighting the influence of interpersonal relationships and the immediate work environment on the authoring process. *“I think [this visualization] was inspired by the work of [name], someone else in the lab.”* (P15).

Participants (P2, P4, P12, P14, P18, P19) sometimes described challenges related to sociocultural factors in their work environment. In most company settings, there is a tendency to prioritize familiar and *“not perfect but decent”* systems because there is no time to experiment or improve, e.g., *“it is a risk”* (P18) and *“need to convince my boss”* (P19). One participant in a company also felt that constructive feedback from coworkers was simply lacking: *“They’re very tactful to us. So, sometimes I feel like they would not request and say things and they would just accept what we are giving them.”* (P14). Some participants (P5, P12) in academic labs reflected that they encountered resistance to new tools in general: *“I feel like there is some inertia in the scientific community to switch to a different tool as widely used as IGV and genome browsers.”* (P5). In some cases, peers questioned the effectiveness of novel approaches, or there were language gaps between disciplines, therefore being barriers to trying new things. *“My PI doesn’t like [Circos visualizations], he called them circles of hell because he’s a clinician […]”* P9 said. Furthermore, P9 shared that there could be problems with jargon and abstractions: *“[the documentation for these plots] is more from a visualization point of view, not from a genomic point of view. [It could be] more compiled, [e.g. specifying whether it’s] at genome level or chromosome level; from the [visualization] output.”*

#### 3.4.7 Reflections on Ideal Workflows

##### Example-based and Natural Language Techniques.

We extracted several potential methods for improving the authoring steps of participants based on their reflections on the ideal workflow. Working with examples was often mentioned as a strategy or desired way to start with authoring or when they needed inspiration for an idea *(12/20)*. An example-based authoring technique that takes images, code, or sketches and lets users copy the aesthetics or layout configurations might be a useful authoring technique to provide to users. Natural language was also referred to by participants *(5/20)* as a potential solution for implementation and ideation challenges, either in mixed-initiative [30] (P18) or post-editing [26] (P3) settings.

##### Interactions and Automation.

Participants found interactions cumbersome to author or not beneficial for their analysis goals. Related to the lack of interactivity, participants experienced a lack of automation support and, therefore, needed to manually handle certain authoring tasks, especially customization. For example, participants reflected on the usefulness of having powerful *“all-in-one”* (P2) or GUI systems (P16); having support for *“logical”* data manipulation similar to Pandas, for example (P15); additional views with summary statistics (P9, P11, P17, P20); semantic zooming between views for intuitive navigation (P6, P10, P18); or direct manipulation options (P8) and recommendations to quickly explore, customize, and interactively annotate visualizations in the same environment (P14).

## 4 ELICITING VISUALIZATION AUTHORING INTENTS (STUDY 2)

After gaining clarity on the limitations in the current authoring practices of genomics experts, we followed up with an exploratory study to better understand how genomics experts ideally want to author visualizations. We conducted an elicitation study using visual design probes, inspired by Crisan et al. [19], who used probes for learning about users’ intents and design choices for interactive machine learning.

### 4.1 Visual Design Probes

Using visual design probes, our second study explored which types of authoring techniques were considered helpful by genomics experts and in which tasks. The authoring techniques considered in the current situation of genomics authoring are limited compared to the state-of-the-art described in visualization literature [24,58]. If genomics experts were offered more options for handling the authoring tasks, would they also use those, and why? By studying this, we aimed to learn more about the authoring needs of genomics experts and whether there were any notable differences among the five newly identified personas.

We designed visual probes to explore user preferences for visualization authoring techniques in each domain task for genomics data visualization. These probes included visual and interactive aids to help get started and guided participants through realistic scenarios, prompting reflection and interaction with authoring techniques. For participants, the probes served as a canvas to express their approach. At the same time, the probes provided us a means to collect quantitative and qualitative data observing and analyzing participant interactions.

**Fig. 2:**
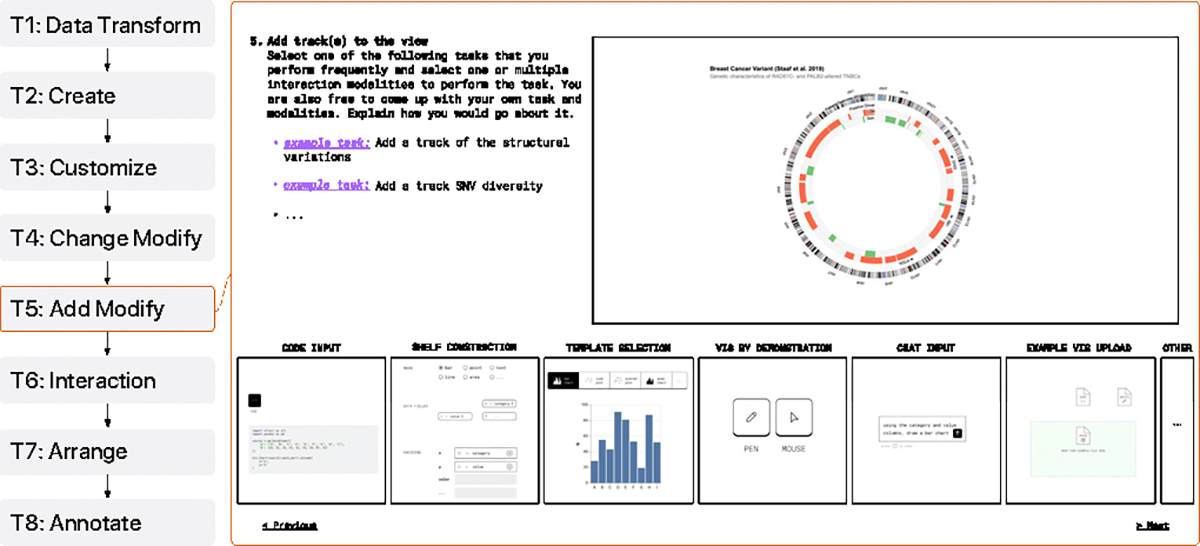
Example circular condition probe for modification task (T5), showing prompts with example tasks (top) and technique thumbnails (bottom).

We created two sets of visual design probes that guided the users through different authoring scenarios. Each set provided the user with example data and visual encodings in linear or circular layouts, as these are common to view genome-mapped data. An example probe is shown in Fig. 2. In the top-left of each probe, textual instructions and example domain tasks were provided. However, participants were also encouraged to come up with their own that better reflect their everyday tasks. At the bottom of each probe, we displayed six techniques—code editing, NLI, shelf configuration, template-based, VbD, and example-based—to choose from as a gallery of thumbnails (randomized order per participant). This gallery included an extra option at the end to encourage participants to propose techniques that do not correspond to any of the six provided techniques.

#### Design Considerations.

We designed the probes with the following set of design considerations:

- *Coverage:* We created probes covering a wide range of authoring tasks identified through the literature review and informed by the Study 1 interviews. The clarity and vocabulary correspondence of the task prompts were tested with two pilot sessions.
- *Granularity:* To gain nuanced information about different large editing subtasks, we split the **modification** task (Sec. 2.1) over two probes: one for visualization type changes and another for adding encodings, making a total of eight probes per participant.
- *Relevance:* We provided several domain task options for each probe that participants could choose from. This allowed participants to reflect on situations close to their actual work scenario while safeguarding some structured insights for the study.

The probes were implemented using Figma [22]. From the pilot study, we learned that Figma was a more expressive medium for our goals than Google Slides (used in prior work [19]). This expressivity was especially deemed important for the onboarding session, which guided the users through interactive demos of the thumbnails in the main probes. This enabled participants to experience individual authoring techniques in an engaged manner so that they could provide more meaningful feedback. Using Figma’s interactive components and prototyping features enabled us to include minimal interactions needed in the visual probes, such as drag-and-drop for shelf configuration and a prompt for NLI. After learning about the affordances of the techniques, users are reminded of those in the probes by the thumbnails, which in itself can only be selected or enlarged. We deliberately kept the interactions in the probes limited to avoid attribution biases. For the same reason, we used sketchy UI components and icons.

We used Gosling [40] to create visualization images to be shown on the visual probes. We tried to use datasets [6,16,27] that are familiar enough to participants by referring to their visualization examples that were submitted from Study 1. These included Pan-Cancer Analysis of Whole Genomes (PCAWG) structural variation [1], ChIP-seq samples [41], and GRCh38 genes [27].

### 4.2 Participants and Procedure

The participants were invited from the pool of participants in the first study. The 13 participants are marked in column “S2” in Table 1 and represent all user personas apart from “Visualization Experts”. Participants who completed the second study received another $25 Amazon gift card for a 1-hour remote interview session using Zoom. We followed a three-step procedure for this study:

1. *Pre-test Training (15 min):* To familiarize the participants with the six authoring techniques, we created a pre-test probe for each technique that embedded a video of an example tool with the respective technique. Participants could then try it out for themselves in the Figma prototype. After the training, they filled out a Google form to test whether they understood each technique.
2. *Interaction with Probes (20 min)*: After clarifying any confusion about the techniques, participants interacted with the probes and were encouraged to think out loud [55] to clarify their intents. Each participant received probes for genome-mapped visualizations in either circular or linear layout based on insights from Study 1. We asked participants to envision themselves working with an authoring system where all these techniques are available to use and combine. We reminded participants that the techniques were not actually functional in the probes, but that the thumbnails could be used to explain and reflect on their intent.
3. *Post-test Questionnaire and Short interview (15 min):* After the probes, we asked the participants to fill out a short survey to measure their familiarity with the techniques and perceived usefulness. We concluded with a few open questions about surprises and reflections from the interactions with probes.

### 4.3 Results

**Fig. 3:**
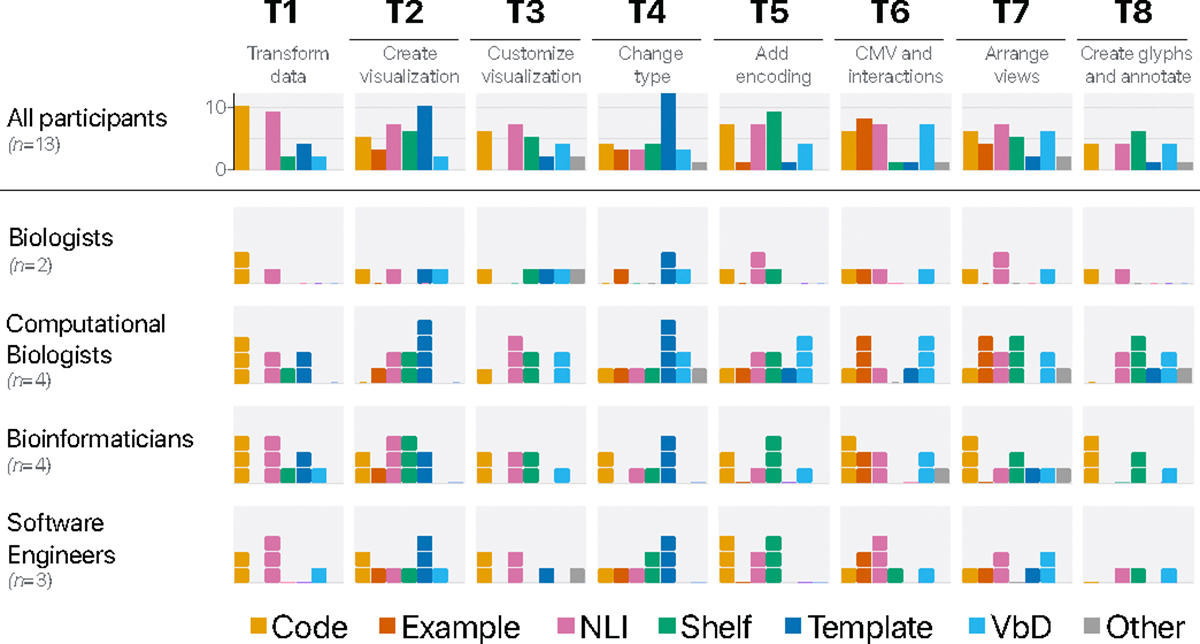
The frequency of participants’ responses (multiple possible) over visualization authoring techniques (Code, Example, NLI, Shelf, Template, VbD, and Other) for given tasks (T1–8) and personas (“Biologists”, “Computational Biologists”, “Bioinformaticians”, and “Software Engineers”).

We performed thematic analysis and used descriptive statistics on notes and coded interactions from video-recorded sessions to analyze the results. In Figure 3 we summarized high-level patterns for the interactions with the probes. We describe the patterns below with participant quotes for context. Note that participant identifiers in Study 2 summarized below do not correspond to participant identifiers in Study 1.

#### 4.3.1 Authoring Techniques by Tasks and Personas

##### Data Transformation [T1].

Code editing and NLI techniques were both most often chosen to handle the data preparation task. Participants elaborated that code would be *“more explicit”* and feel like having *“more agency over manipulating the data”* (P12, P13). Natural language was found *“easy to express”* (P7), where example prompt from the participant was *“Keep all reads with the mapping quality over 30”*. Participants also saw a potential for use of templates. Although this may not seem like an obvious choice, it is not surprising. Genome browsers such as IGV [72] already offer functionalities for automatically deriving common data, such as the coverage score. For example, P10 thought the functionality was *“standard in genome browsers”* or *“tedious to code”*. Interestingly, one participant thought about a combination of NLI and code: *“asking the chat to do the code and then continue with code on my own”* (P7). For this task, we do not observe any patterns that distinguish the choices of particular personas, other than templates mostly being chosen by “Computational Biologists” and “Bioinformaticians”, possibly due to their familiarity with genome browsers.

##### Specification [T2].

Template-based editing was most often picked for this task, followed by NLI, shelf configuration, and code editing. Templates were referred to as *“easy”* for this task, but also NLI was thought of as being *“*easiest *and [requiring] less effort”* (P6, P11). Shelf configuration was also intuitive and *“faster”* for some, while others had to think a bit more about the encoding: *“I have to think about what my x-axis is, what my y-axis is, …”* (P12). P5 mentioned that uploading an example sketch could be *“pretty natural. […] I normally draw what I want the figure to look like.”* Furthermore, we observed that shelf editing was never expressed by “Biologists”.

##### Customizations [T3].

For making customizations, such as a change of color encoding, NLI was most often referred to as the sensible choice.

For example, the specifications would be *“very straightforward to translate”* or *“cosmetic changes seem to be suitable for code inputs”* (P6, P8). Code was preferred because of the support for fine-grained control, e.g., by tweaking *“the exact hex color code”* (P10). One participant reflected on using NLI+code, and thought code editing would be suitable for *“adjustments”* after the rough specification. Participants also thought about using VbD, for example, to change the bin size of the bar chart by *“dragging the interval”* or *“drawing [splitting] one bar in half”* (P8, P13). Interestingly, one participant thought about using shelves as a way to learn how to achieve it in code: *“teaching myself to use shelf construction and then export the code to understand the code”* (P11). Some participants thought of strategies not provided in the probe, such as using a WIMP-like interaction, *“right mouse click*” to change the color, or picking from a color palette as typically supported in presentation software (P13). While using NLI, code editing, and VbD were mentioned by different types of users, mainly users fitting the “Biologists”, “Computational Biologists” and “Bioinformaticians” persona reflected on authoring with shelves.

##### Modifications [T4, T5].

For modifying the visualization type, almost all participants immediately resorted to templates *(12/13)*. They motivated that *“if [the visualization] is basic, then”* this would be good and *“being able to test a lot of things” “quickly”* (P5, P11). One said it could potentially be because of *“bias to software already used”* (P10).

Example-based authoring was mentioned by one participant in case a *“very very specific style”* of visualization would be needed (P6). VbD was highlighted as a good option too: *“tracing the top of the plot”* to transform a bar into a line chart or *“If so complex … draw rather than describing it (in textual representations)”* (P8, P9).

Another modification task was to add a summary track (i.e., a metric computed over additional data not yet encoded) to the existing view. Because novel information needs to be added, this led many participants to think of shelves as a strategy to achieve it *(9/13)*. Also, code editing and NLI were frequently discussed, for example, in the case that the novel track involved calculating over the attribute(s) *“[prompt:] within this region, what’s the count of … and display it”* or *“if you want a certain number of things…”* (P9, P11). NLI was discussed as a way to have a *“co-pilot”* (P7). Using VbD with pen input to draw the additional track was found plausible too for circular layouts *“drawing lines inside the inner track”* (P13), as well as using example-based strategies because that would be useful for *“detailed output, or something hard to implement”* (P6). However, one participant remarked that using an example felt like *“an extra step”*. This adding track task also sparked ideas for combinations of techniques, such as NLI+VbD *“using chat input and then draw a line to let the system know where”* (P8), example+code *“to adjust”* (P4), or example+NLI (P3). Regarding personas, VbD strategies seemed to be primarily thought of by “Computational Biologists”; all others were mixed between personas.

##### Coordinated Multiple Views and Interactions [T6].

As highlighted in Study 1, interactivity was not necessarily common practice for the participants. When given the task to duplicate or link a (whole genome) view with a brush to the detailed view, many participants thought of using examples with *“screenshots”* or *“sketches as the starting point”* to create this (P3, P8). NLI was also frequently helpful to get started: *“I don’t see how I should do that … so I will probably use [NLI]”* (P4). One participant commented: *“I’ve never seen something like it before”* (P7). One participant reflected on their current trials to make it work in their work: *“I had a lot of trouble linking views …”* (P10). Interestingly, participants found that VbD could work by *“drawing the link between views”*, *“selecting a region [to become the brush]”*, *“click and zoom, then duplicate”* or *“like taking screenshots in MacOS: select a subsection, and then click on the ‘create a new view’ and zoom into this section”* (P5, P8, P1, P11). One participant further reflected that VbD could be combined with shelves *“and the use shelf configuration to remove tracks of not interest”* (P11). Another technique that did not fit the ones offered in the probe was to *“drag and drop [shapes]”* to create the brush (P12), indicating a visual builder [24] technique.

##### View Arrangement [T7].

Code and NLI were often discussed to change the arrangement of multiple views. Also, DM (labeled as “Other”) strategies were mentioned, making analogies to presentation software, e.g., *“Drag the plots to a certain area with a mouse, and ‘snap”‘* (P2, P3, P6, P11). One participant (P9) considered drawing an arrow to show the direction of the ordering views using VbD. Similar to some previous tasks, code would be a good candidate to make it precise and *“determine the size of the grid”* (P10). Shelves were also mentioned, but we suspect participants confused it with what would be more of a template editing technique, for example, *“dragging the data to a specific cell in a table template (or zones)?”* (P5).

##### Glyph Creation and Annotation [T8].

The last task was to create and add a custom genome annotation glyph, deliberately framed as an open task to invite people to think of a scenario applicable to their research. Here a variety of approaches was described based on how customized the glyph would need to be. For example, shelves were used by participants to *“provide additional data [encodings]”* (P8) and code to *“have an exact way of defining [(genomics) coordinates]”* (P10). VbD was for basic annotations such as *“Adding a label”* and *“writing comments for sharing”*. One participant (P2) mentioned a combination of 3 techniques to perform the task, using example+NLI+VbD, with examples to *“start in a certain style or with figure from another paper”*, and to then *“adjust that style with NLI and VbD”* to arrange labels.

#### 4.3.2 Summarizing Elicitations

None of the participants stuck to a single authoring technique across all tasks and noted that different authoring techniques accomplished different tasks. Participants reflected on the value of having flexibility in the system to be able to create expressive visualizations.

##### Natural Language Not Always Natural?

Code editing was often the technique of choice for tasks requiring fine-grained specifications. Moreover, code solutions were generally considered when participants knew there was a way to do it. In situations where it was harder to know how to author the visualization, but the task was specific, NLI was often considered. Furthermore, NLI could help to *“specify all in one”* mappings (i.e., visualization type and coordinates) so that the author would not need to worry about how to communicate low-level encodings. However, the reverse was also found true. With NLI, interactions did not seem *“natural”* to explain in language for complex tasks, such as visualizing BAM tracks. Furthermore, there was skepticism towards NLI due to the potential lack of accuracy in interpreting the authoring intent—then needing to revisit those authoring actions—and concerns about privacy and data handling.

##### Communicating Visually for Visualizations.

Participants were most unfamiliar with VbD and example-based techniques. However, communicating with visual cues for visualizations, using examples or demonstrating, was often used during the probes. In particular, VbD seemed useful for position-related customizations and modifications, e.g., to create linking and brushing interactions between views. However, the automated aspects of the techniques were also found to be vague; similar to NLI, there could be *“a gap between what you want to create vs. what you would get”* (i.e., gulfs of execution and evaluation [32]).

## 5 DESIGN IMPLICATIONS

We highlight several design implications and research opportunities, motivated by our findings presented in Sec. 3 and Sec. 4.

1. **Combine Techniques for Better Expressiveness and Learnability.** Our user studies show that multiple techniques are used for various tasks and by all personas. Tool builders should consider adding support for multiple authoring techniques to help genomics experts in the visualization authoring process. For some, having the choice of multiple authoring techniques can help to create expressive visualizations more easily. For others, it might offer an opportunity to learn more advanced, expressive techniques. Because of these multiple criteria, integrating authoring techniques into a system can be beneficial in both redundant and complementary ways [45].
2. **Integrating Workflows for Data Exploration and Presentation.** Participants often described and reflected on situations where poor integration between tools and frequent context switching hindered their overall workflows, as was also pointed out by L’Yi et al. [42]. We found that authors need to overcome significant discrepancies between interactive GUI data viewers and tools for designing publication-ready figures to use them together in their overall workflow. Better integration of these workflow phases can be achieved by supporting more flexibility [3] in how a visualization design can be authored.
3. **Guided Support in Creating Multiple Linked Views.** Our studies uncovered that authoring interactions, especially linking views, is a non-trivial task. Support for authoring interactions currently needs to be improved for genomics tools; it is only possible in a few lower-level coding libraries such as Gosling [40], primarily used by personas with more advanced visualization and programming skills (e.g.,”Bioinformaticians” and “Visualization Experts”). Future work should explore ways to guide the authoring process of interactions for genomics visualizations, such as by providing sensible defaults or automatically linking and brushing views with shared properties that authors can further customize.
4. **Data and Design Sensemaking in the Same Context.** Similar to integrating the process of data exploration and presentation, tools should help authors explore visual designs in the same context as where they do data analysis. We found that genomics authors typically cannot do this due to the multitude of tools used and the complex data flow between them. Often, there is a disconnect between where computations and data are processed (e.g., computational notebooks on servers) and where visualizations are viewed, resulting in disjointed workflow components and a complex process. Bridging the gap in this sensemaking process [74] could enable users to author more effective representations with greater efficiency.
5. **Support Collaborative Authoring.** Receiving feedback is an integral part of the authoring workflows of genomics experts. Furthermore, genomics visualization authors collaborate by exchanging pieces of code. Tool builders can explore different modes of facilitating this handoff [64] collaboration processes, for example, by providing features that show the “delta” between two versions of a visualization at different levels of detail [38].

## 6 DISCUSSION

Our interview studies, based on 33 interviews with 20 participants, are the most comprehensive investigation of how genomics researchers are using visualization in their work to date. The five personas and corresponding analysis contexts we characterized are related to yet differentiated from those identified by others [78], giving us confidence in our findings.

The results of these studies, in particular the design implications, will be valuable to researchers who are creating visualization tools for genomics researchers and those in adjacent scientific fields that rely on large-scale biological data (e.g., single-cell analysis data). There might also be lessons for researchers in other data-intensive fields or those with similar data complexities (e.g., time series analysis), where customized solutions for diverse users are needed. As we have shown, user needs are highly dependent on their analysis approaches, which in turn depend on the focus of their questions and the audiences for the visualizations that they author. Customized visualization authoring solutions, leveraging multiple techniques, are expected to be more effective for diverse users.

The representativeness of our study participants is a potential limitation of this work. Even though 20 participants is a large number for a study of this kind, we found that our participants are mostly working in computational settings. Only a few participants are primarily working in experimental or clinical settings. Another audience that we hoped to recruit but were unable to were those in supervisory roles (principal investigators, managers, etc.), who typically rely on others to author visualizations for them. More information about their approach would help clarify their role, e.g., in collaborative authoring. While our findings have potential value beyond biological domains, their relevance might differ across fields. In particular, the results of the second study might not be directly applicable to other user audiences, given the study’s reliance on visual probes, which were found to be less transferable than other approaches [66]. Further work is needed to validate the generalizability of our findings.

## 7 CONCLUSION

The investigation in this paper provides a comprehensive view of genomics researchers’ visualization authoring practices and a detailed characterization of the contexts in which visualization authoring takes place. These findings will inform the design of future genomics visualization tools and improve their effectiveness. In particular, the design implications identified here provide valuable guidance for visualization researchers and can motivate future work.

## Supplementary Material

tvcg-3456298-mm

## Figures and Tables

**Table 1:** The 20 user study participants assigned to newly identified personas for genomics data visualization authoring—*“Biologists”, “Computational Biologists”, “Bioinformaticians”, “Software Engineers”, and “Visualization Experts”*—with their demographics and skills in genomics (gen.), data processing (data), programming (prog.), and visualization (vis.) and mappings to Bioinformatics personas [78] and Data Science personas [18].