# Supplementary material for: Understanding Visualization Authoring Techniques for Genomics Data in the Context of Personas and Tasks
Source: IEEE Trans Vis Comput Graph. Author manuscript; Available in PMC 2025 Mar 4. (PMC11875953; doi:10.1109/TVCG.2024.3456298)
Supplement: tvcg-3456298-mm [file NIHMS2039885-supplement-tvcg-3456298-mm.zip › tvcg-3456298-mm/probe_example_linear_figma_export.pdf]

🤖 Interaction with 8 Probes

In the following 8 probes, you will be guided through parts of a visualization construction process.

For each probe, you may get partial data or visualization results at the start

- At the top of each probe, some instructions and example tasks are provided.
  - You can choose one task or come up with your own task.
- At the bottom, you will see some modality options. **You should envision that you are working within a system where you have these modalities at your disposal and they all work**. However, in these probes they are not really functional; you can only select or deselect modalities to show your intent.

Most importantly, for each probe:

- Try to **describe your thought process in detail** while you point things out on the probe. We might interrupt you and ask you follow up questions.
- You are encouraged to go beyond the examples and do things that would be closer to your own work. You can also think of other modalities not shown.
- On average, you can spend about 3 minutes per probe. We will give you a heads up about the time if needed.

Start

1. Data transformation

Select one of the following tasks that you perform frequently and select one or multiple interaction modalities to perform the task. You are also free to come up with your own task and modalities. Explain how you would go about it.

- *example task:* Calculate the coverage score for the given BAM file
- *example task:* Filter out low quality reads
- ...

BAM file

r001	163	ref	7	30	8M4I4M 1D3M	=	37	39	TTAGATAA AGAGGAT ACTG	*
r002	0	ref	9	30	1S2I6M1 P1I1P1I4 M2I	*	0	0	AAAAGATA AGGGATA AA	*
r003	0	ref	9	30	5H6M	*	0	0	AGCTAA	*
r004	0	ref	16	30	6M14N1I 5M	*	0	0	ATAGCTCT CAGC	*

Pileup visualization

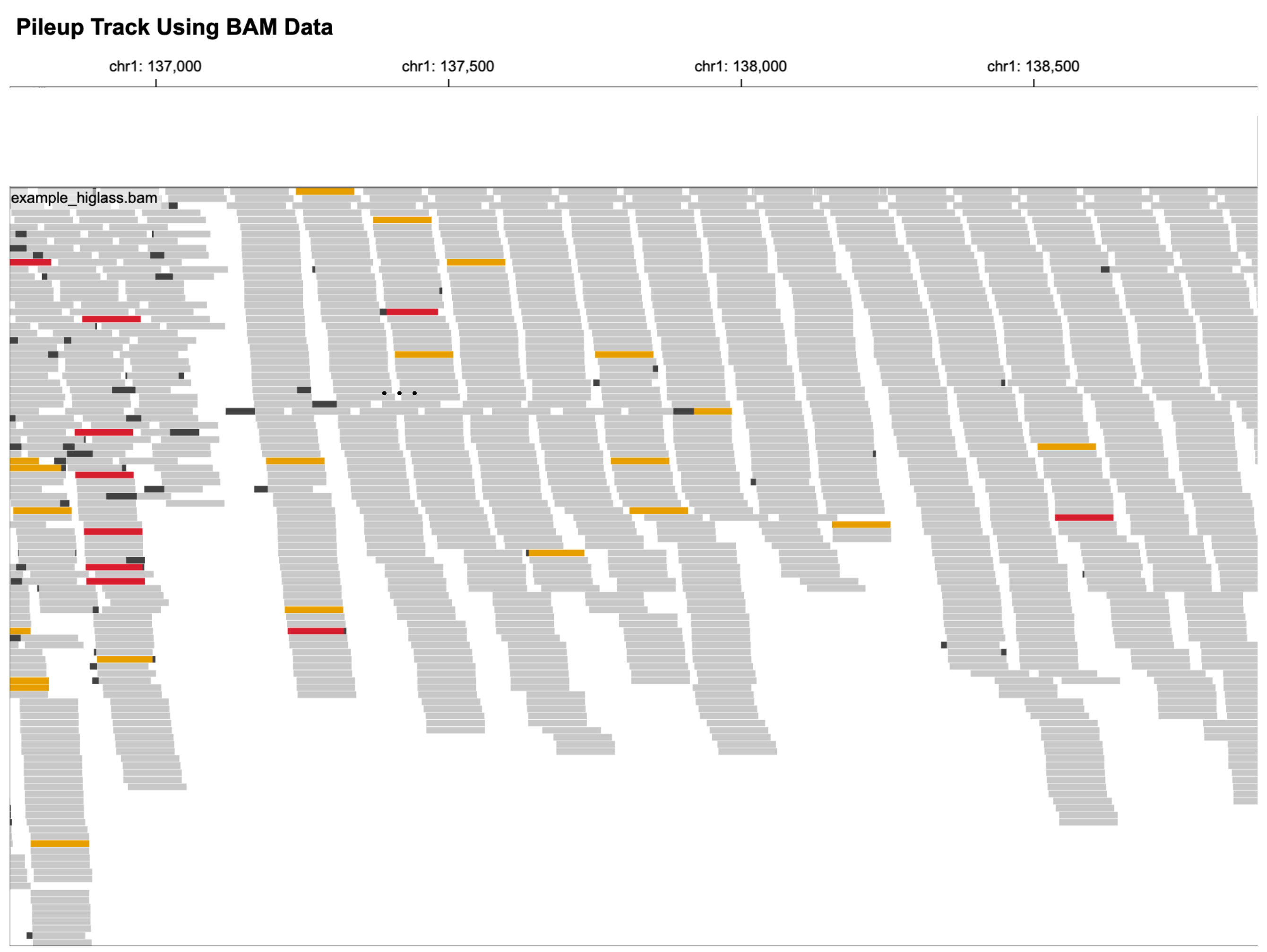

CHAT INPUT

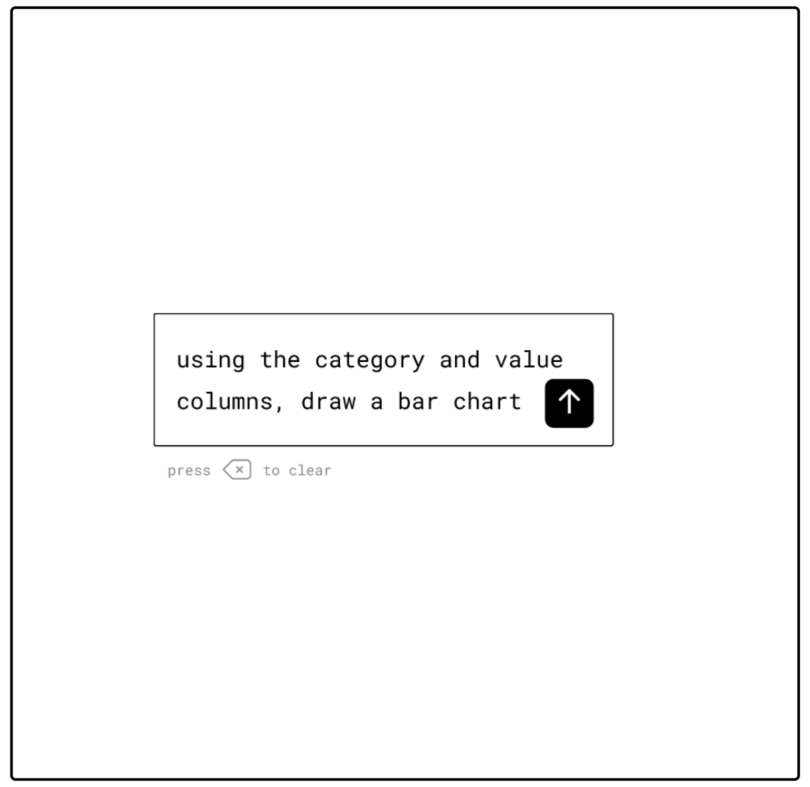

SHELF CONSTRUCTION

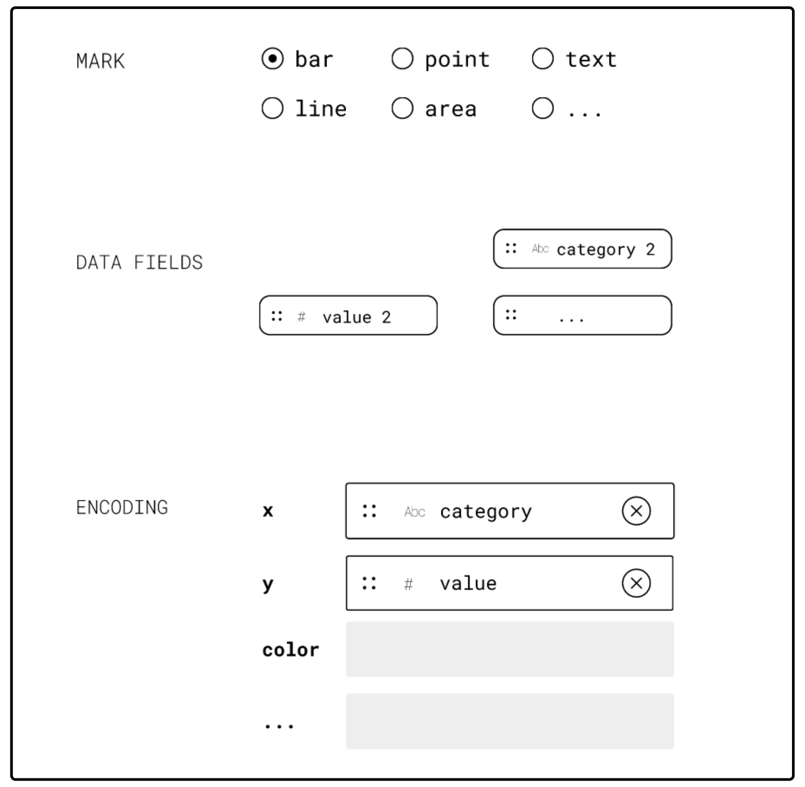

TEMPLATE SELECTION

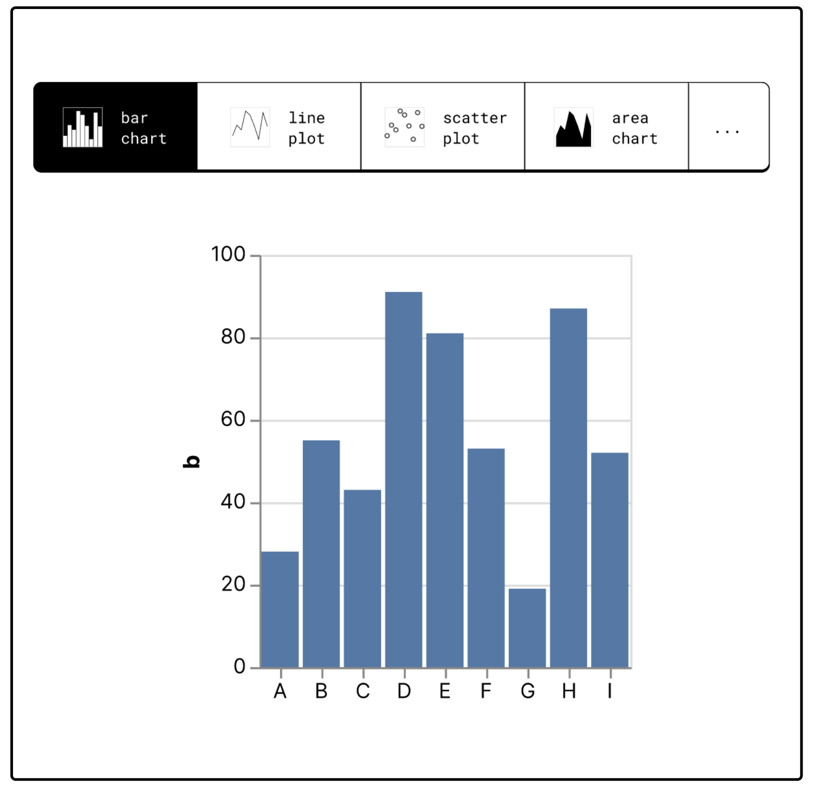

VISUALIZATION BY DEMONSTRATION

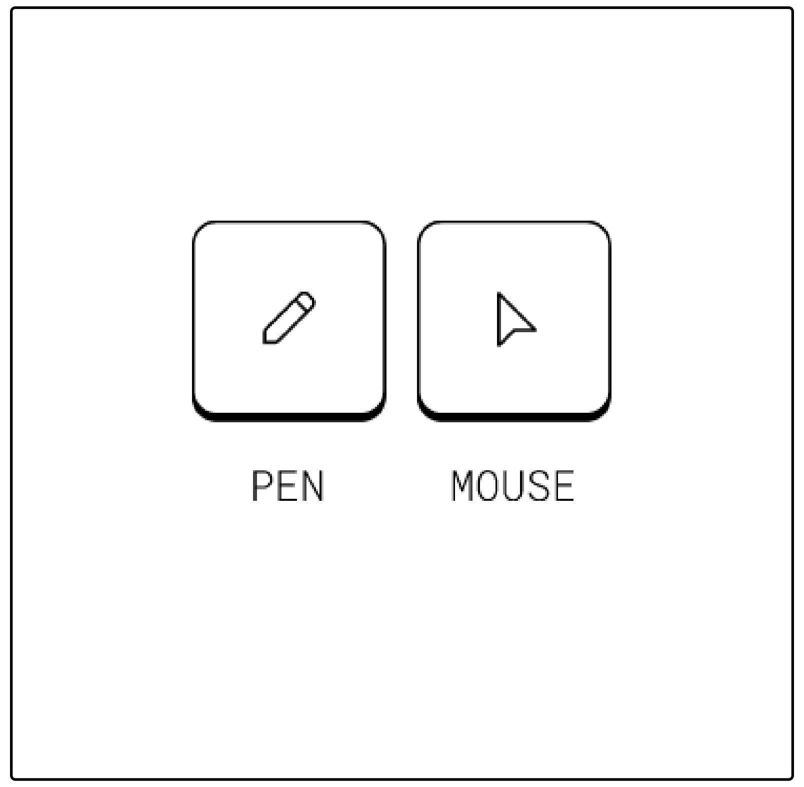

EXAMPLE VISUALIZATION UPLOAD

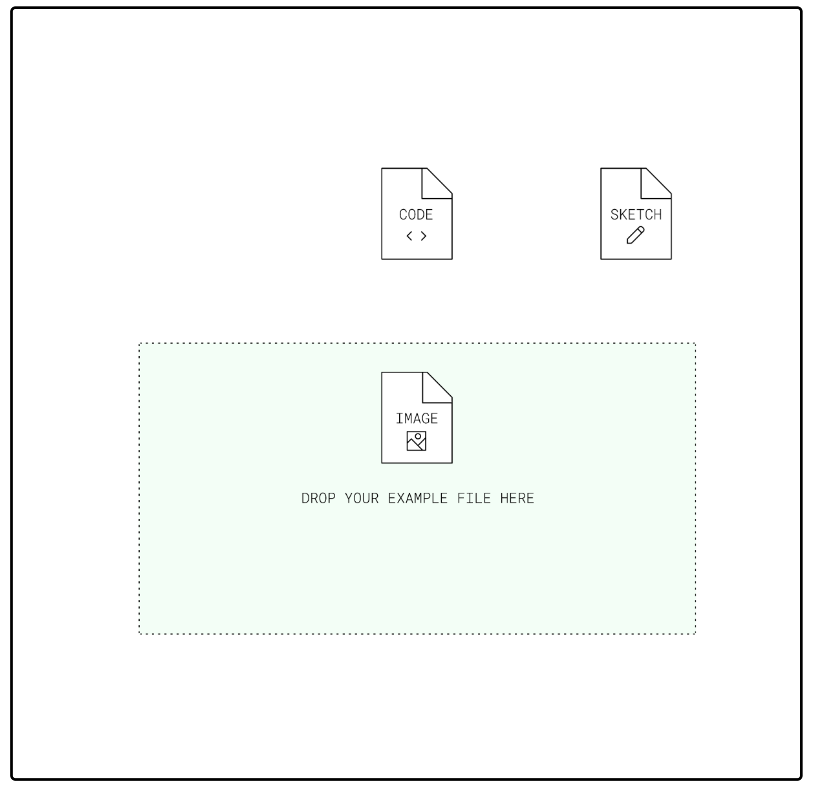

CODE INPUT

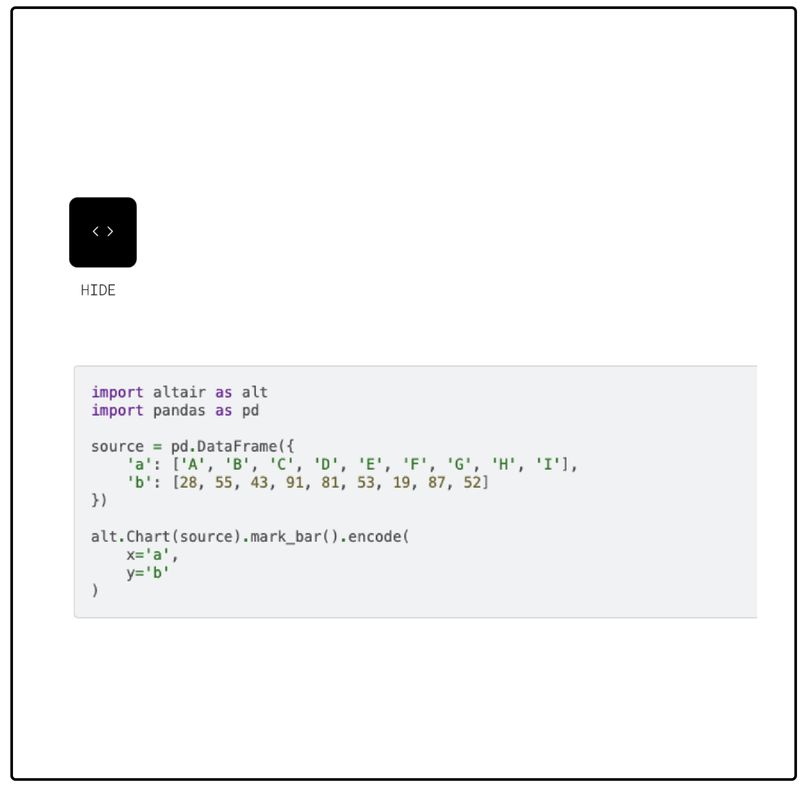


```python
import altair as alt
import pandas as pd

source = pd.DataFrame({
    'a': ['A', 'B', 'C', 'D', 'E', 'F', 'G', 'H', 'I'],
    'b': [28, 55, 43, 91, 81, 53, 19, 87, 52]
})

alt.Chart(source).mark_bar().encode(
    x='a',
    y='b'
)
```

OTHER

...

< Previous

> Next

2. Visualization creation

Select one of the following tasks that you perform frequently and select one or multiple interaction modalities to perform the task. You are also free to come up with your own task and modalities. Explain how you would go about it.

Create track that visualizes:

- *example task:* a bar chart of the coverage score (BAM file)
- *example task:* a line chart of a BigWig file
- ...

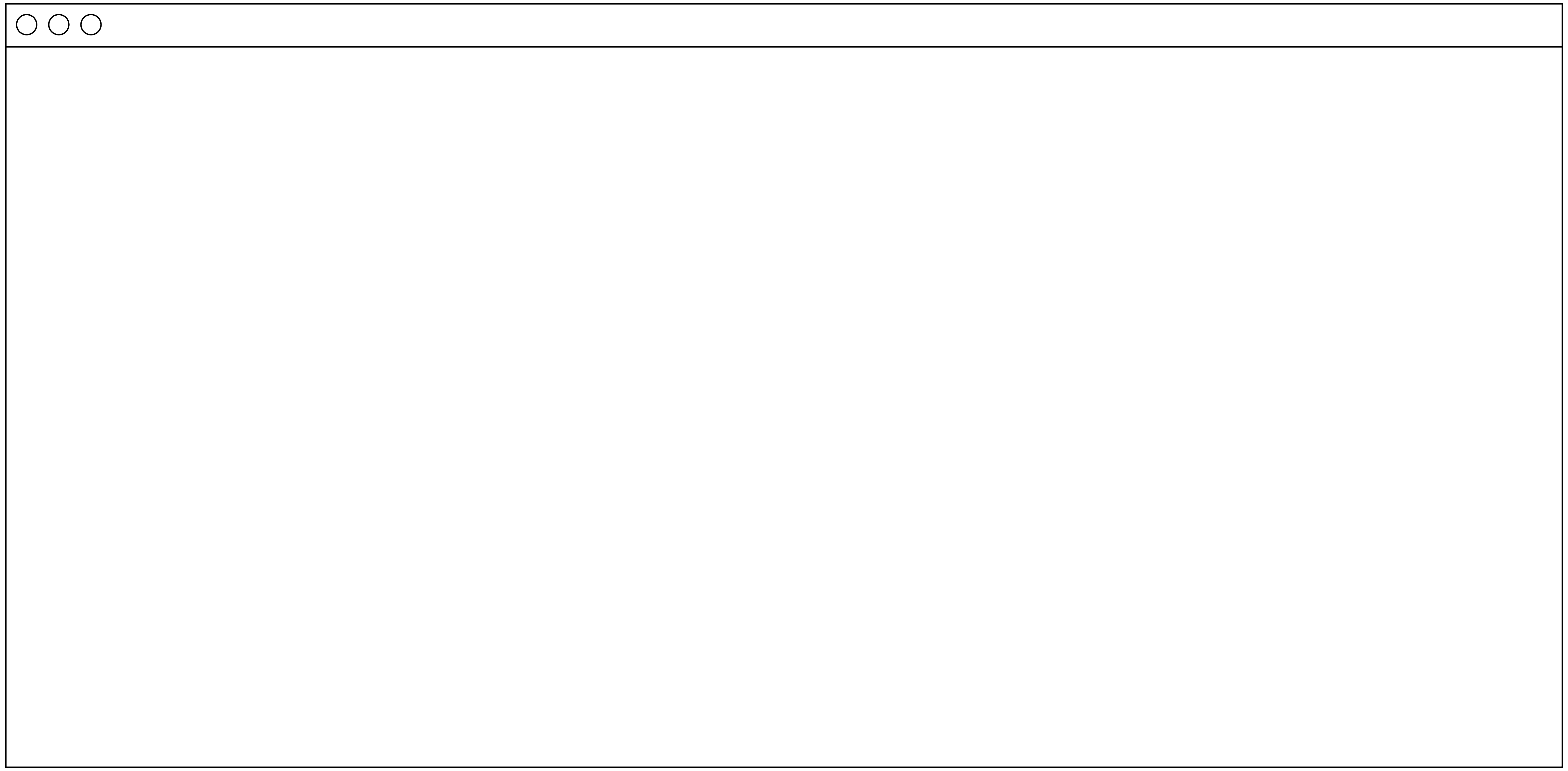

CHAT INPUT

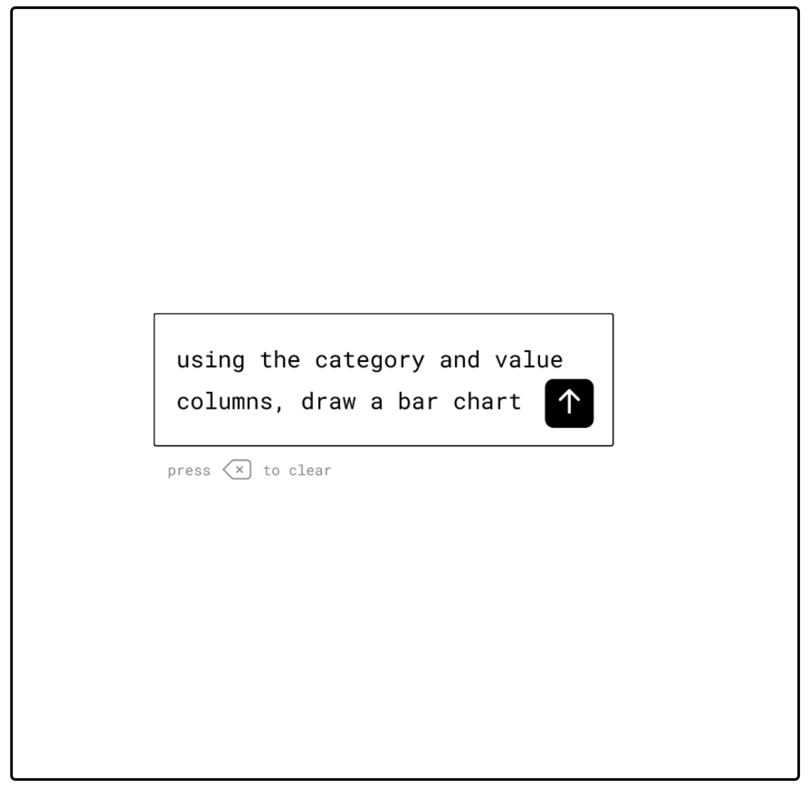

SHELF CONSTRUCTION

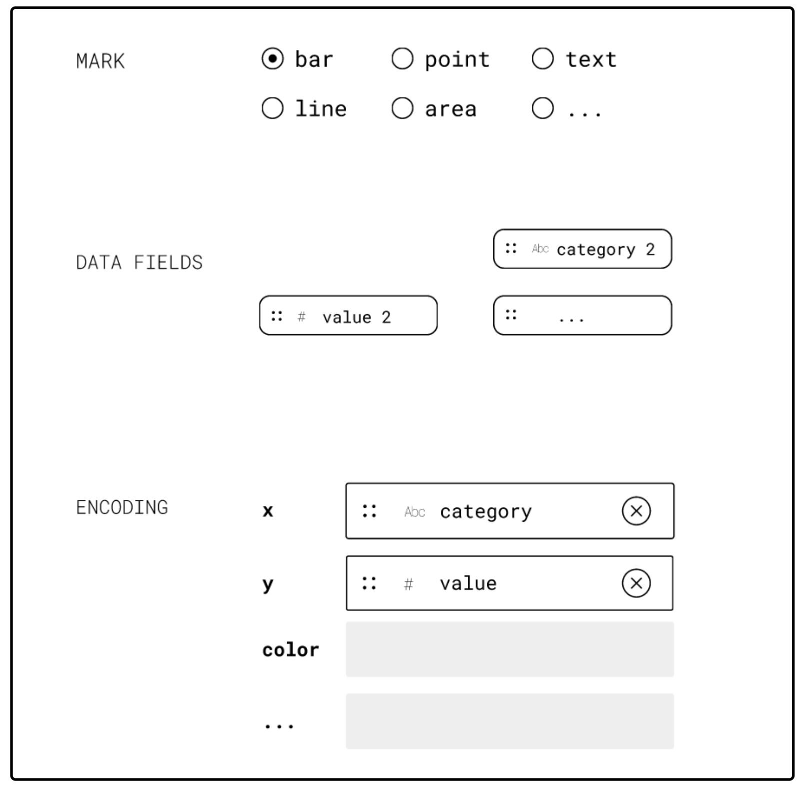

TEMPLATE SELECTION

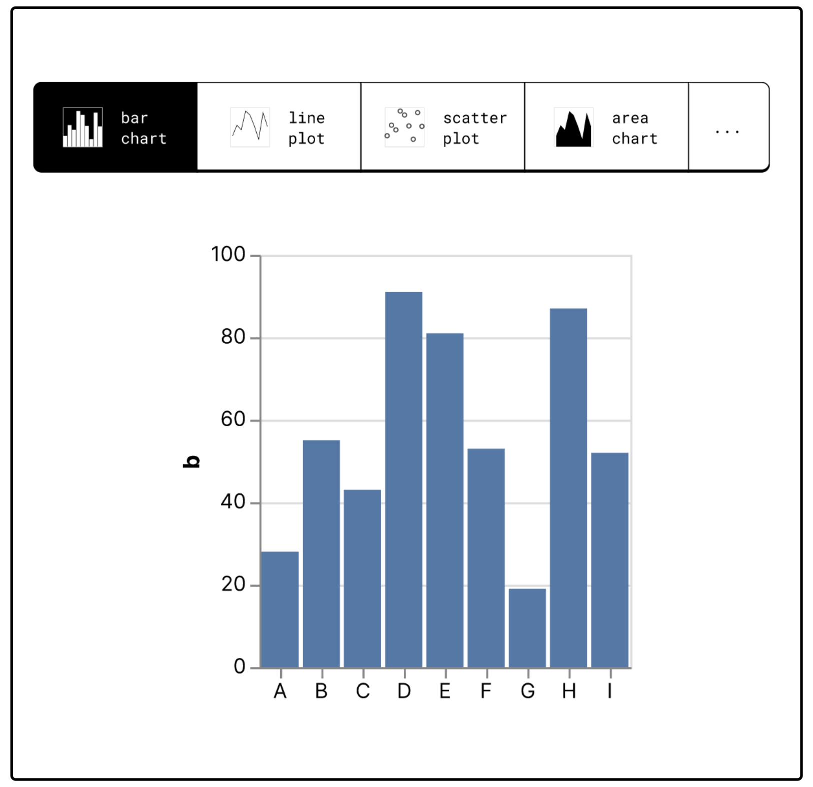

VISUALIZATION BY DEMONSTRATION

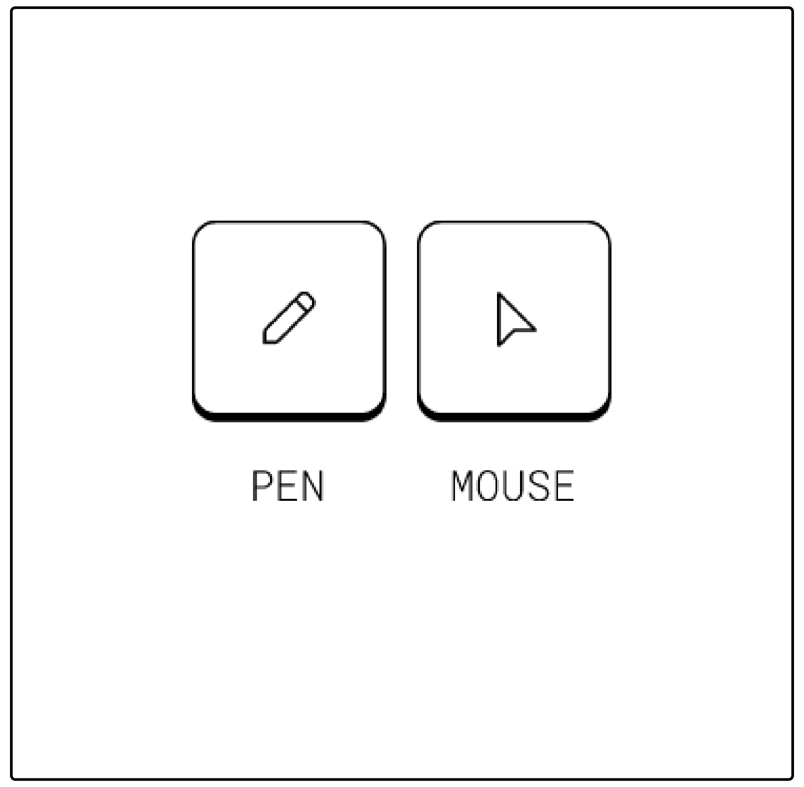

EXAMPLE VISUALIZATION UPLOAD

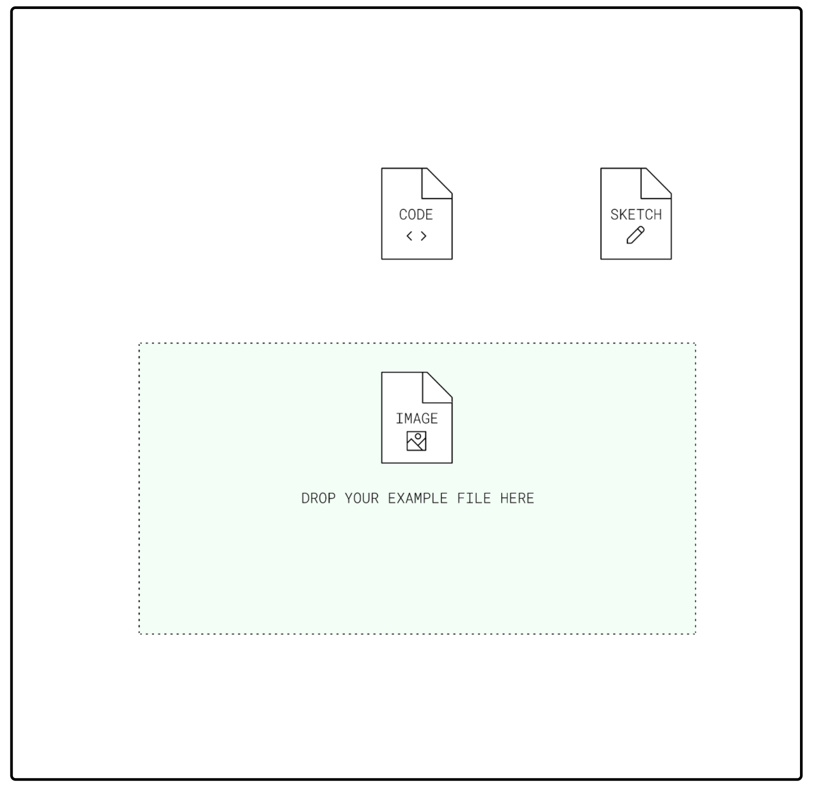

CODE INPUT

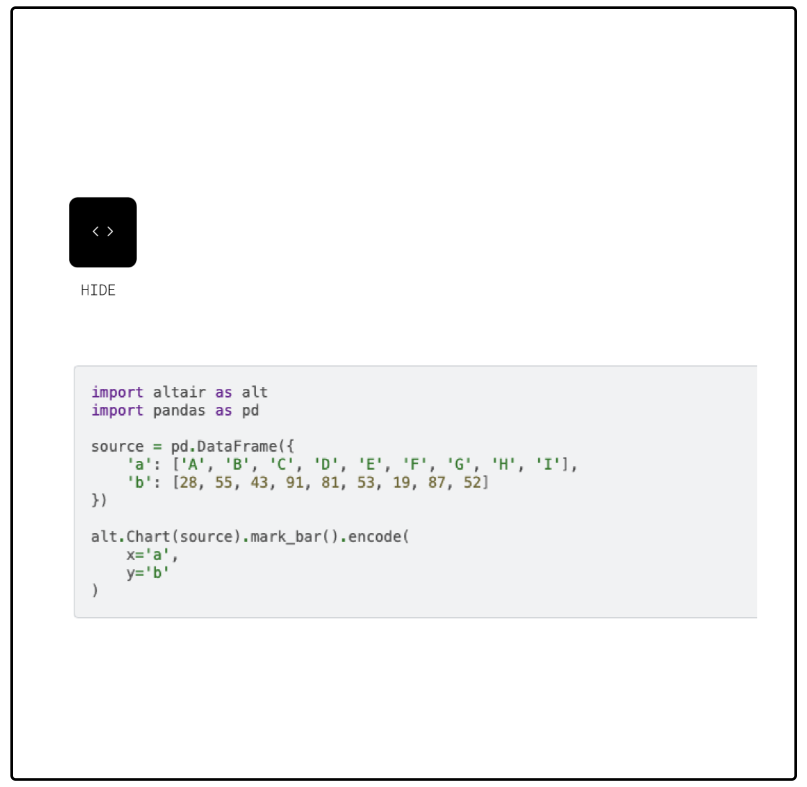

OTHER

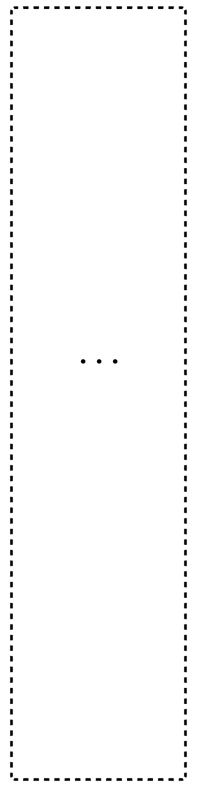

< Previous

> Next

3. Visualization customization

Select one of the following tasks that you perform frequently and select one or multiple interaction modalities to perform the task. You are also free to come up with your own task and modalities. Explain how you would go about it.

Customize the visualized track

- *example task:* Change the color coding of the visualized track
- *example task:* Change the x-tick interval of the visualized track
- ...

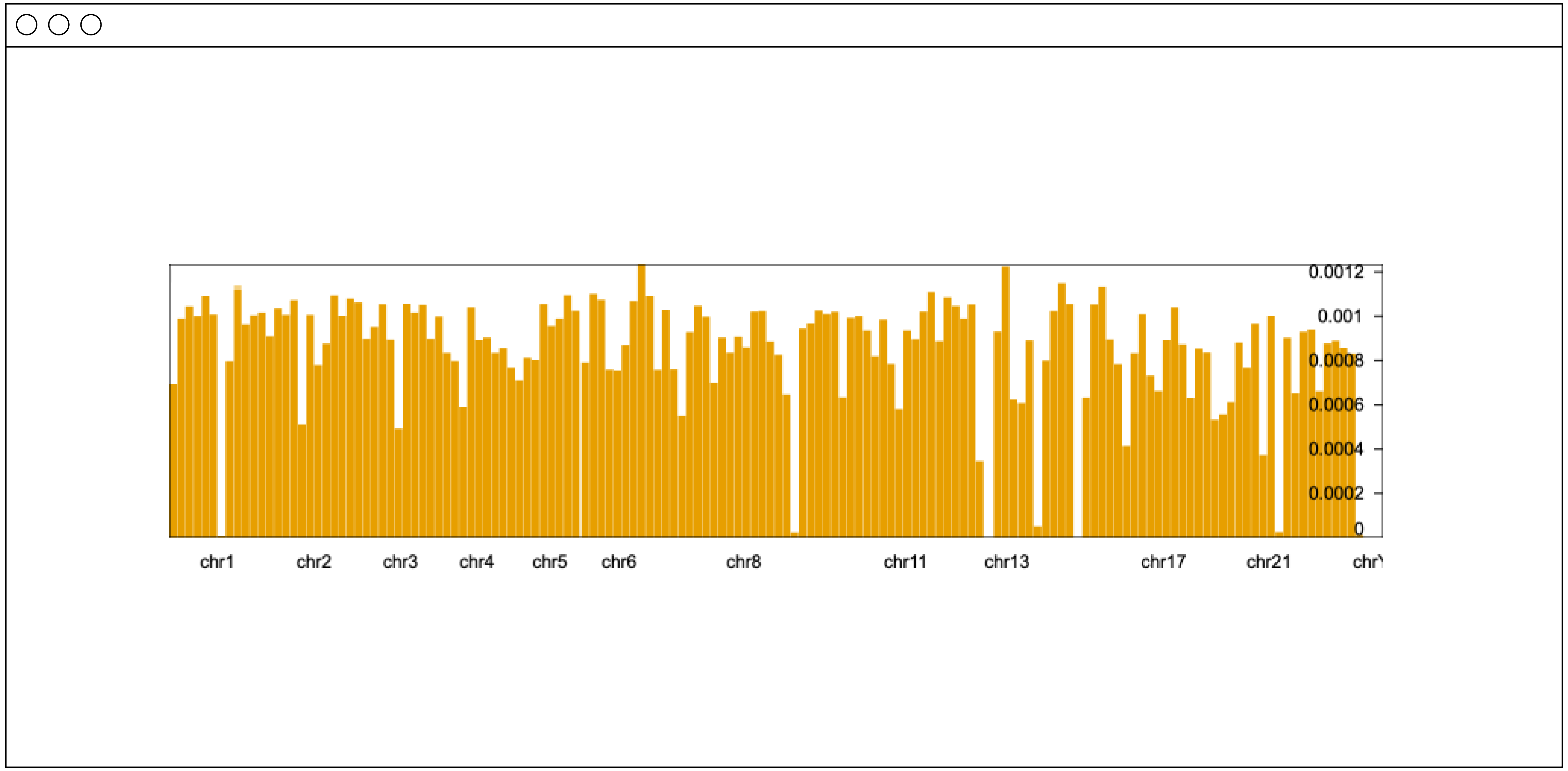

CHAT INPUT

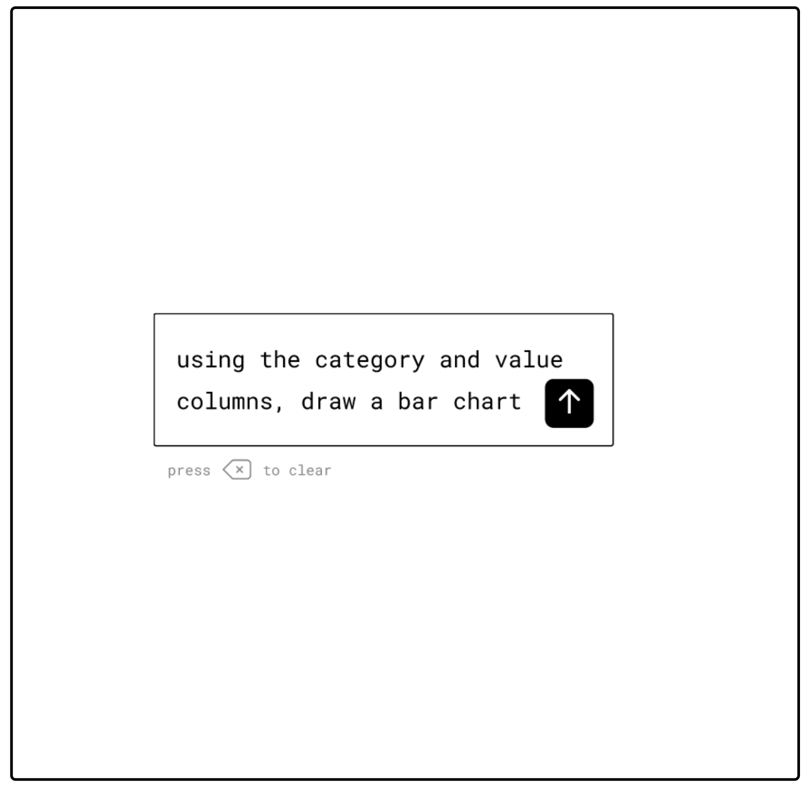

SHELF CONSTRUCTION

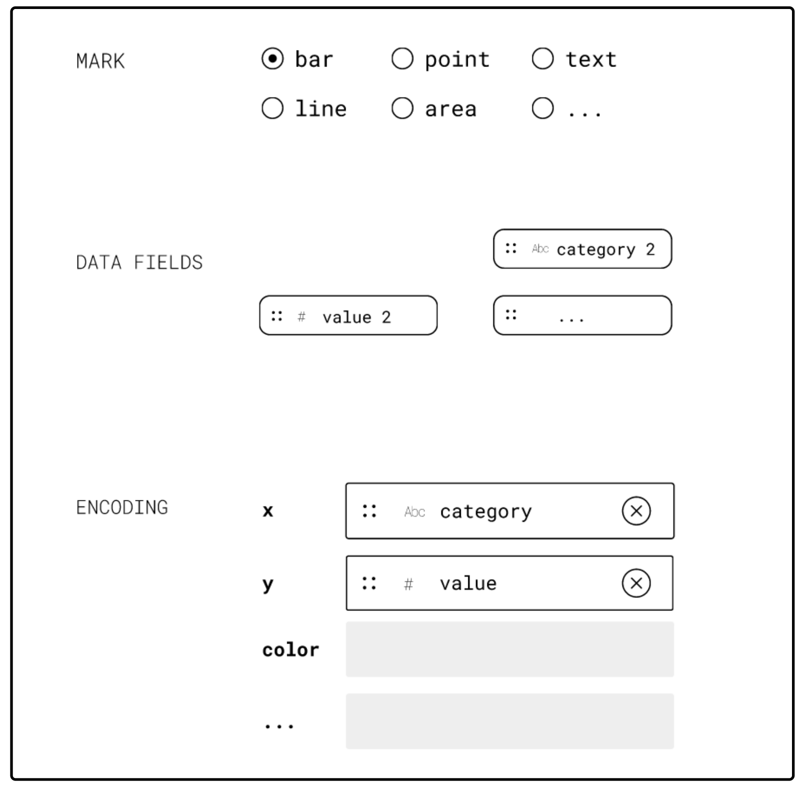

TEMPLATE SELECTION

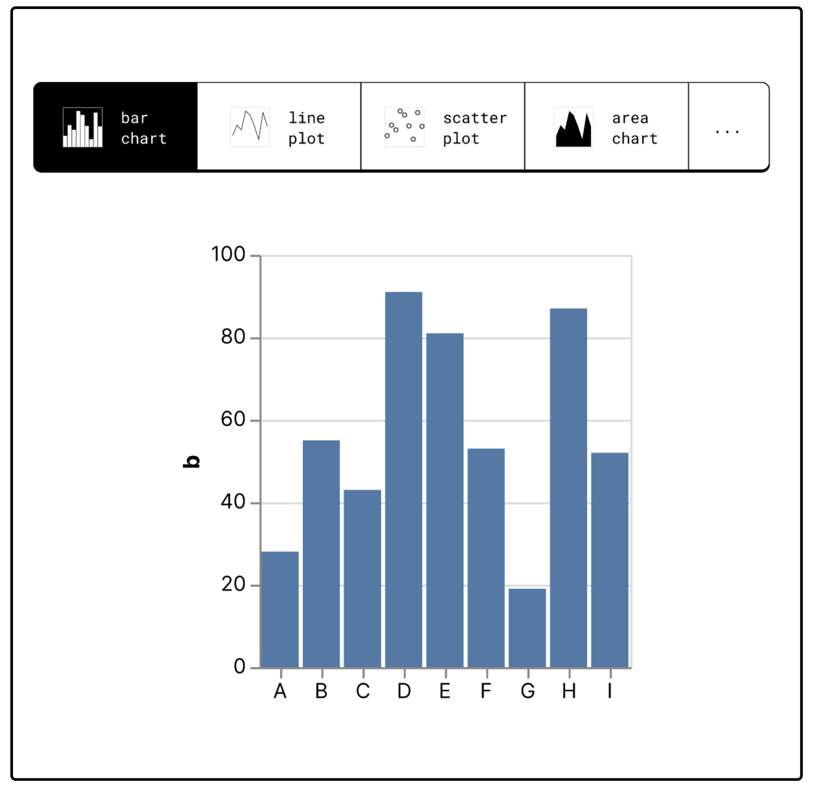

VISUALIZATION BY DEMONSTRATION

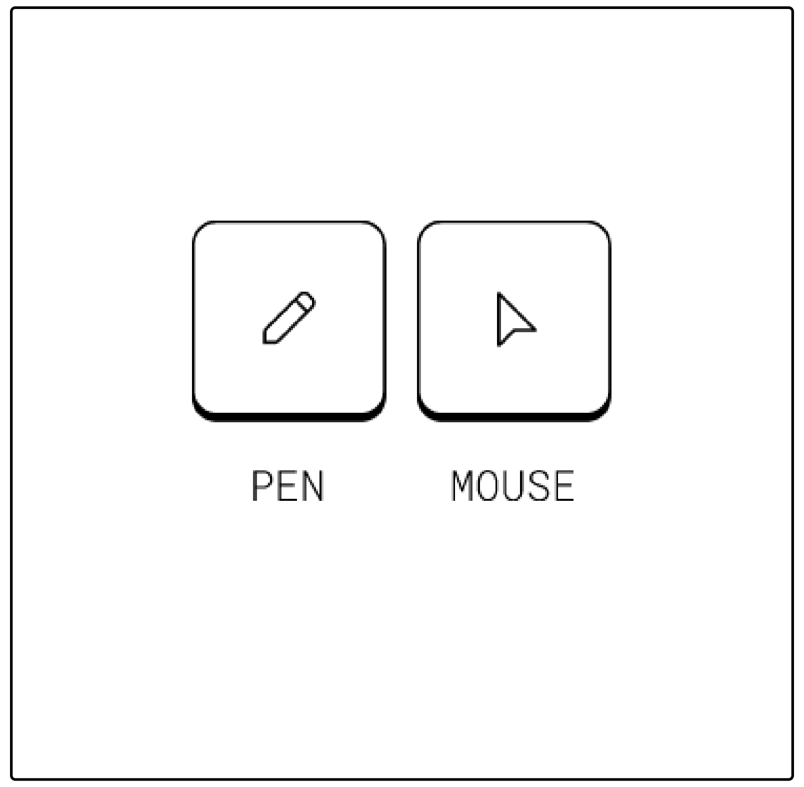

EXAMPLE VISUALIZATION UPLOAD

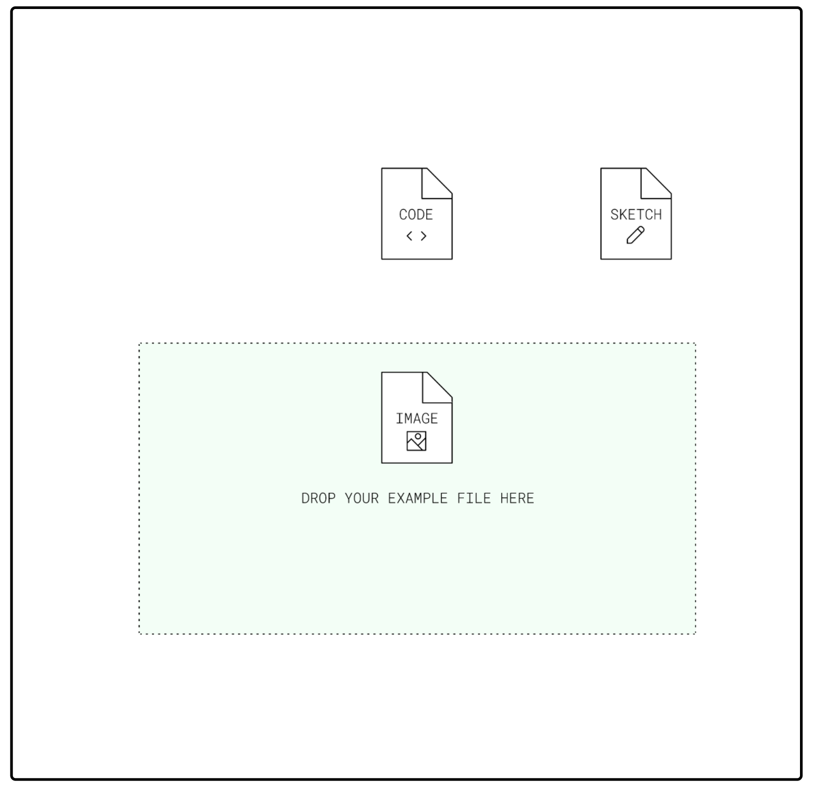

CODE INPUT

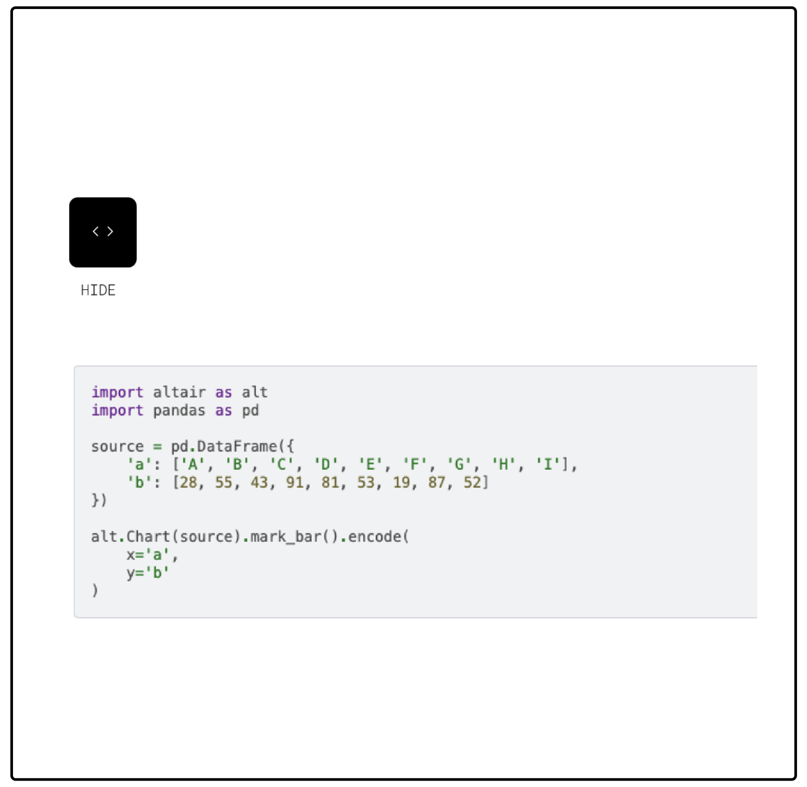

OTHER

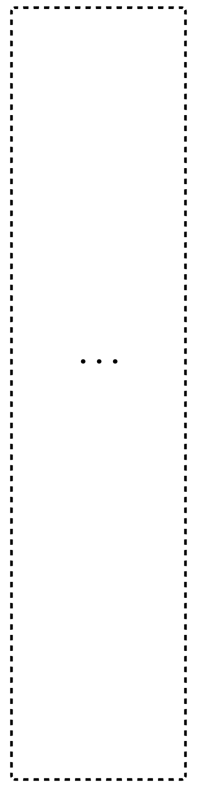

< Previous

> Next

4. Visualization modification

Select one of the following tasks that you perform frequently and select one or multiple interaction modalities to perform the task. You are also free to come up with your own task and modalities. Explain how you would go about it.

Change the type:

- *example task:* Change the visualization type to line plot
- *example task:* Change the visualization type to scatter plot
- ...

Change the layout:

- *example task:* Change the layout from linear (—) to circular (○)
- *example task:* Change the layout from linear (—) to Hilbert ( )
- ...

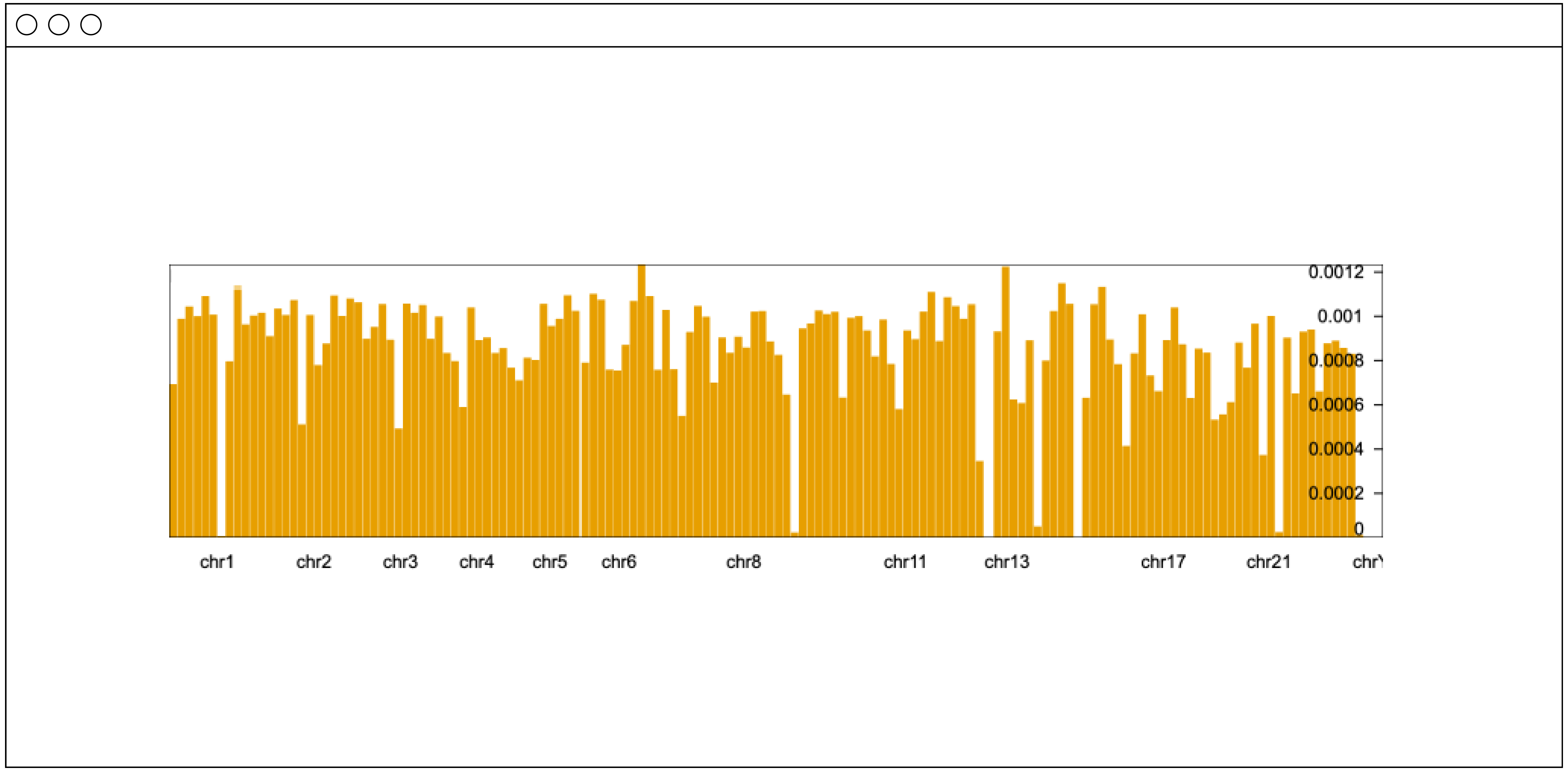

CHAT INPUT

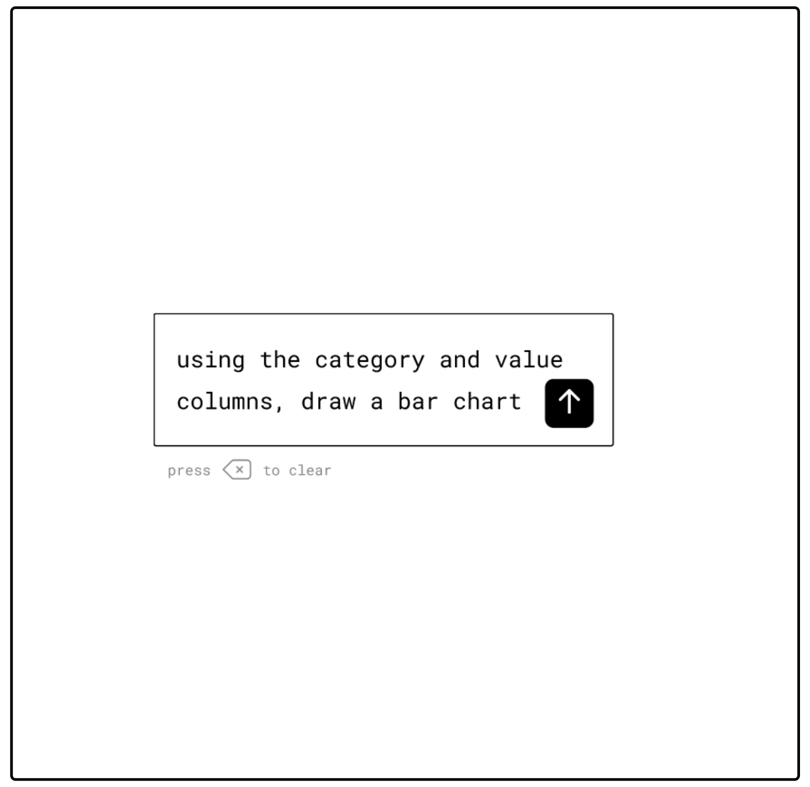

SHELF CONSTRUCTION

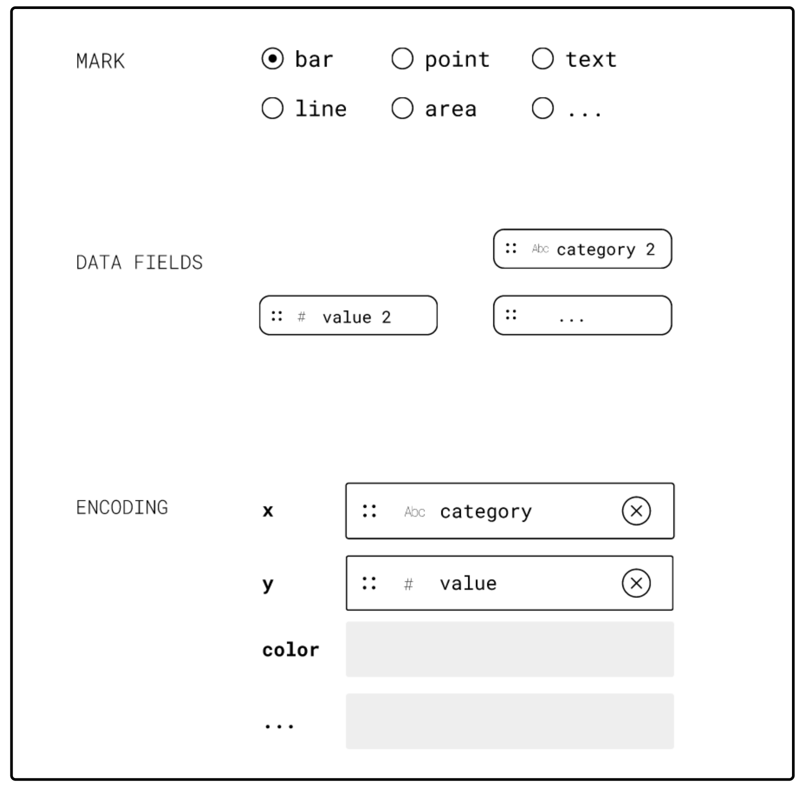

TEMPLATE SELECTION

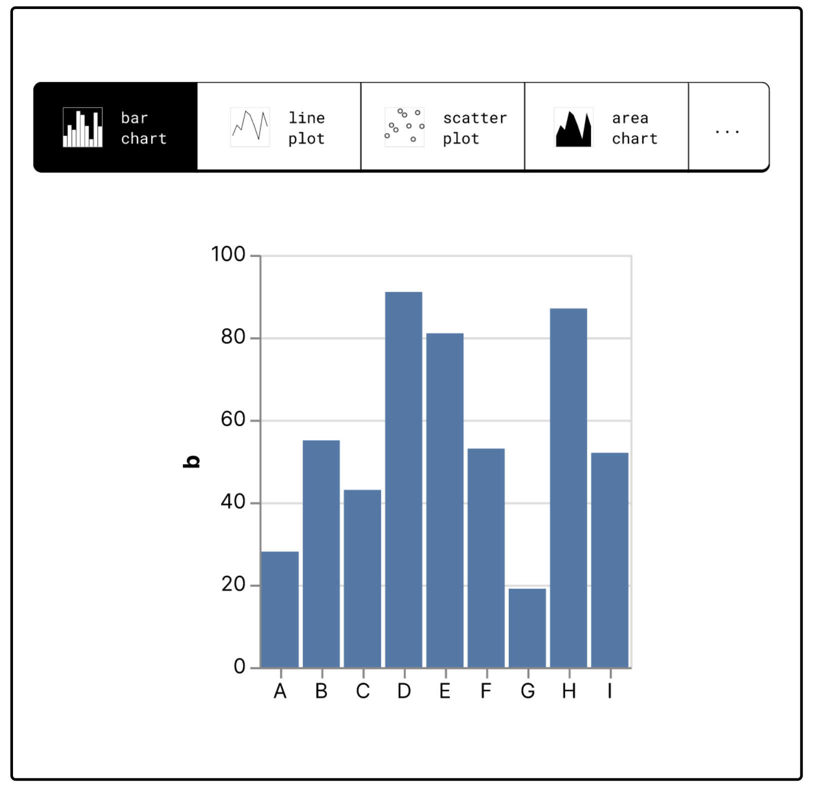

VISUALIZATION BY DEMONSTRATION

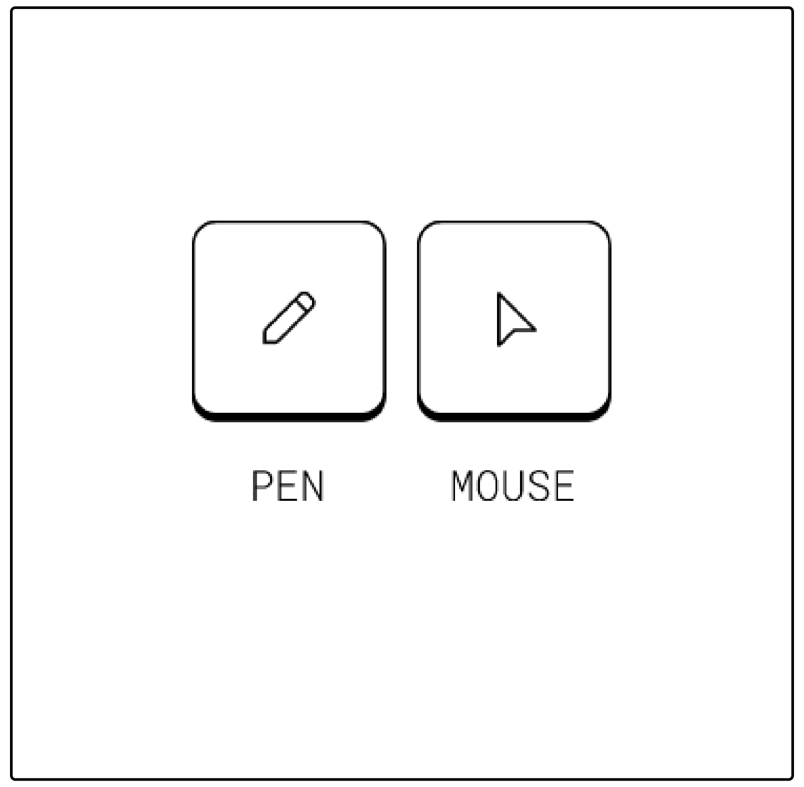

EXAMPLE VISUALIZATION UPLOAD

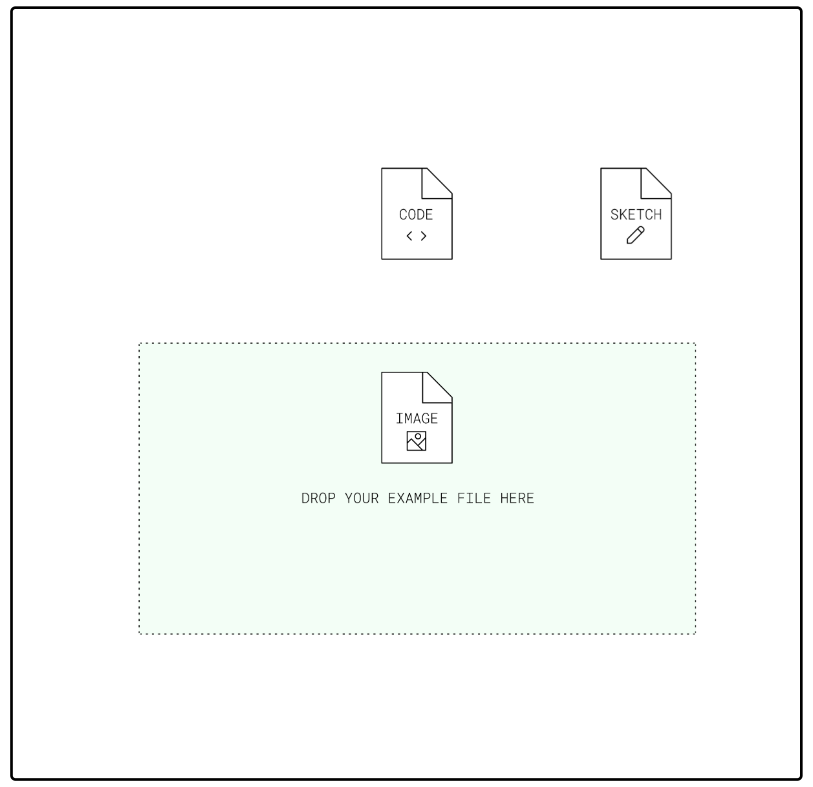

CODE INPUT

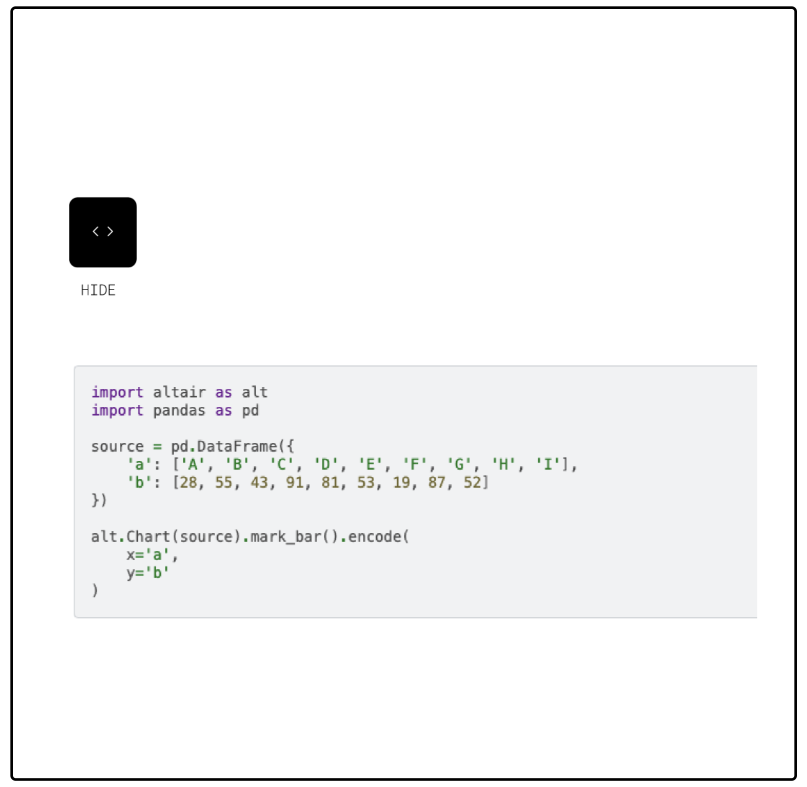

OTHER

...

< Previous

> Next

5. Add track(s) to the view

Select one of the following tasks that you perform frequently and select one or multiple interaction modalities to perform the task. You are also free to come up with your own task and modalities. Explain how you would go about it.

- *example task:* Add a track of the conservation score in the same ROI
- *example task:* Add a track of the gaps per position in this ROI
- ...

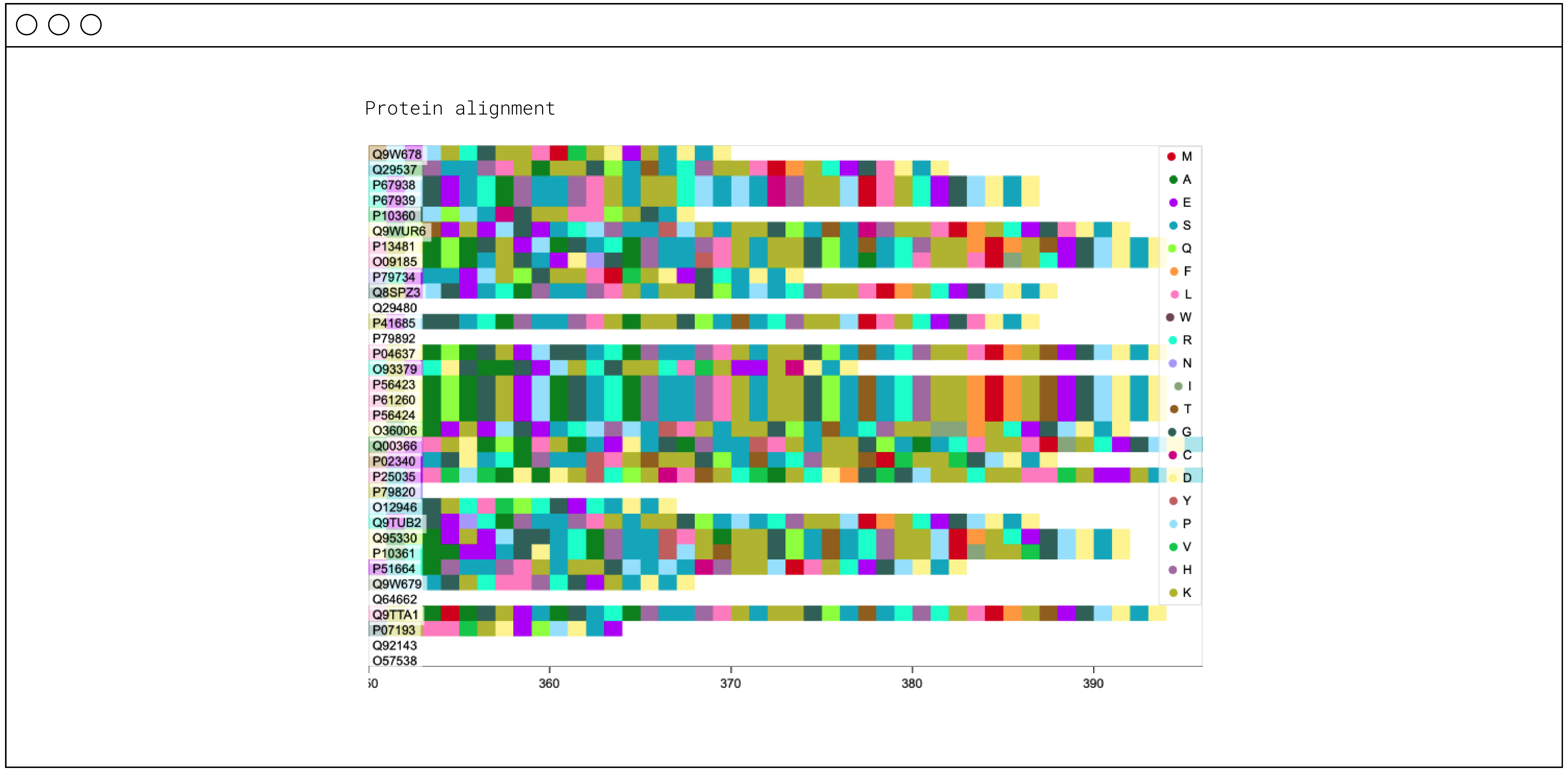

CHAT INPUT

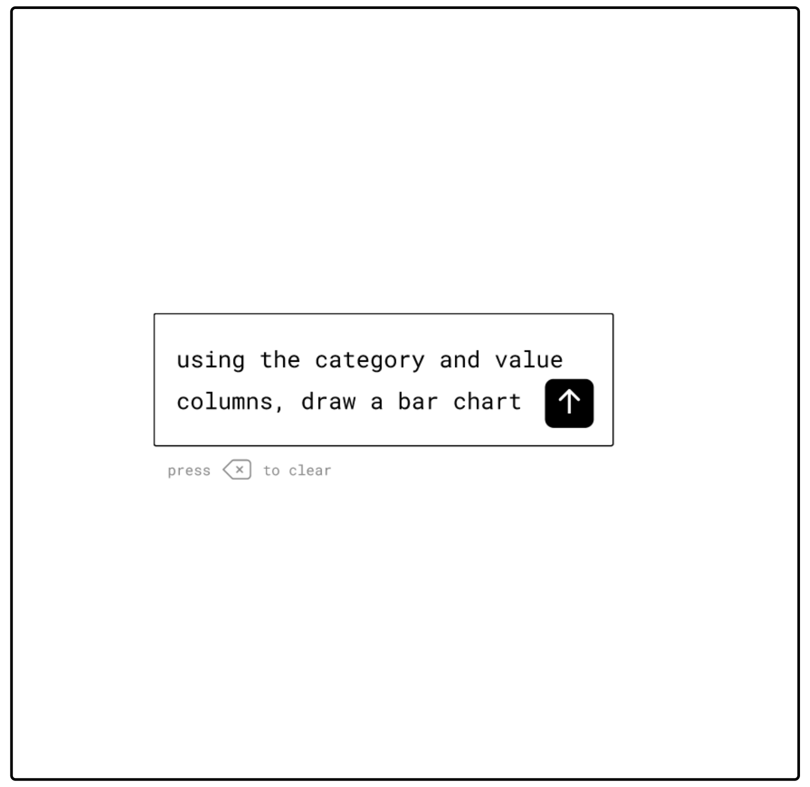

SHELF CONSTRUCTION

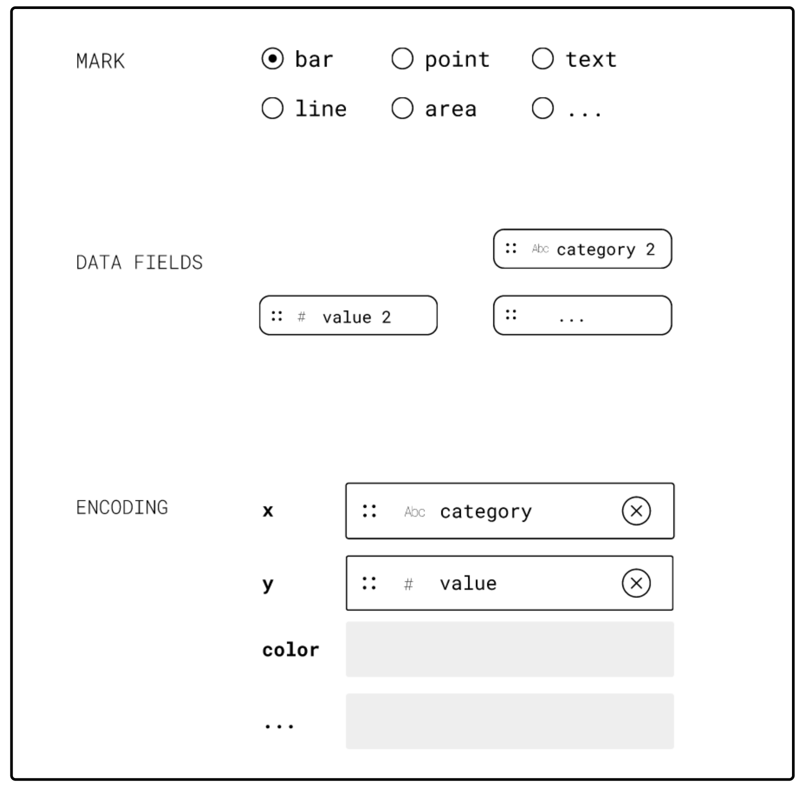

TEMPLATE SELECTION

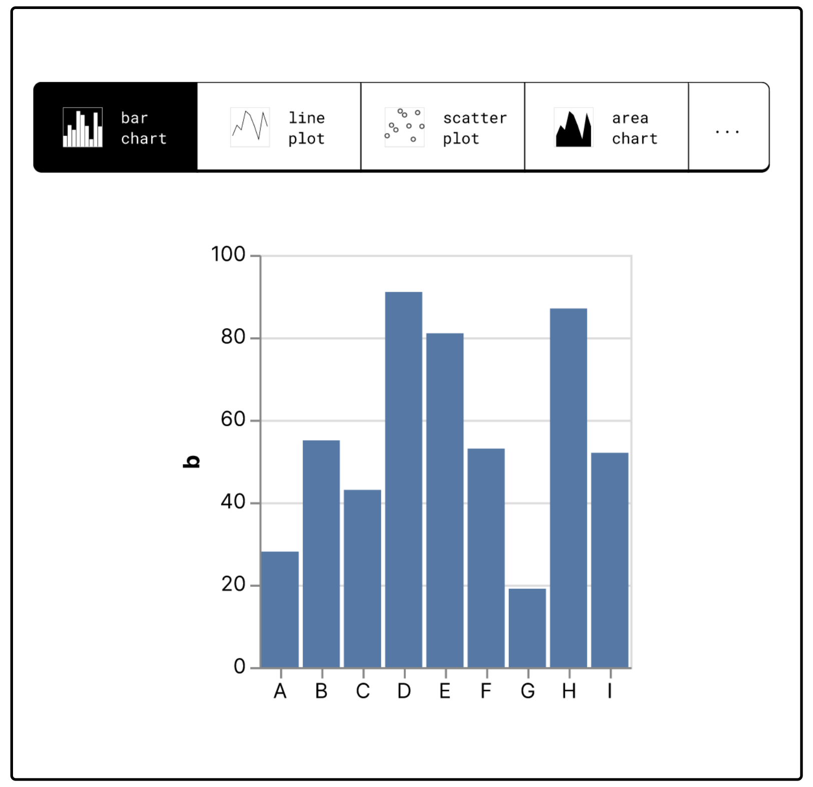

VISUALIZATION BY DEMONSTRATION

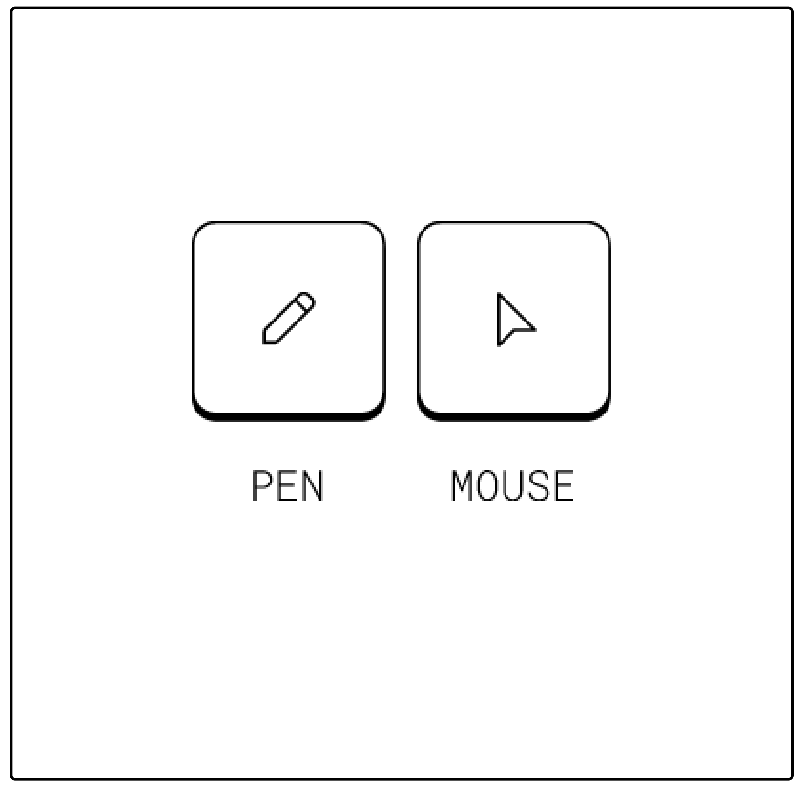

EXAMPLE VISUALIZATION UPLOAD

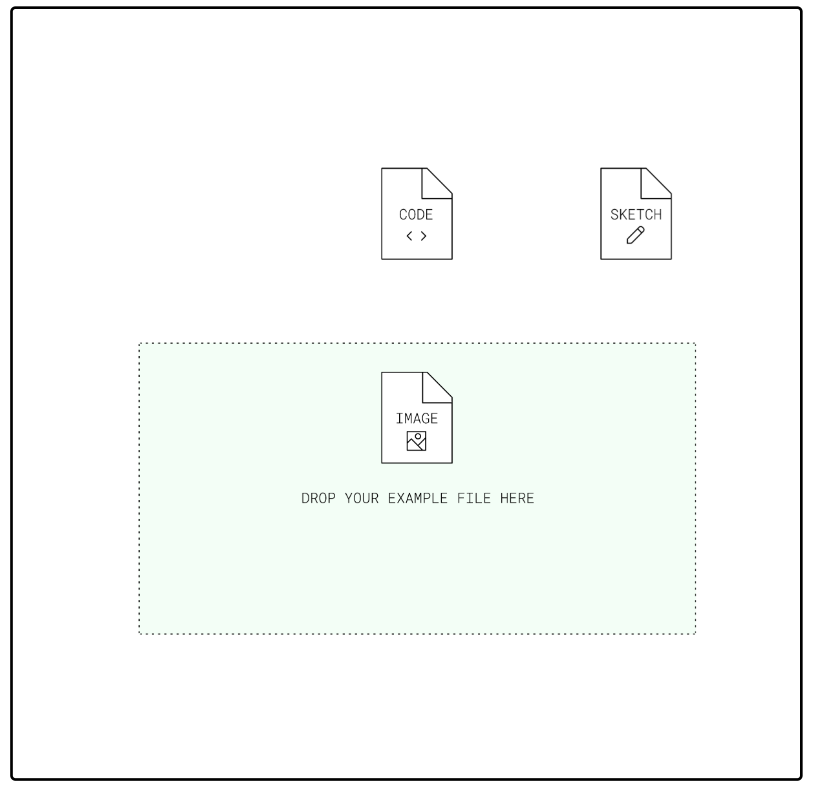

CODE INPUT

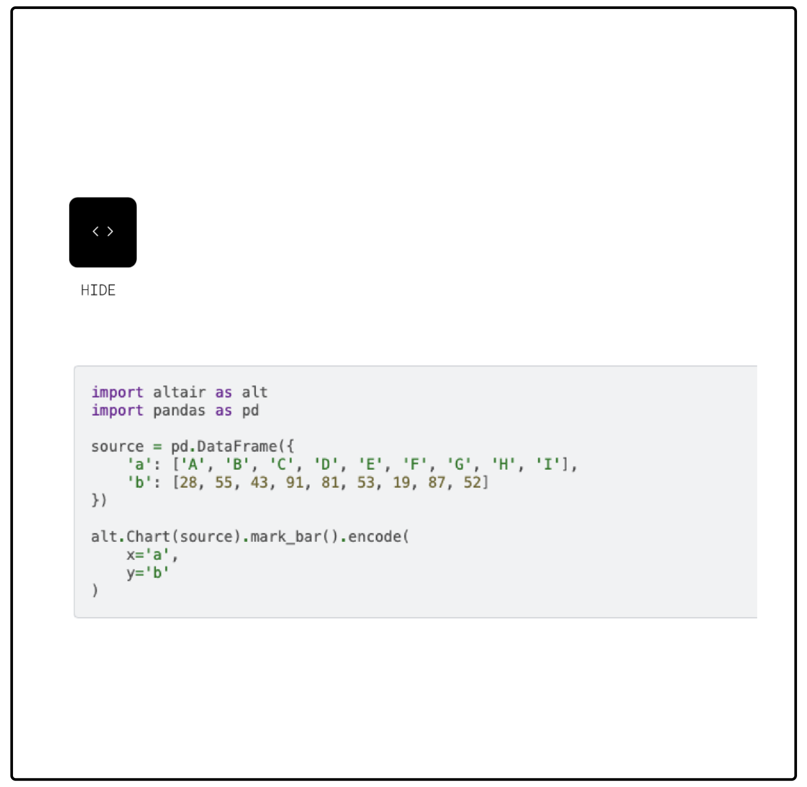

OTHER

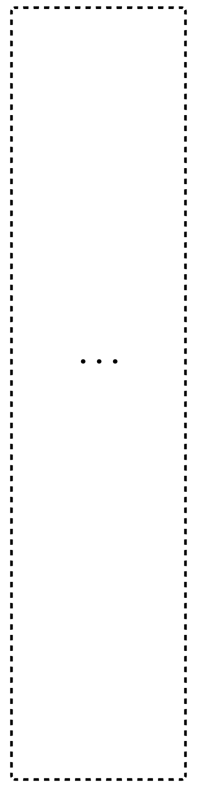

< Previous

> Next

6. Create multiple views

Select one of the following tasks that you perform frequently and select one or multiple interaction modalities to perform the task. You are also free to come up with your own task and modalities. Explain how you would go about it.

- *example task:* clone this visualization for another sample or ROI
- *example task:* Add a whole genome overview and create a brush to link to the detailed view
- ...

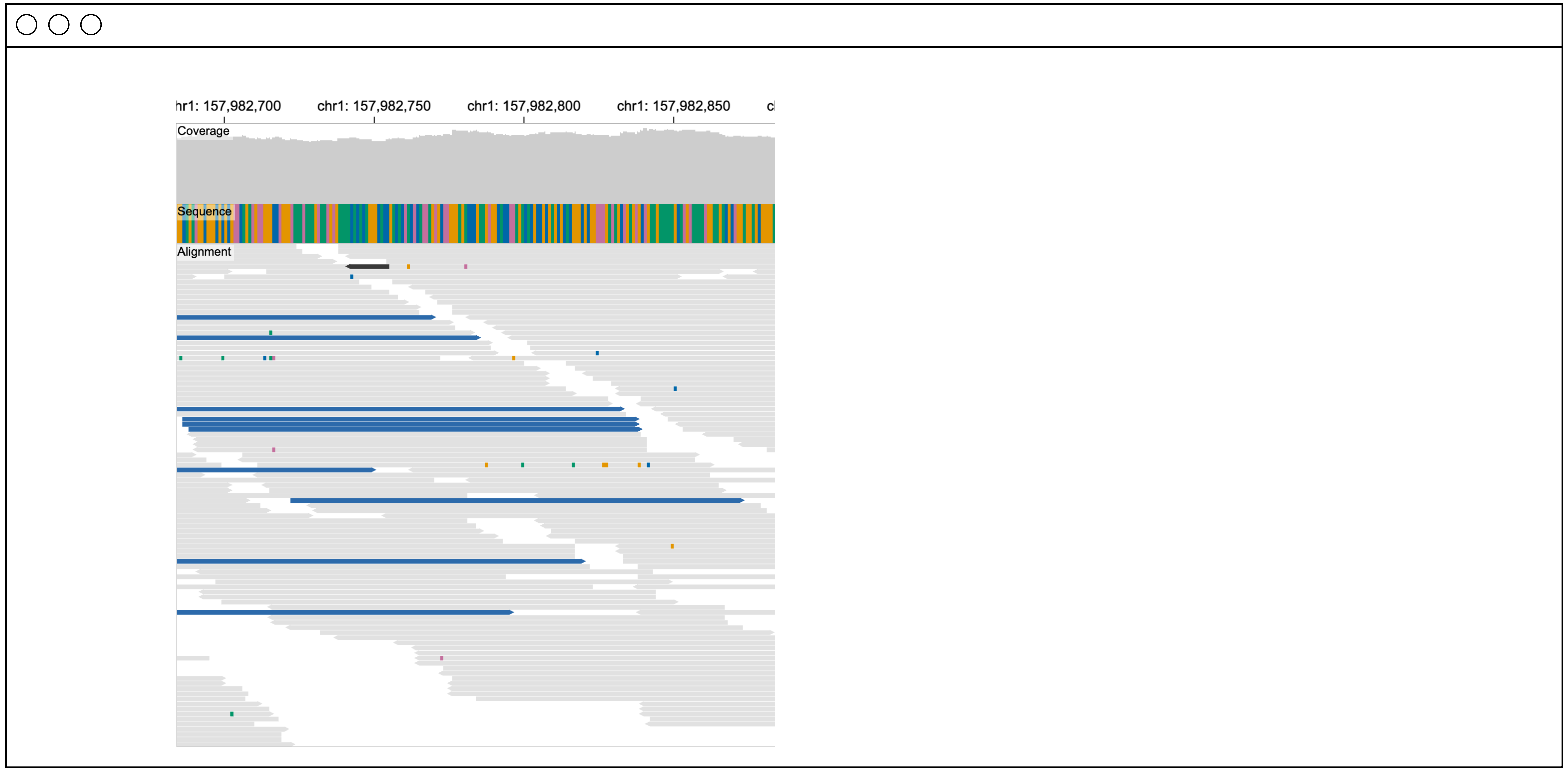

CHAT INPUT

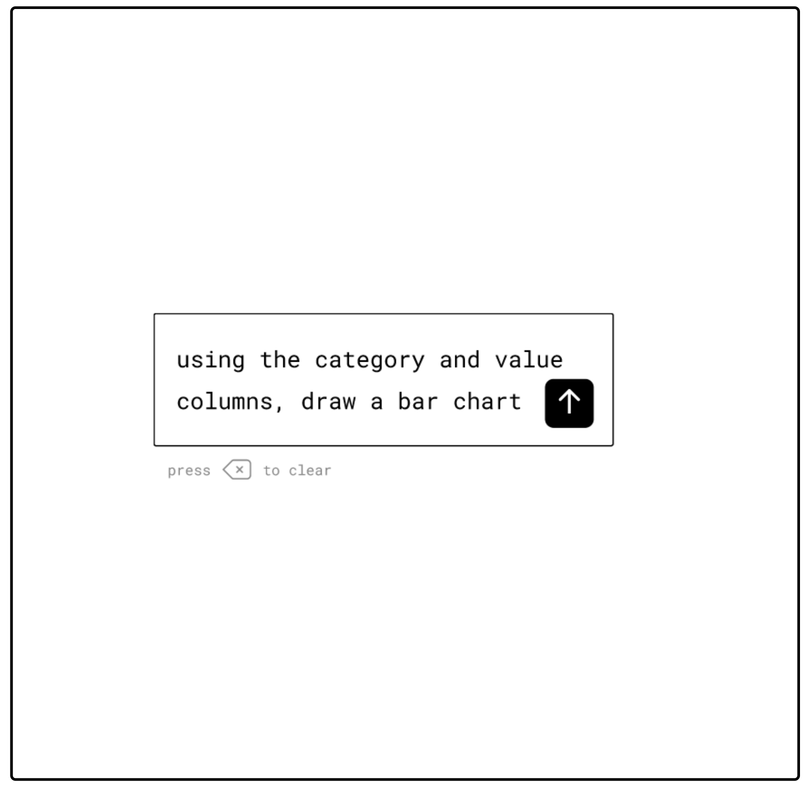

SHELF CONSTRUCTION

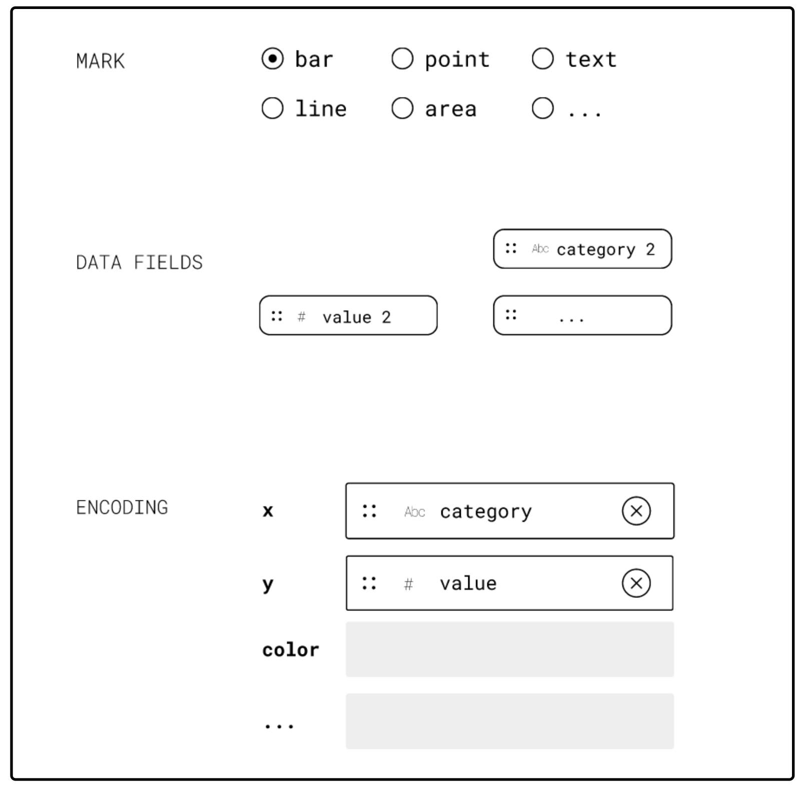

TEMPLATE SELECTION

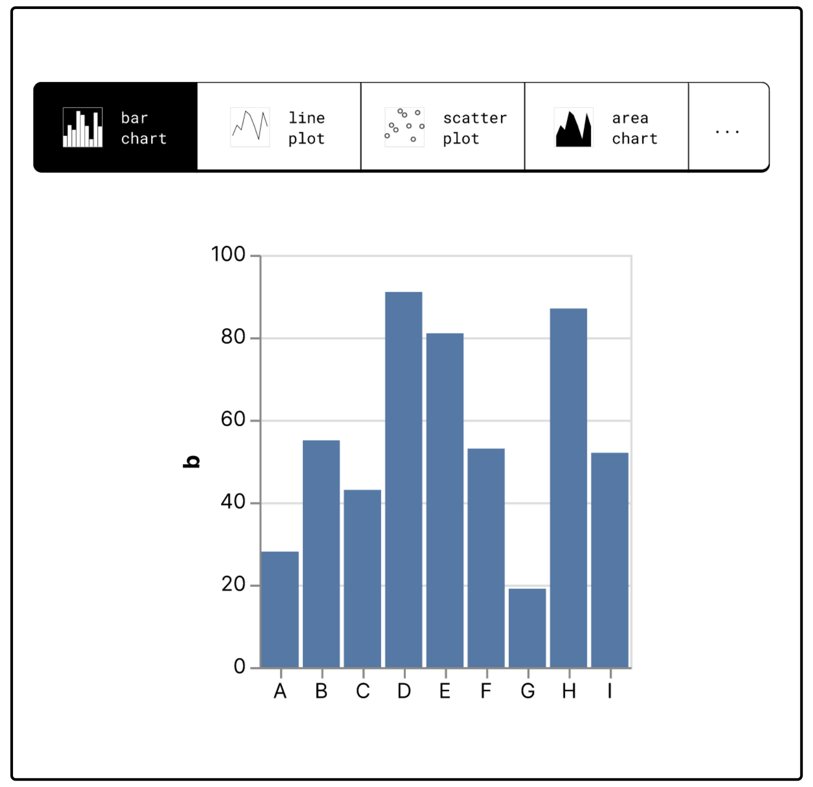

VISUALIZATION BY DEMONSTRATION

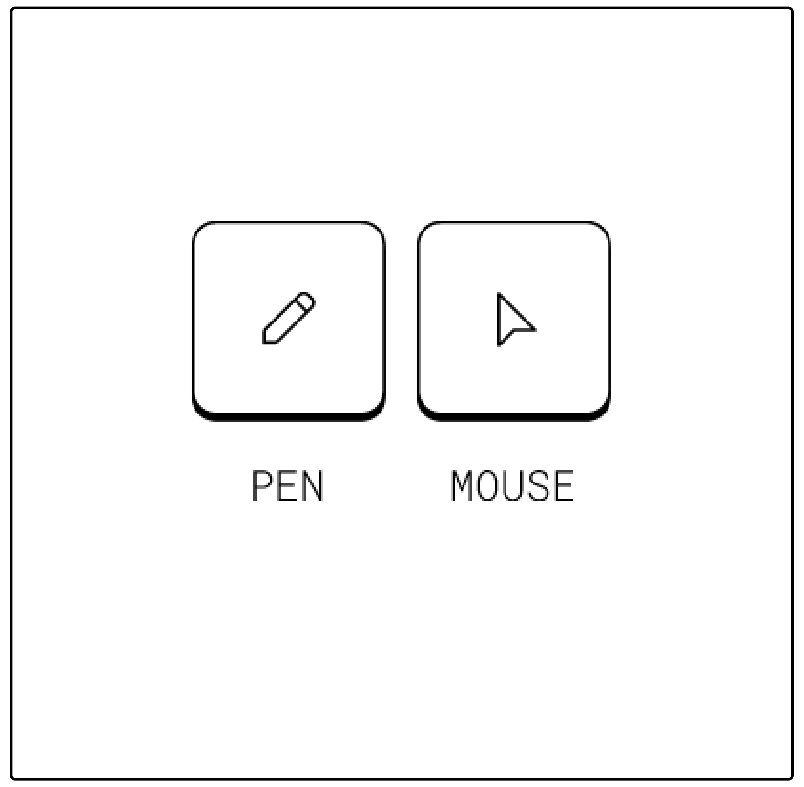

EXAMPLE VISUALIZATION UPLOAD

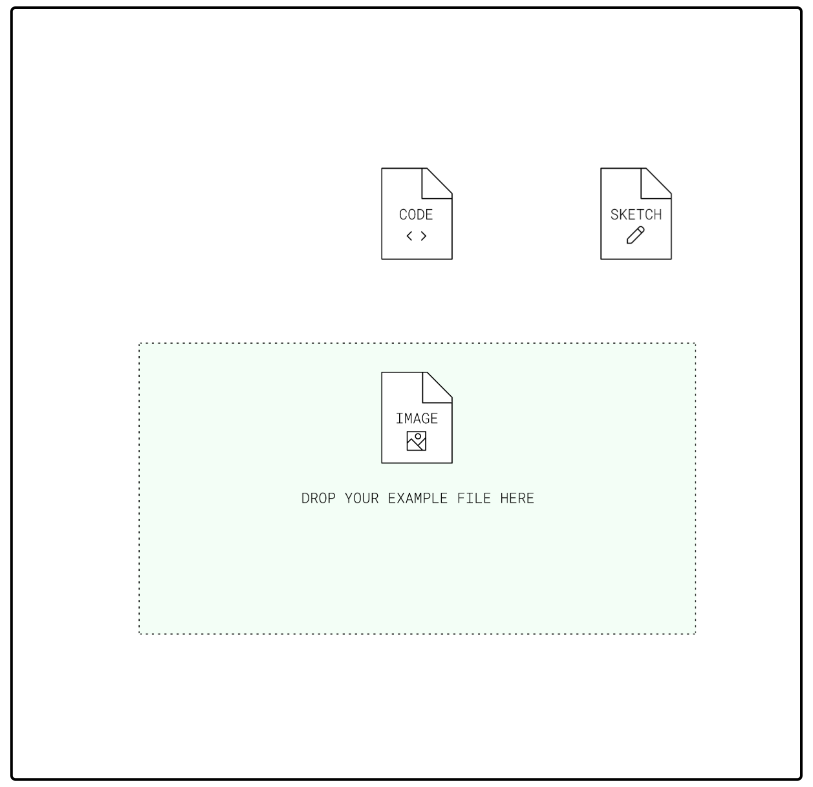

CODE INPUT

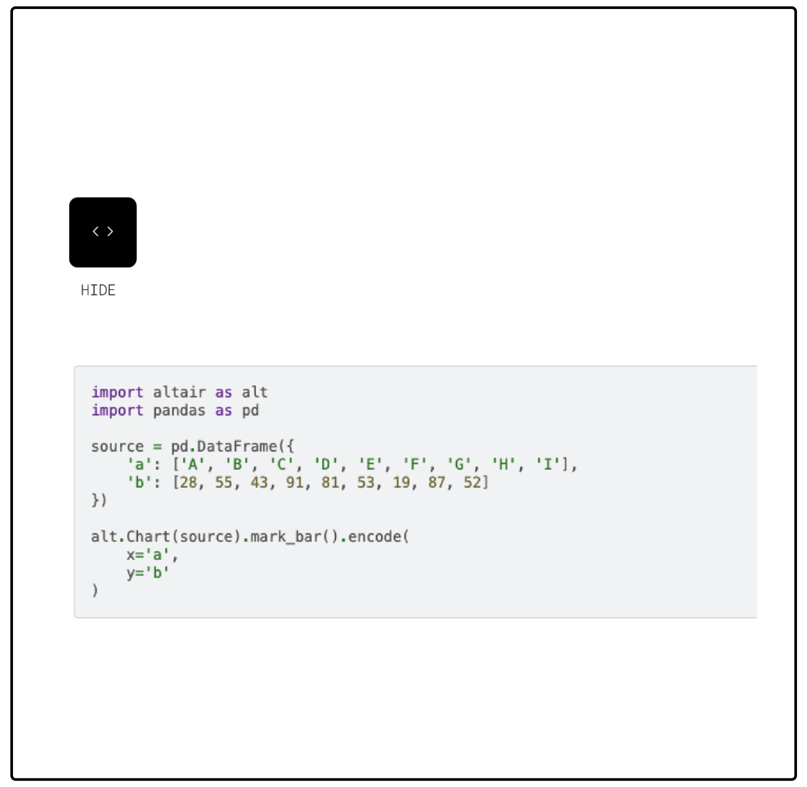

OTHER

...

< Previous

> Next

7. Change arrangement of views

Select one of the following tasks that you perform frequently and select one or multiple interaction modalities to perform the task. You are also free to come up with your own task and modalities. Explain how you would go about it.

- *example task:* Change the arrangement of the views to all vertically ( ) or horizontally ( ) juxtaposed
- *example task:* Change the arrangement of the views from juxtaposed ( ) to superimposed ( )
- ...

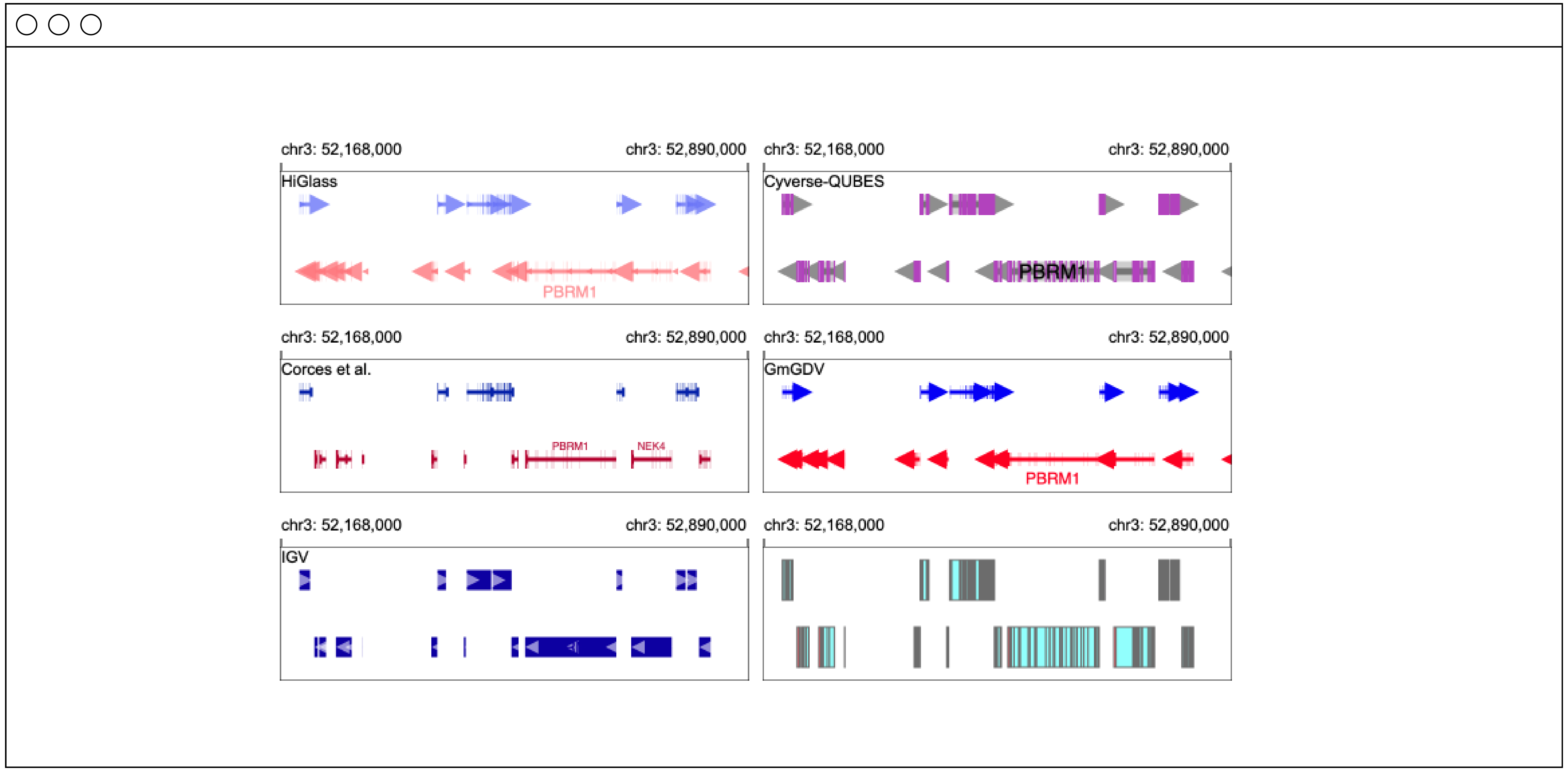

CHAT INPUT

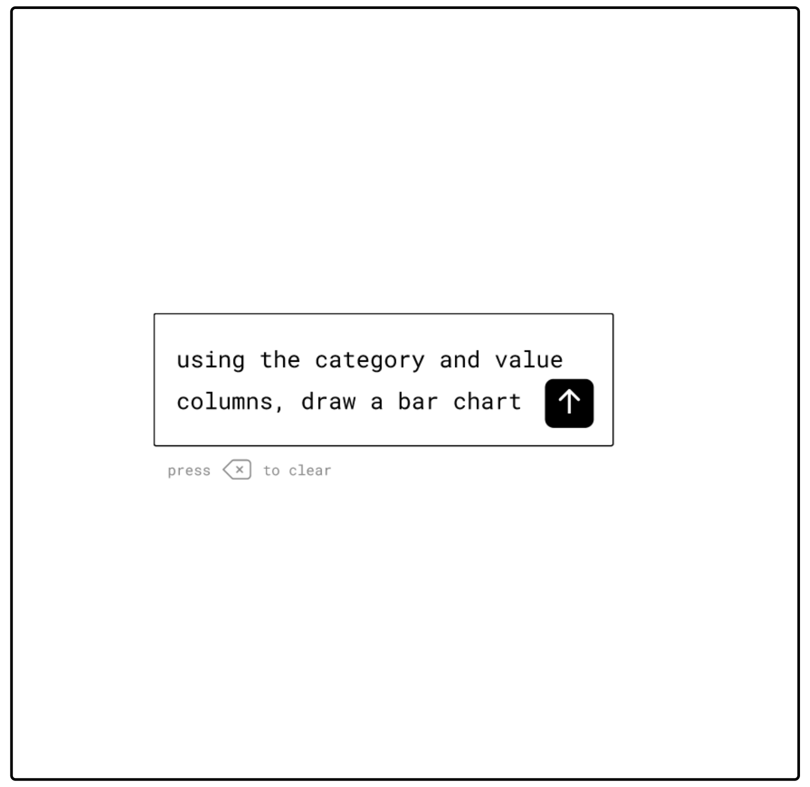

SHELF CONSTRUCTION

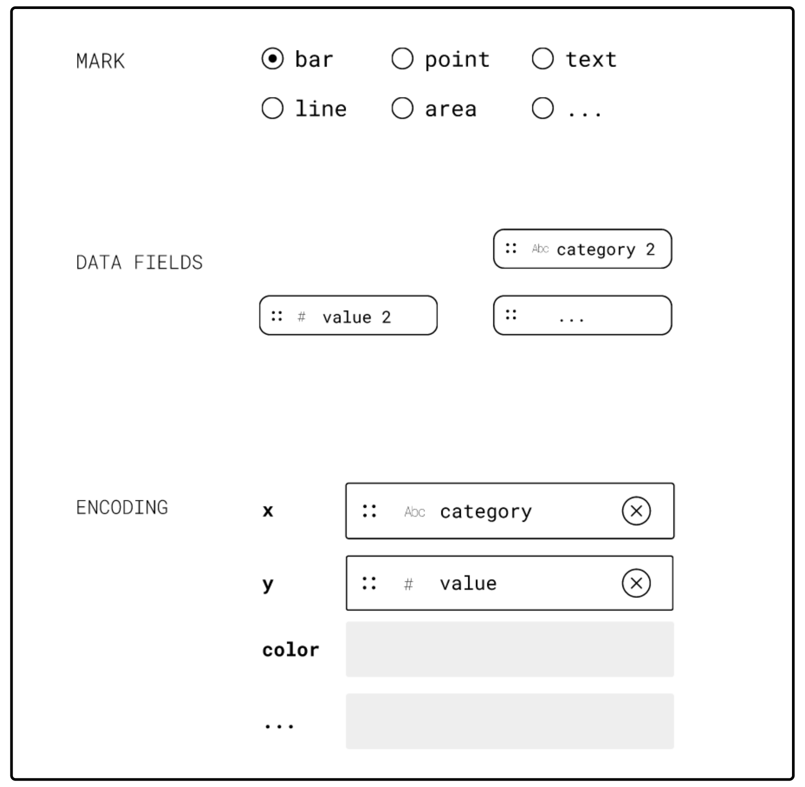

TEMPLATE SELECTION

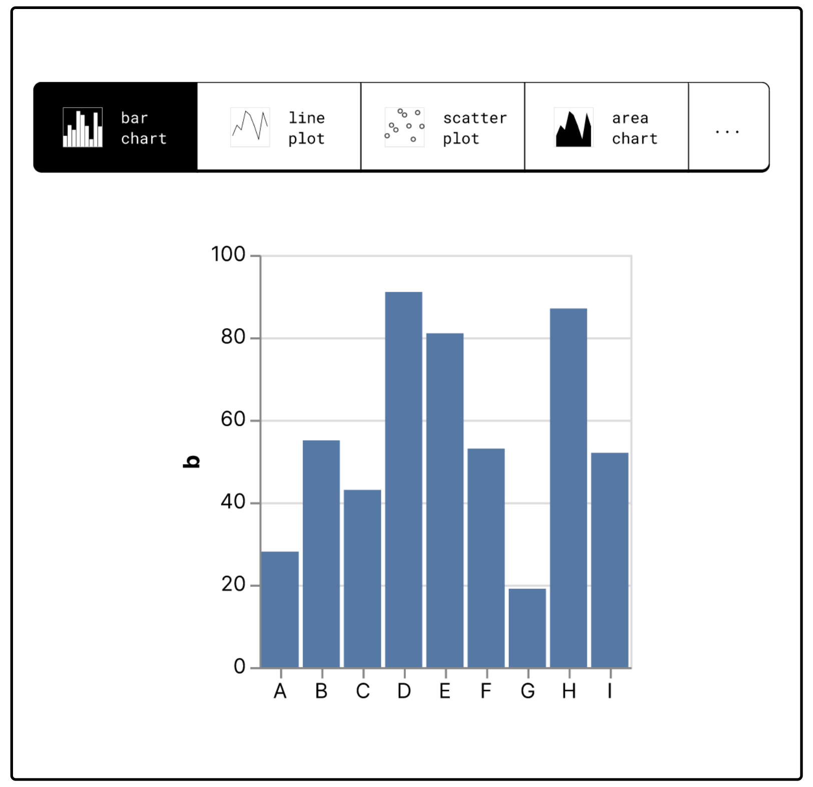

VISUALIZATION BY DEMONSTRATION

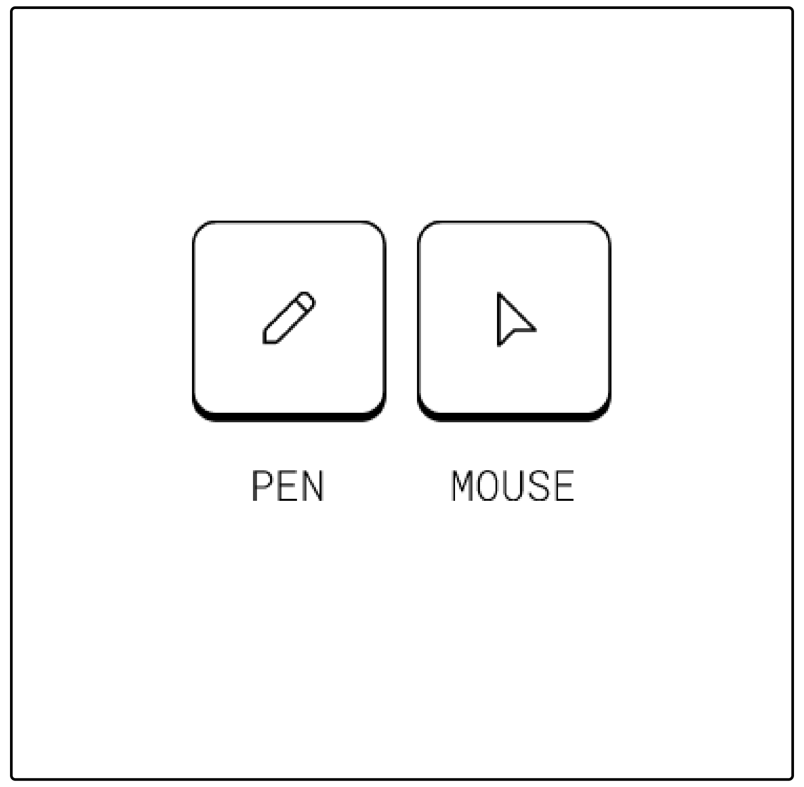

EXAMPLE VISUALIZATION UPLOAD

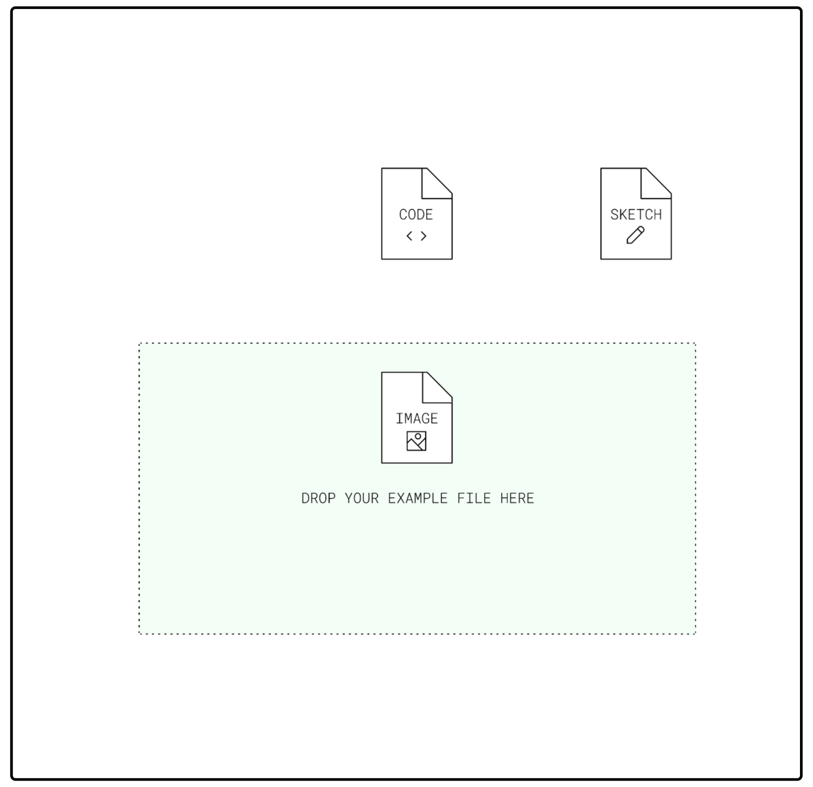

CODE INPUT

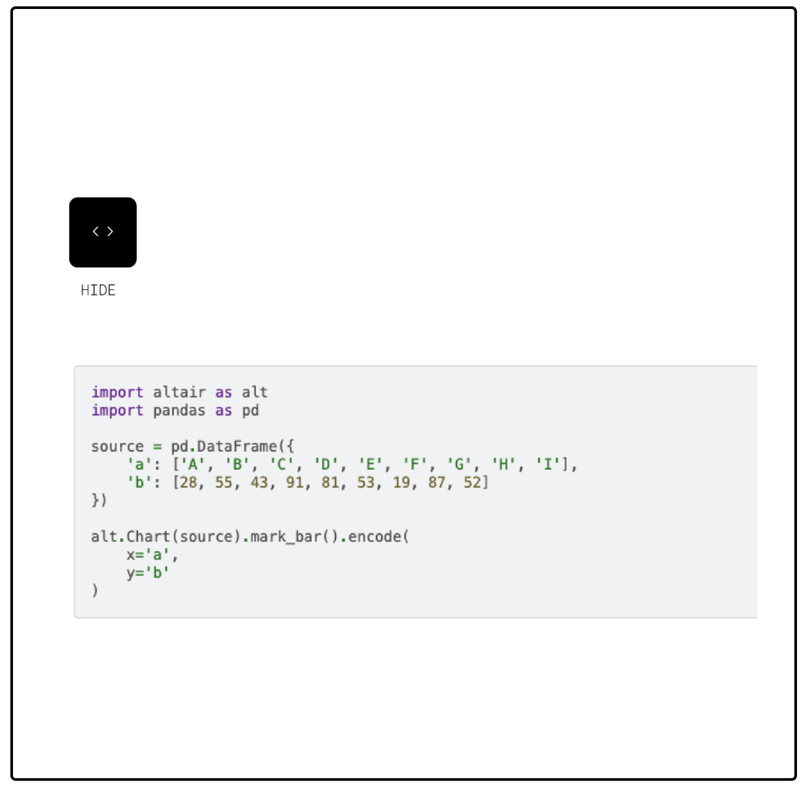

OTHER

...

< Previous

> Next

8. Custom genome annotation

Select one of the following tasks that you perform frequently and select one or multiple interaction modalities to perform the task. You are also free to come up with your own task and modalities. Explain how you would go about it.

- *example task: Create a custom genome annotation and add it to the view(s) to use this visualization as a figure in a paper*

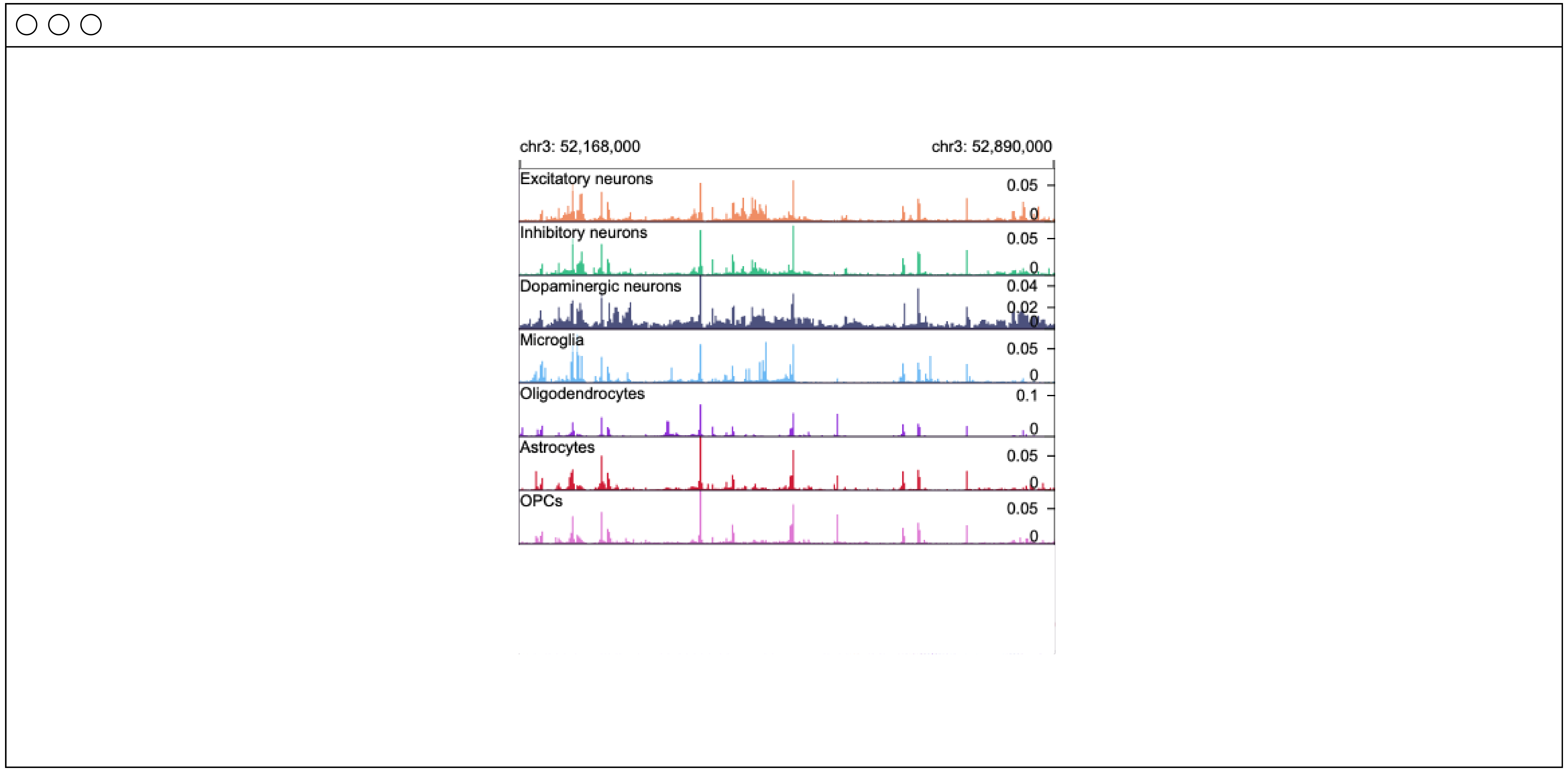

CHAT INPUT | SHELF CONSTRUCTION | TEMPLATE SELECTION | VISUALIZATION BY DEMONSTRATION | EXAMPLE VISUALIZATION UPLOAD | CODE INPUT | OTHER

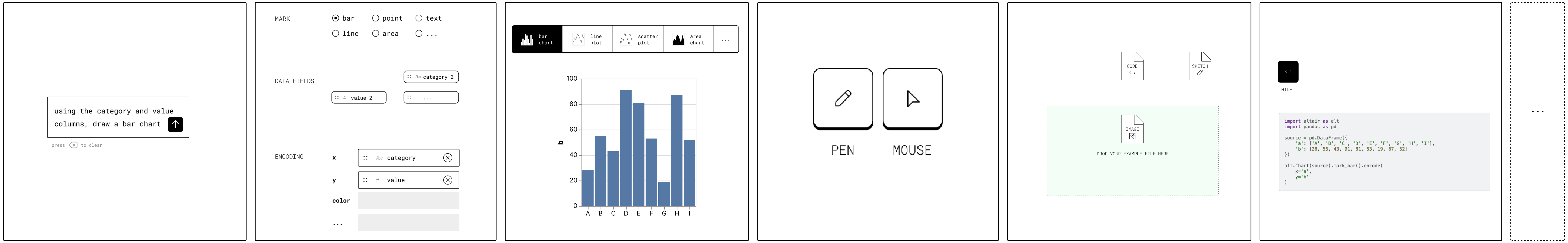

< Previous

> Next

🥳 You’ve gone through all the 8 probes

Please visit this link to continue:
https://forms.gle/ooe46EhMBfXb8ZAU6
