# Supplementary material for: Understanding Visualization Authoring Techniques for Genomics Data in the Context of Personas and Tasks
Source: IEEE Trans Vis Comput Graph. Author manuscript; Available in PMC 2025 Mar 4. (PMC11875953; doi:10.1109/TVCG.2024.3456298)
Supplement: tvcg-3456298-mm [file NIHMS2039885-supplement-tvcg-3456298-mm.zip › tvcg-3456298-mm/probe-circular-example-link.rtf]

https://www.figma.com/proto/koVriMeg4CZ1X6VKc5jies/Design-Probes-(C)---P5?node-id=26-90&starting-point-node-id=26%3A90&t=EXMzshOJ7W8VxRir-1
