# Supplementary material for: Understanding Visualization Authoring Techniques for Genomics Data in the Context of Personas and Tasks
Source: IEEE Trans Vis Comput Graph. Author manuscript; Available in PMC 2025 Mar 4. (PMC11875953; doi:10.1109/TVCG.2024.3456298)
Supplement: tvcg-3456298-mm [file NIHMS2039885-supplement-tvcg-3456298-mm.zip › tvcg-3456298-mm/README.pdf]

DESCRIPTION: The supplemental material contains material that is not included within the article itself.

SIZE: 12.4 MB

PLAYER INFORMATION: .txt, .pdf, .rtf, .csv

PACKING LIST: tvcg-3456298-mm.zip

CONTACT INFORMATION:

Astrid van den Brandt
Eindhoven University of Technology
Email: a.v.d.brandt@tue.nl
