# Supplementary material for: Understanding Visualization Authoring Techniques for Genomics Data in the Context of Personas and Tasks
Source: IEEE Trans Vis Comput Graph. Author manuscript; Available in PMC 2025 Mar 4. (PMC11875953; doi:10.1109/TVCG.2024.3456298)
Supplement: tvcg-3456298-mm [file NIHMS2039885-supplement-tvcg-3456298-mm.zip › tvcg-3456298-mm/study2_checklist.pdf]

Before session

- [ ] Change the Zoom name to P# (e.g., P1)
- [ ] Enable automatic transcription
- [ ] Enable screen sharing by multiple participants

Introduction slides (2 min)

- [ ] Definition of interaction modality
- [ ] Ask permission to record

Training phase (18 min)

- [ ] Tell what will happen: first we explain the modality, then watch video, finally DIY
- [ ] Share link figma training via Zoom
    - [ ] https://www.figma.com/proto/wl4wv5QARDnRXZAj3lvVhS/Training-Material?node-id=2342-2894&starting-point-node-id=2342%3A2894&t=Qn23pvgpkkoWXsIK-1
- [ ] Ask participant to share the screen
    - [ ] Ask to press Z for fit to screen
- [ ] End of training: share test on modalities
    - [ ] https://forms.gle/HyqLT1Jfi3GNcaQD7
    - [ ] Encourage participant to read captions before making choice
    - [ ] Research team quickly checks the answers of the participant and if needed provide extra explanation

Test Probe phase (40 min)

- [ ] Tell what will happen: we will go through 8 probes, this will guide you through
    - [ ] If anything is unclear, ask
- [ ] Share link figma test via Zoom
- [ ] Ask participant to share the screen
    - [ ] Ask to press Z for fit to screen
- [ ] Ask to spotlight participant in Figma
- [ ] End of training: share link for survey on modalities
    - [ ] https://forms.gle/ooe46EhMBfXb8ZAU6
