# Supplementary material for: Understanding Visualization Authoring Techniques for Genomics Data in the Context of Personas and Tasks
Source: IEEE Trans Vis Comput Graph. Author manuscript; Available in PMC 2025 Mar 4. (PMC11875953; doi:10.1109/TVCG.2024.3456298)
Supplement: tvcg-3456298-mm [file NIHMS2039885-supplement-tvcg-3456298-mm.zip › tvcg-3456298-mm/study2_figma_links_setup.pdf]

Study slides:
https://docs.google.com/presentation/d/1H8ROHmd9ZNxTRNPEkwiqdvl9Wd2QXF-YsnnZlofVGX4/edit?usp=sharing

Training figma:
https://www.figma.com/proto/wl4wv5QARDnRXZAj3lvVhS/Training-Material?node-id=2342-2894&starting-point-node-id=2342%3A2894&mode=design&t=Qn23pvgpkkoWXsIK-1

Google form post-training: https://forms.gle/2YUQFoGYjPP3oUPJ7

Google form post-probes: https://forms.gle/ooe46EhMBfXb8ZAU6

P13	Linear/ Circular	Shelf - Chat - VbD - Code - Example - Template	C: https://www.figma.com/proto/iIlKe1c0SaVFa2gcUtk8bw/Design-Probes-(C)---P13?type=design&node-id=26-90&t=KkUA09I3XrxIhIPU-1&scaling=contain&page-id=0%3A1&starting-point-node-id=26%3A90&mode=design<br><br>~~L: https://www.figma.com/proto/7pFdZQ25TbqvD2YeD5ti2k/Design-Probes-(L)---P13?type=design&t=xuv9XbWmCpl1PBbz-1&scaling=contain&page-id=0%3A1&node-id=26-90&starting-point-node-id=26%3A90&mode=design~~
P12	Linear	Chat - Shelf - Template - VbD - Example - Code	https://www.figma.com/proto/nIDbxQMijk0c6rSKFRFDMj/Design-Probes-(L)---P12?type=design&node-id=26-90&t=zBguEk0UaplENT54-1&scaling=contain&page-id=0%3A1&starting-point-node-id=26%3A90&mode=design
P11	Linear	Example - Shelf - Code - Chat - Template - VbD	https://www.figma.com/proto/k5rHMhzGPZ7W7cZHTssFzi/Design-Probes-(L)---P11?type=design&node-id=3-3&t=ieYut7UEKTTxtffX-1&scaling=min-zoom&page-id=0%3A1&starting-point-node-id=26%3A90&mode=design
P10	Circular	Template - Example - VbD - Code - Shelf - Chat	https://www.figma.com/proto/kD3QqOzbPYF7A7xk140Vgi/Design-Probes-(C)---P10?type=design&node-id=26-90&t=9nG30aUAl0R2r0FL-1&scaling=contain&page-id=0%3A1&starting-point-node-id=26%3A90&mode=design

P9	Linear/ Circular	VbD - Code - Template - Example - Shelf - Chat	L: https://www.figma.com/proto/Pm7uatesna7DKvIE3DSfHH/Design-Probes-(L)---P9?type=design&node-id=26-90&t=EVNTSjDORqwFfePW-1&scaling=scale-down-width&page-id=0%3A1&starting-point-node-id=26%3A90&mode=design<br><br>~~C: https://www.figma.com/proto/BIxDj6IPVWKxajpYI5hsHf/Design-Probes-(C)---P9?type=design&node-id=26-90&t=jr5C5IwCik4IqGfp-1&scaling=contain&page-id=0%3A1&starting-point-node-id=26%3A90&mode=design~~
P8	Linear/ ~~Circular~~	Example - Code - Chat - VbD - Shelf - Template	L: https://www.figma.com/proto/Pip95vbUI7sDCGPS6EIiuY/Design-Probes-(L)---P8?type=design&node-id=26-90&t=Tik4rM2DXP8oF2AB-1&scaling=contain&page-id=0%3A1&starting-point-node-id=26%3A90&mode=design<br>~~C: https://www.figma.com/proto/vnYGP7vmskw93iwQWxVfZT/Design-Probes-(C)---P8?type=design&node-id=26-90&t=jMbYB3uiVp1ZWMmz-1&scaling=contain&page-id=0%3A1&starting-point-node-id=26%3A90&mode=design~~
P7	Linear	Shelf - Chat - VbD - Example - Template - Code	https://www.figma.com/proto/i2QweMK7nvmJxZd3fXOajO/Design-Probes-(L)---P7?type=design&t=GMfkCmktILTk1qkU-1&scaling=contain&page-id=0%3A1&node-id=26-90&starting-point-node-id=26%3A90&mode=design
P6	Linear	VbD - Example - Template - Shelf - Chat - Code	https://www.figma.com/proto/ED6Qg7EDLBDaEYkYbAQVDS/Design-Probes-(L)---P6?type=design&node-id=26-90&t=3EEMe9D5oX8wO9IY-1&scaling=contain&page-id=0%3A1&starting-point-node-id=26%3A90&mode=design
P5	Circular	Code - Shelf - Template - VbD - Chat - Example	https://www.figma.com/proto/koVriMeg4CZ1X6VKc5jies/Design-Probes-(C)---P5?type=design&node-id=26-90&t=0i4uCnhBv2YyawOy-1&scaling=contain&page-id=0%3A1&starting-point-node-id=26%3A90&mode=design
P4	Linear	Chat - Example - Template - Code - Shelf - VbD	https://www.figma.com/proto/hPKIN52VspC3HRDBGAUcr5/Design-Probes-(L)---P4?type=desig

			n&node-id=26-90&t=O69xt7J0l0rAXye9-1&scaling=contain&page-id=0%3A1&starting-point-node-id=26%3A90&mode=design
P3	Linear	Example - Shelf - Template - VbD - Code - Chat	https://www.figma.com/proto/lr7BPu7e6Iem8K9jR6mKMQ/Design-Probes-(L)---P3?type=design&node-id=26-90&t=UGpUM26rZGFXwlvj-1&scaling=contain&page-id=0%3A1&starting-point-node-id=26%3A90&mode=design
P2	Linear	Template - VbD - Example - Code - Chat - Shelf	https://www.figma.com/proto/HnK3uUM3oixyj9Wfncggpa/Design-Probes-(L)---P2?type=design&node-id=26-90&t=tfqqvtGWINE23R3a-1&scaling=contain&page-id=0%3A1&starting-point-node-id=26%3A90&mode=design
P1	Linear	Shelf - VbD - Template - Chat - Code - Example	https://www.figma.com/proto/qCXq1ppjIVYhMmcTedTFRa/Design-Probes-(L)---P1?type=design&t=b9IhXeOdrv19FMsw-1&scaling=contain&page-id=0%3A1&node-id=26-90&starting-point-node-id=26%3A90&mode=design
