# Supplementary material for: Understanding Visualization Authoring Techniques for Genomics Data in the Context of Personas and Tasks
Source: IEEE Trans Vis Comput Graph. Author manuscript; Available in PMC 2025 Mar 4. (PMC11875953; doi:10.1109/TVCG.2024.3456298)

- Participant characteristics
    - P10
        - High computational skills
        - Leaning toward the conventions in existing tools (e.g., click on a template)
- Training
    - I think it takes more time on VbD. Might affect the results.
    - "[Chat Input] is so much simpler than the previous one (VbD)" (P4)
- Test
    - P1 made a mistake on Q10 that files in the video looks like a gallery instead of files (confirmed after the test)
    - Mistakes on Q11 (P6, P7)
        - P6 recognized higlass as shelf
    - Shelf vs. Template
        - P1 was a little bit unclear in Q7
        - P4 thought shelf configuration (Q4 and Q7) are template based. Seemed to be confused distinguishing between the two. Seems to be clear after clarification. → actually, not very clear.
        - P9 selected Excel the template (Q7)
            - Confused by Excel
        - P10 selected Excel the template (Q7)
            - Confused since it is a Excel and did not see the example closely
        - P11 selected HiGlass the shelf (Q11)
        - P12 selected Excel the template (Q7)
    - Q13
        - P11 selected example upload
- Observed Tendency
    - 👍Advantages of Shelf
        - For basic customizations (or for little changes) → shelf construction (P1)
            - "Not complicated enough … to use chat input" (P1)
    - 👎Disadvantages of Shelf
        - "Anything beyond bar chart and line plot, it is difficult to describe in the system by dragging" how to achieve… (P6)
    - 👍Advantages of Template
        - "Easiest for me" (P2)
        - "Pretty basic" would be supported as templates (P5)
        - "see alternative options" "give you an idea" (P5)
        - Effect of preview
            - "you do not know what it should be called" (P6)
        - "Having names list out" (P9)
    - 👎Disadvantages of Template
        - "Not sure if a template can incorporate the set of coordinates" (P2)
        - Easy to use for simple filtering (P4)
        - I want "definite control" (P5)
    - 👍Advantages Code
        - Scalability, i.e., repeat for thousand of samples (P5)
        - "Keep track of what has been done" (P5)
            - P5 did not consider Code as the most preferred one, but considered it as an important feature to have.
        - "To do something that is not standard" (P10)
        - "Ultimate way to customize things" (P11)
    - 👎Disadvantages Coding
        - "Cumbersome … I don't love coding layout" (P3)

- 👎Disadvantages of VbD
    - "Reluctant to use visualization by demonstration" (P1)
    - "Not sure what I would get" (P1)
    - "Unsure how I would communicate with the system" (P1)
    - "Not sure how useful it is" … "maybe …" (P2)
    - "Struggling to see how I would demonstrate" (P3)
    - "Struggling to think about how I would do [demonstration]" (P3)
    - "Might be difficult to provide fine-grained customization" (P3)
    - "Not sure if I can exactly demonstrate what I want" (P3)
    - "I would use only if I am really familiar with the system" "too difficult" (P4)
    - "I wouldn't try it as a first" "don't know how to communicate" (P4)
    - "It's so different that what I have been doing" "I don't know what to expect" (P5)
    - "These (VbD and Example Up.) are farthest (or fuzziest) than what I am used to" (P5)
    - "How can I be sure I can get it" (P5)
    - Would be appropriate for "certain types of persons … you can draw really well" (P7)
    - "Not the first thing I would do" (P7)
    - "I have no idea where to begin in this case" (P7)
    - "awkward … do all gestures" (P10)
    - "I don't see how I describe it," "As a user, I don't know how to demonstrate it" (P13)
    - "No high granularity" (P13)
- 👍Potentially useful use cases of VbD
    - Create a relationship between views, i.e., overview + detail (P1)
    - Filter out reads by drawing, if it is clearly shown in the visulaization (e.g., white reads that represent zero quality) (P3)
    - Maybe for "really particular cases" (P5)
    - "Fun" "would be very fun" (P9)
    - "YOu list to have a full list of things that you can do" (P12)
- 👍Advantages of Chat
    - "You can specify all in one" (i.e., visualization type and coordinates) (P3)
    - "use publically available dataset" (i.e., download a data and upload it automatically to the system) (P3)
    - More fine-grained customization (P2)
        - "Color only chromosome 2"
    - "Easiest way to …" (P4)
    - Scalability
        - (clone task) , i.e., repeat for thousand of samples (P5)
        - (arrangement task) Order them based on something (P5)
    - "Reliable" (P8)
    - "describe the problem," "Oh, this is too crowded" (P10)
- 👎Disadvantages of Chat
    - "not perfect" (P3)
    - "bit of the stress of fully using ChatGPT", "worry about some of the stuffs" (P3)
    - I don't expect that AI will … and "I don't want to spend too much time fixing it" (P4)
    - "would be tricky for AI to expect what I want" (P4)
    - concern of the server, "privacy issue", sending data to the server for AI (P4)
    - "exact way to defining something" is not easy with chat (P10)
    - "Chat GPT needs to know which part of the data to use" (P13)
    - "I don't like the idea of chat input to stand alone" (P13)
- 👍Advantages of Example
    - "good starting point … but not for the entire process" (P3)
    - "It is a specific [visualization]," "It is so painful to create those figures (in papers)" (P2)

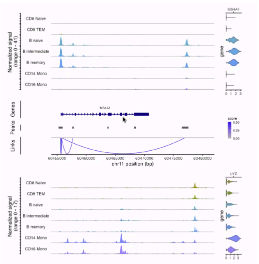

            - [redacted], link, link2
        - Reproducing examples (e.g., clone) (P2)
        - "Really cool and useful" (P3)
        - "Too much time" (P4)
        - "If I want something like fancier" (P5)
    - 👎Disadvantages of Example
        - Questionable "how much it will understand the properties in my plot" (P1)
        - "Still unclear in terms of the use case" (P2)
        - "Little bit clunky" (P3)
            - because have to download the file and upload it
        - "Slow," "too much effort" (P4)
        - "I can imagine frustration" (P5)
        - "I am not sure … how to transform data …" (P6)
        - "only apply to certain scenario" (P9)
    - Supporting multiple modalities in a single tool is…
        - "fluent" (P2)
        - "cool to see both of things[terminal and UI] in a same system" (P2)
            - "Seamless connection" – related to the *Integration*
            - P2 normally does it on a terminal and then uploads in a different system
        - "Definitely see the value," "useful" (P2)
        - "100%" useful! Genomics visualization is too complex to use only one modality (P5)
        - Normalize the score (chat) and visualize (shelf) (P6)
            - Working on the data with one modality and visualize with another
    - Using multiple modalities for a single task is…
        - "Combine Example with Code would be really cool," "make my job a lot easier," "sometimes you have an idea, but it is hard to code it" (P3)
- Results
    - Maybe, we need to revisit Template and Shelf and see if their understanding was clear when describing one of them for selecting an option for each question.
    - Q1
        - Code (P1, P2, P4, P5, P7, P8, P9, P10, P12, P13)
            - "More explicit" (P12)
            - "I like to have an agency of manipulating the data," "my comfort zone" (P13)
        - Chat (P2, P3, P4, P7, P8, P9, P10, P12, P13)
            - "Easily express" (P7)
            - "Compute the coverage across the bam files using sam tools" (P7)
            - "Keep all reads with the mapping quality over 30" (P7)
            - "filter quality score by greater than 30" (P12)
        - Template (P4, P5, P6, P10)
            - But want to "keep track of what I did" "save what I did" (P5)
            - Usual in genome browser, tedious to do coding, "such a standard option in genome browser" (P10)
        - Shelf (P4, P6)
            - Drag to do a calculation (P6)
        - VbD (P3, P11)
            - Rearrange bars to make them coverage bar (P11)

        - Chat + Code (P7)
            - Ask chat to see code and then code one my own (P7)
    - Q2
        - Template (P2, P3, P4, P5, P6, P7, P8, P9, P10, P13)
            - "easy" (P6)
        - Chat (P3, P4, P5, P9, P11, P12, P13)
            - "Add specific region of the genome" (P9)
            - "Can you extract the coverage for this position from file, and display as a bar chart" (P9)
            - "I have zero" … "easiest and less effort" (P11)
        - Shelf (P4, P7, P8, P10, P12, P13)
            - "Might be faster" (P8)
            - "y value being the y axis" (P10)
            - "I have to think about how to … what my x-axis is, what my y-axis is, …" (P12)
        - Code (P2, P4, P7, P9, P10)
            - "compute the score and then" "more control" (P10)
            - “most familiar” (P2)f
        - Example (P3, P5, P12)
            - "I normally draw what I want the figure to look like," "Pretty natural" "something that what I do" (P5)
        - VbD (P2, P9)
            - "Select the region of interest" (P9)
            - “could be powerful” (P2)
        - Chat + Code (P1)
            - asking it to change the bin size (P1)
    - Q3
        - Code (P3, P5, P7, P9, P10, P11)
            - "Gives me the control of the exact color code" (P10)
        - Chat (P2, P3, P4, P6, P8, P12, P13)
            - "Very straightforward to translate" (P6)
            - "Seems like the easiest" "Cosmetic changes seem to be suitable for code inputs" (P8)
            - "Change the color to blah blah blah" (P13)
        - VbD (P4, P6, P8, P9)
            - Drag the interval (P8)
            - Drop a color like in palette (P8)
            - Draw a region (P9)
            - Draw the bar to halp (P13)
        - Shelf (P1, P5, P6, P11, P12)
            - **"Plotly … teaching myself to use shelf-construction and then export the code to understand the code" (P11)**
        - Template (P2, P9)
        - Chat + Code (P5)
            - "Change the color" and then go to code for adjust (P5)
        - Other (P7, P10)
            - Right click to change color (P7, P10)
                - "if too custom …" that is not supported, the would prefer code (P7)
                - Palette (P13)
        - ~~VbD (or Other?) (P2) – not sure if need to include~~
    - Q4
        - Template (P1, P2, P3, P4, P5, P6, P7, P8, P9, P10, P11, P13)
            - "if it's basic, then" template would be good (P5)

            - "that's what what is exposed to" (P6)
            - "biased to software used" (P10)
            - "being able to test a lot of things" "being able to click quickly" (P11)
        - Shelf Configuration (P2, P3, P8, P12)
            - "Least amount of clicking" (P12)
        - Code (P3, P4, P10, P13)
        - Example (P3, P6, P9)
            - For something that is "very very specific style" of visualization (P6)
        - VbD (P6, P8, P9)
            - "Draw the circle" (P6)
            - "If so complex … draw rather than describing it (in textual representations)" (P8)
            - "Draw line, tracing the top of the plot" "instinct" (P9)
        - Chat (P3, P10, P13)
        - Option
            - Suggestions, i.e., "possible layouts" (P8)
    - Q5
        - Shelf (P1, P2, P3, P4, P5, P7, P10, P11, P13)
        - Code (P2, P3, P7, P9, P11, P12, P13)
            - "within this region, what's the count of …" (P9)
            - "if you want a certain number of things…" (P11)
            - "Copy and paste the code and change the input of the data" (P13)
        - Chat (P1, P3, P5, P6, P7, P9, P12)
            - "Co-pilot" (P7)
            - "within this region, what's the count of …, and display it" (P9)
        - VbD (P5, P8, **P12**, P13)
            - Add a label, put a track by drawing (P5)
            - Drawing lines inside the inner track (P13)
        - Template (P6)
        - Example (P6)
            - "A way to make sure, you are uploading the right track" "Look just like this" (P6)
            - Good for "detailed output, or something that is hard to implement" (P6)
            - "Extra step" (P9)
        - Chat + VbD (P8)
            - Use chat input and then draw a line to let the system know (P8)
        - Chat + Code (P4)
            - Use code to adjust (P4)
        - Example + Chat (P3)
            - "Interesting idea" to combine modalities (P3)
        - Chat + Template/Shelf (P9)
        - Other
            - Automatically, populate the visualization by dragging the data file (P10)
    - Q6
        - Example (P2, P3, P4, P6, P8, P9, P10, P13)
            - reproduce the example (P2)
            - "Screenshot this, and use as the starting point" (P3)
            - "Sketch a whole genome overview …" (P8)
            - Screenshot of this (P9)
            - PPT (P13)
        - Chat (P2, P3, P4, P5, P7, P9, P12)
            - "I don't see how I should do that … so I will probably use [chat input]" (P4)
            - "do the same visualization for all the samples" (P5)

        - "I never seen something like it before" (P7)
        - "I want it for different region" (P9)
        - One prompt er track (P12)
    - Code (P4, P5, P7, P9, P10, P12)
        - "Easy … because … can reuse the entire code" (P4)
        - "Very good use case to use code input" (P7)
        - "I had a lot of trouble linking views …" (P10)
    - VbD (P1,P2, P5, P8, P10, P11, P13)
        - "click on some other sample and clear another sample for me" (P2)
        - "draw the link between views" (P5)
            - Some interesting comments that we can revisit!
        - "Draw the zoomed out version" (P8)
        - Selecting a region "I want to zoom into that region in a visualization" (P10)
        - **Like Mac screenshot, select a subsection, and then click on the "create a new view and zoom into this section"** (P11, P1)
    - Shelf (P3)
        - "easily toggle between different datasets" (P3)
        - "change the input file and drag and drop" data (P5)
            - But does not work for thousand samples
    - Template (P6)
        - "If it have all the options" (P6)
    - **VbD + Shelf (P11)**
        - Like Mac screenshot, select a subsection, and then click on the "create a new view and zoom into this section" and then use shelf configuration to remove tracks of not interest (P11)
    - Other
        - Drag and drop (P12)
- Q7
    - VbD (P2, P3, P6, P8, P9, P11)
        - Drag the plots to a certain area with a mouse, and "snap" (P2)
        - "Start dragging things on top of each other" "Powerpoint" "align" (P3)
        - "Click and drag them … most natural way to do it" (P6)
        - "draws arrows" "[VbD] sounds more fun" (P9)
        - "Stick and align to the position" (P11)
    - Chat (P1, P2, P3, P5, P9, P12, P13)
        - order them based on something (P5)
        - "I would like all this plot to be horizontal" (P9)
    - Code (P2, P4, P9, P10, P12, P13)
        - "Really simple with code" (P4)
        - Edit the parameter (P9)
        - "determine the size of the grid" (P10)
    - Shelf (P4, P5, P6, P11, P13)
        - **Need to confirm what people meant by this!**
        - dragging the data to a specific cell in a table template (or zones)? (P11)
    - Example (P3, P5, P8, P13)
        - "quickly draw them on a paper" (P5)
        - "Draw" (P8)
    - Template (P7, P10)
        - Already provide some options (P7)
    - Other
        - Select all views and then click on "align all to abc" (P10)
        - Drag and drop (P13)
- Q8 (Genome Annotation Task)

        - Overall similarity
        - Shelf (P3, P4, P6, P8, P11, P13)
            - "Provide additional data" (P8)
        - Chat (P3, P5, P6, P9)
        - Code (P4, P9, P10, P12)
            - "Copy and change … to what I want" (P4)
            - Average coverage of each cell type (P9)
            - Want to be exact (genomics) coordinate. "Exact way to defining something" (P10)
        - VbD (P5, P7, P10, P13)
            - Basic annotation (P5)
            - based on whether the annotation would be linked with data or not (P5)
            - "Definitely do with [VbD]" (P7)
                - "Adding a label"
            - Draw annotation for highlighting certain regions (P10)
                - For collaborate (e.g., write a comment for share)
            - Adding annotations (e.g., adding arrows and labels) (P13)
        - Template (P6)
        - Example + Chat input + VbD (P2)
            - "in a certain style", "see a figure in another paper" (P2)
            - Chat input to adjust style, e.g., move labels
            - VbD to rearrange labels and etc
        - Drag and drop the data + select the plot with VbD + shelf (P12)
        - Other
            - Drag and drop the edge of a circos track (P13)
- Discussion Points
    - Interestingly, all participants did not stick to a single modality across all tasks.
    - Modalities by Tasks
        - Can we draw an overall workflow of constructing visualization and come up with relevant modalities that are preferred/effective for each part?
        - Chat
            - "good way to do for the final step" (e.g., fine tunning) (P6)
            - "When I am like in zeros" and to all things (P11)
    - Value of multi-modality
        - Clear task separation
            - Working on the data with one modality and visualize with another are preferred (P6)
        - "Initially doubt about VbD" (P8)
        - Chat support, in addition to others, such as Chat + Example (P8)
        - "Having flexibility in the system" (P10)
    - Considerations for selecting modalities
        - Given tasks
            - "easy to difficulty of tasks" (P8)
            - "some kinds of calculations" (P9) — Code/Chat
            - "visually something" (P9) — VbD
            - "When I am close to this, … I don't want to … save me time … rather click through it" (P11)
            - How fine grained you want to do something
                - "but you cannot have infinite options" (P11) — shelf
        - Reproducibility
            - "in existing tools, applying the same thing to another is missing" (P5)
        - Scalability of repeated interactions
            - Applying for thousands of samples (P5)

            - Apply data to a single visualization. Code is good. Would be good to have other modalities for this. (P13)
        - Difficulty in communicating with the system
            - Sometimes, chat is difficult since it is difficult to explain in language, and communicating with 'visual' for 'visualizations' (like Example Upload) could be easier (P5)
            - "How to communicate, and how to communicate correctly" (P6)
            - "I don't really know…. very very cumbersome to describe it" "Could be very long description" (P6) — BAM tracks
            - "You need to be okay drawer" (P6) — VbD
            - "has to understand what you are doing" (P8) — VbD
            - "not having to\
            - "I don't know how to phrase that to chat input" (P13)
            - "less freedom .. unless very specific … I rather do it myself" (P13) – code
        - Automatic tool is not perfect
            - "vaguest thing about the VbD, Example, and Chat are that … **there is a gap** between what you want to create vs. what you would get" (P6)
            - Templates are not generally expected to give perfect vis. design (P7)
            - "Typically Chat-GPT get wrong" code (P8)
        - Transparency
            - "Very little transparency" "errors … in the data … that you are not aware of" (P6)
        - Efficiency
            - "[Example upload] and [VbD] would takes too long" (P8)
            - "little more clicking" on GUI (P10)
            - "code … annoying" kind to write in words before seeing. "switch between options" (P11)
- Important question to clarify!
    - Would zoom interaction on ideograms (i.e., selecting a region to move to that region) VbD?

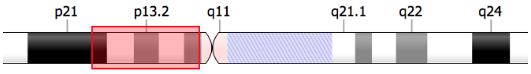

Supplement: tvcg-3456298-mm [file NIHMS2039885-supplement-tvcg-3456298-mm.zip › tvcg-3456298-mm/study2_notes.pdf]
