# Supplementary material for: Understanding Visualization Authoring Techniques for Genomics Data in the Context of Personas and Tasks
Source: IEEE Trans Vis Comput Graph. Author manuscript; Available in PMC 2025 Mar 4. (PMC11875953; doi:10.1109/TVCG.2024.3456298)
Supplement: tvcg-3456298-mm [file NIHMS2039885-supplement-tvcg-3456298-mm.zip › tvcg-3456298-mm/study2_pilot_protocol.pdf]

Study 2 Protocol

[1 min] Pitch of study aims and setup ← 00:03:06

- [ ] Need to ensure that the link is sharable

[17 min] Training Phase:

(15 min) Showing videos & interacting with the Figma training prototype

1. **Template-based**: Select a chart from a gallery of chart types. After selection some customization is possible. An example system of this modality is Microsoft Excel or Tableau Show Me (Satyanarayan et al., 2019) ← 00:06:58.97
2. **Shelf construction**: Instead of choosing a predefined chart template, the user can drag and drop data fields onto a drop zone (i.e., the shelf) to specify how the data should be visually encoded; the system then renders a valid visualization. This modality provides somewhat more flexibility over templates. An example is Tableau (Satyanarayan et al., 2019) ← 00:08:43.49 (perhaps, explain how the bar chart is shown with the "value" field)
3. **Direct manipulation**: Users can directly add or edit graphical elements on a canvas to construct a visualization (Satyanarayan et al, 2020/ Liu et al., 2019). Manipulations can be performed using mouse or pen interactions. The modality is inspired by vector graphics editors such as Adobe Illustrator and SketchVis for pen interaction (Browne et al., 2011) ← 00:10:11.22 (use a video with pen interaction on an existing vis/chi paper?)
4. **Chat input**: Users create a visualization through a conversational interaction with a natural language interface (Wang et al., 2022). Examples of this modality are VisTalk (Wang et al., 2022), Eviza (Setlur et al., 2016), and ChatGPT. ← 00:12:01.39 (Bug getting to a previous slide)
5. **Code input**: An interface through which a user can interactively code up or textually specify a visualization construction. An example is Jupyter Notebook. ← 00:13:00.96 (bug broken and Jupyter not shown)
6. **Example upload**: Contrary to choosing a chart by template, a user can upload an example in the form of code, an image or a sketch to construct a visualization. After upload customization is possible. For sketch upload, an example is Sketch2Vis (Teng et al., 2021). ← 00:13:33.79

(2 min) Test to collect participant's understanding of the modalities

- Share this google form: https://forms.gle/1w1UbwAVvnfTtkHW6

[40 min] Test Phase with Design Probes:

(30 min) Interaction & thinking out loud with 8 probes

- Example data set?
- Two layouts (linear or circular) serve as starting points for the probes. A statement about justification why these different layouts matter.
- Between-study setup: participants are assigned to either linear (L) or circular (C) based on their experience measured in study 1.
- Flow of probes: all sessions start with probe 1 (data transformation) and then continue to either a series of linear (L2→L8) or circular (C2→C8) probes.
- The order of the modalities is randomized per participant but consistent for each probe of that participant.
- Elicitation session: we make clear that tasks are merely examples, but they are free to choose something else that is more closely related to their workflow
    - Each probe has clear instructions for the tasks and options
        - “Please pick one …
        - “Pick one or more modality”
- (24 min) Sequence of probes (L) or (C)

**Probe**	**Duration**	**Task**	**(L) Linear Layout**	**(C) Circular Layout**
1	3 min<br><br>(~2:38)	T1: Transform data<br>- Calculate the coverage score for the given BAM file<br>- Filter out low quality reads		
2L	3 min	T2: Create track that visualizes:<br>- Bar chart of the coverage score (BAM)<br>- Line chart of a BigWig file<br>- …	✔	
3L	3 min	Select one of the following tasks that you perform frequently and select one or multiple interaction modalities to perform the task:<br>T3: Customize the visualized track<br>- Change the color coding of the visualized track<br>- …<br>- …	✔	
4L	3 min	T4: Modify the visualization<br>- Change the visualization type to line plot<br>- Change the visualization type to scatter plot<br>- Change the layout to circular<br>- Change the layout to Hilbert	✔	

5L	3 min	T5: Add track(s) to the view<br>- Add a track visualization of the SNV diversity in the same ROI	✔	
6L	3 min	T6: Create multiple views<br>- clone this visualization but but in a different ROI<br>- add a whole genome overview and create a brush to link to the detailed view	✔	
7L	3 min	T6: Change the arrangement of the views<br>- Vertical juxtaposed <-> horizontal<br>- Juxtapose <-> Superimpose	✔	
8L	3 min	Create a custom genome annotation and add it to the view(s) to use this visualization as a figure in a paper	✔	
2C	3 min	T2: Create a bar track to visualize the coverage score		✔
3C	3 min	T3: Customize the visualized track<br>- Change the color coding of the visualized track<br>- …		✔
4C	3 min	T4: Change the visualization type of this track (e.g., from bar to line)		✔
5C	3 min	T5: Add track(s) to the view<br>- Add a track visualization of the SNV diversity in the same ROI		✔
6C	3 min	T6: Create multiple views<br>- clone this visualization but but in a different ROI<br>- add a whole genome overview and create a brush to link to the detailed view		✔
7C	3 min	6: Change the arrangement of the two views<br>- Vertical juxtaposed <-> horizontal<br>- Serialized <-> Parallelized		✔
8C	3 min	Create a custom genome annotation and add it to the view(s) to use this visualization as a figure in a paper		✔

- (6 min) Sequence of probes (L) or (C)
  After one set of probes (L) or (C):
    - "I see that you mainly used modality …, what is the reason for this?"

- "I see that you hardly used modality …, what is the reason for this?"
- If multiple are used: what is the reason for combining modalities?
- If participants indicated wanting additions / modifications to the modalities: what is the reason for approaching the task with this custom modality?

(5 min) Survey to collect participant's experiences with the modalities

- On a scale from 1 (Not at all) to 7 (Extremely), how **familiar** were you with each modality for constructing visualizations **before participating** in this interview
- On a scale from 1 (Not at all) to 7 (Extremely), how **likely** would you use each of the modalities for constructing visualizations

(5 min) Follow-up interview on general experience

Interview guide:

- What was your general experience interacting with the different modalities?
- Which modalities surprised you the most?
- Which modalities did you find most / least useful?
- Which modalities would you most / least likely use?
- What is your opinion about having multiple modalities for construction of visualizations?

[2 min] Thanks and Closing

Participants recruited

Demographics of participants

Participants who were involved in study 1 are again invited for the second study. Furthermore, we aim to recruit a diverse group of users. Diversity is evaluated by:

- Main job title / role: PhD & PostDoc, Research Scientist, Software Engineers / Scientific Programmers
- Lab / Work affiliation:
    - HMS
    - WUR
    - Other (Broad Institute, TGEN, …)
- Type of data analysis
    - Human genome, plant genome, bacterial/virus genome
    - Sequence variation, structural variation, epigenomics, …

Number of participants

3 groups (PhD+PD vs SE vs RS) x 2 setups (Linear vs Circular fist) x 2 (from each group at least two people follow the same setup) = **12 participants**
