# Supplementary material for: Understanding Visualization Authoring Techniques for Genomics Data in the Context of Personas and Tasks
Source: IEEE Trans Vis Comput Graph. Author manuscript; Available in PMC 2025 Mar 4. (PMC11875953; doi:10.1109/TVCG.2024.3456298)
Supplement: tvcg-3456298-mm [file NIHMS2039885-supplement-tvcg-3456298-mm.zip › tvcg-3456298-mm/study2_slides.pdf]

User Interview Genome-mapped Data Visualization

Follow-up study aim and setup

Study aim

To understand whether, how and why **different users** may use different **interaction modalities** for **for different tasks** of the visualization construction workflow

Interaction modality : a way how a human can send information to a machine (feedback loop)

Procedure

(20 min) Introduction of modalities

(40 min) Interaction with probes

Questions & Reflection

Recording request

Do we have permission to:

- record zoom meeting while you share your screen?
- permission to auto transcribe zoom meeting?
